# Mitofusin 1 is required for female fertility and to maintain ovarian follicular reserve

**DOI:** 10.1038/s41419-019-1799-3

**Published:** 2019-07-22

**Authors:** Man Zhang, Muhammed Burak Bener, Zongliang Jiang, Tianren Wang, Ecem Esencan, Richard Scott III, Tamas Horvath, Emre Seli

**Affiliations:** 1 Department of Obstetrics, Gynecology and Reproductive Sciences, Yale School of Medicine, New Haven, CT 06510 USA; 2 Department of Comparative Medicine, Yale School of Medicine, New Haven, CT 06520 USA; 3 Present Address: AgCenter, School of Animal Sciences, Louisiana State University, Baton Rouge, LA 70803 USA; 4 Present Address: Foundation for Embryonic Competence, Basking Ridge, NJ 07920 USA

**Keywords:** Cell proliferation, Oogenesis

## Abstract

Mitochondria are dynamic organelles that continually adapt their structure through fusion and fission in response to changes in their bioenergetic environment. Targeted deletion of mitochondrial fusion protein mitofusin1 (MFN1) in oocytes resulted in female infertility associated with failure to achieve oocyte maturation. Oocyte-granulosa cell communication was impaired, and cadherins and connexins were downregulated, resulting in follicle developmental arrest at the secondary follicle stage. Deletion of MFN1 in oocytes resulted in mitochondrial dysfunction and altered mitochondrial dynamics, as well as accumulation of ceramide, which contributed to increased apoptosis and a reproductive phenotype that was partially rescued by treatment with ceramide synthesis inhibitor myriocin. Absence of MFN1 and resulting apoptotic cell loss also caused depletion of ovarian follicular reserve, and a phenotype consistent with accelerated female reproductive aging.

## Introduction

Mitochondria have the ability to adapt their shapes through fusion and fission (collectively termed mitochondrial dynamics) in response to changes in their metabolic milieu^1^. Mitochondrial dynamics are essential for mitochondrial energy metabolism and stress response^2^, and seem to play a key role during oocyte and pre-implantation embryo development, as mitochondria become more elongated^3^, with higher number of cristae and lower density matrix^4^.

Fusion is a mechanism that allows one mitochondrion to compensate for a functional defect in another by sharing transfer RNAs, ribosomal RNAs, and proteins^5^, while fission may occur when mitochondria are damaged, accumulate deleterious components, or are subjected to high levels of cellular stress. Fusion is mediated by mitofusin 1 (MFN1) and mitofusin 2 (MFN2) in the outer mitochondrial membrane and by optic atrophy 1 (OPA1) in the inner mitochondrial membrane^6^. A number of proteins have been implicated in controlling mitochondrial fission, including dynamin-related protein 1 (DRP1)^7^.

The fundamental necessity for mitochondrial fusion and fission processes in the maintenance of cellular homeostasis has been demonstrated in a number of cell types, including fibroblasts, epithelial cells, and neurons^8–11^, and confirmed by embryonic lethal phenotype of *Drp1*, *Opa1*, *Mfn1*, and *Mfn2* mouse knockout models^12–14^. Recent studies also suggest an important role for mitochondrial dynamics in female reproduction. siRNA-mediated knockdown of mitochondrial fusion gene *Mfn2* in immature oocytes results in a decline in oocyte maturation^15^, while oocyte-specific knockout of mitochondrial fission factor *Drp1* results in defective follicular maturation and female infertility^16^.

In this study, we aimed to investigate the role of MFN1 in female fertility and ovarian function. We found that oocyte-specific targeted deletion of *Mfn1* causes infertility with defective follicle development and lack of oocyte maturation. These defects were associated with impaired mitochondrial function and dynamics, and accumulation of ceramide in oocytes; reproductive phenotype could partially be rescued with ceramide synthesis inhibitor myriocin. Importantly, in the absence of MFN1, follicular depletion was accelerated, consistent with a phenotype of diminished ovarian reserve.

## Results

### *Mfn1* is required for female fertility, oocyte maturation, and follicle development

Oocyte-specific *Mfn1* knockout (*Mfn1*^*−/−*^) mice were generated by crossing floxed *Mfn1* mice to transgenic mice expressing *ZP3-Cre*^17,18^, resulting in the loss of *Mfn1* mRNA expression in the oocyte, but not in granulosa cells (Fig. S1A). Mature (8-week-old) *Mfn1*-deficient female mice were viable, however, their ovaries were significantly smaller in size (1.65 ± 0.08 vs. 4.8 ± 0.75 μm^2^, *p* *<* 0.05) and weight (2.017 ± 0.1352 vs. 3.667 ± 0.1856 mg, *p* *<* 0.01) compared to WT (*n* = 3–6 for each genotype) (Fig. S1B, S1C and S1D).

**Fig. 1:**
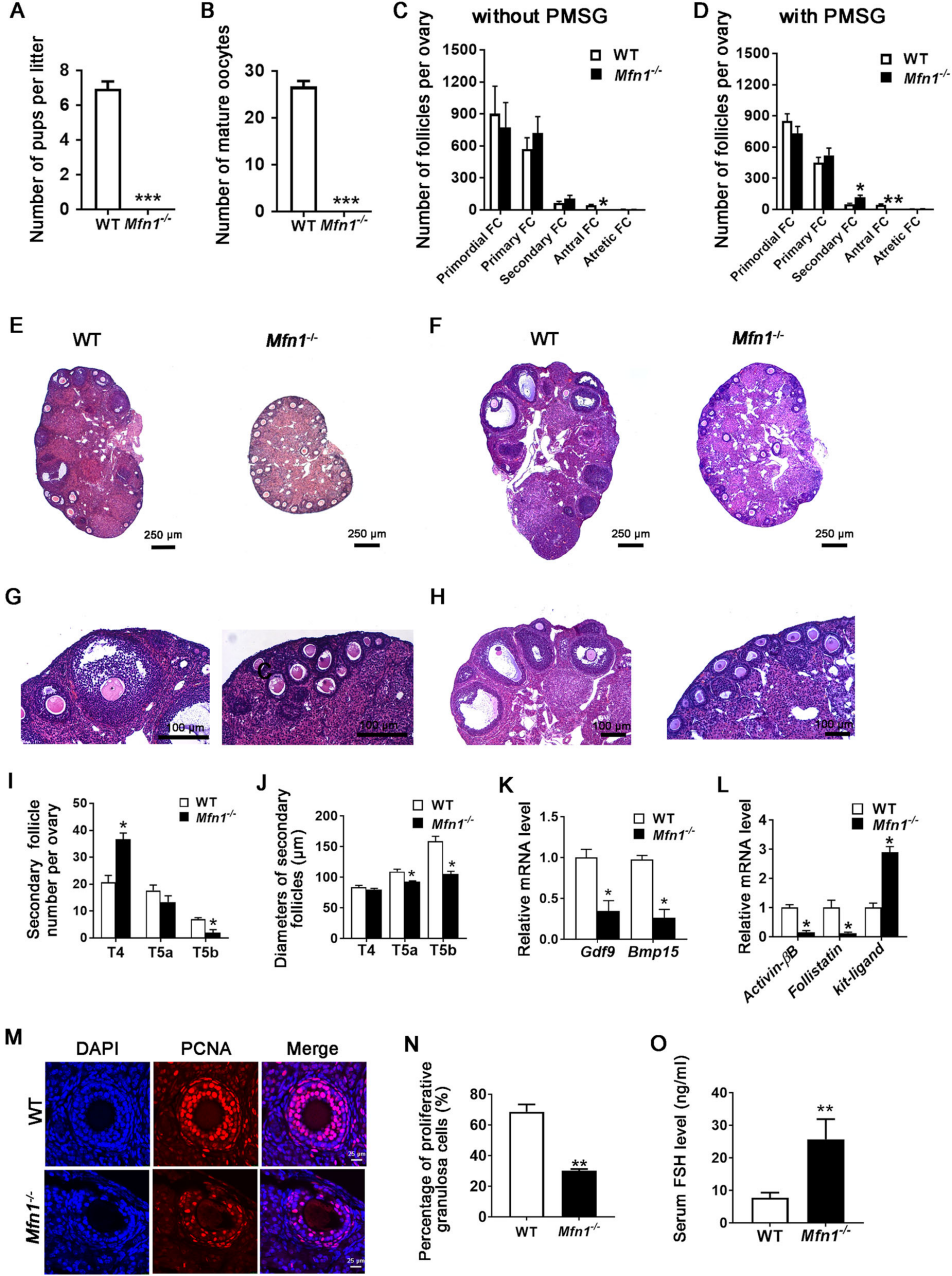
*Mfn1*^*−/−*^ mice are infertile, with defective oocyte and follicular maturation. **a** Fertility of *Mfn1*^*−/−*^ and WT female mice (8-week-old) was assessed by mating with WT males of proven fertility (male/female; 1:2) for 12 weeks. The average number of delivered pups per litter per mouse was recorded. Data presented as mean ± SEM. *N* = 7 for each genotype; ***p < 0.001 vs. WT using *t*-test. **b** *Mfn1*^*−/−*^ and WT female mice (8-week-old) were superovulated by PMSG and hCG, and the number of mature (MII) oocytes collected from oviducts was counted. Data represent mean ± SEM. *N* = 6 for each genotype; ***p < 0.001 vs. WT using *t*-test. **c** and **d** Ovaries from *Mfn1*^*−/−*^ and WT mice (8-week-old) primed with PMSG (**d**) or unprimed (**c**) were fixed, embedded, sectioned, and stained with hematoxylin and eosin. Follicle counts using 3 mice ovaries for each genotype were performed. No antral follicles were found in *Mfn1*^*−/−*^ ovaries, with or without PMSG stimulation. **e**, **f**, **g** and **h** Representative micrographs ovarian sections from *Mfn1*^*−/−*^ and WT mice (8-week-old) with (**f** and **h**) or without (**e** and **g**) PMSG stimulation. **i** The number of secondary follicle subtypes per ovary was quantified as described in Materials and Methods section (*n* = 4 mice per genotype). **j** The diameters of secondary follicle subtypes per ovary were measured (*n* = 4 mice per genotype). **k** Secondary follicle-enclosed oocytes and granulosa cells were collected from *Mfn1*^*−/−*^ and WT ovaries. qRT-PCR was performed to determine *Gdf9* and *Bmp15* mRNA expression in *Mfn1*^*−/−*^ and WT oocytes. **l** qRT-PCR analysis of *ActivinβB, Follistatin*, and *kit-ligand* in *Mfn1*^*−/−*^ and WT granulosa cells was performed. **m**, **n** PCNA immunofluorescence assays and the quantification of PCNA-positive granulosa cells in *Mfn1*^*−/−*^ and WT ovaries are shown. **o** Serum FSH levels in sera of *Mfn1*^*−/−*^ and WT mice (*n* = 7–11 per genotype). Data presented as mean ± SEM. *p < 0.05, **p *<* 0.01 vs. WT using *t*-test. T4: follicles with two layers of cuboidal granulosa cells, T5a: follicles with three layers of granulosa cells, T5b: follicles with many layers of granulosa cells but no antrum. Data represent mean ± SEM. *p < 0.05 vs. WT using *t*-test

To evaluate the fertility of *Mfn1*^*−/−*^ mice, we conducted a continuous mating study using sexually mature female mice (8-week-old, *n* = 7 for each genotype) and WT male mice (12-week-old) of proven fertility. After 12 weeks of mating, there were no pregnancies or deliveries in *Mfn1*^*−/−*^ mice, while WT female mice produced an average of 7 pups per mating (0 vs. 6.947 ± 0.429, *p* *<* 0.001) (Fig. 1a); both genotypes exhibited normal sexual behavior (assessed by the presence of a vaginal plug). In addition, *Mfn1*^*−/−*^ mice failed to produce mature (MII) oocytes (0 vs. 26.67 ± 1.202, *p* *<* 0.001) (Fig. 1b).

Assessment of serial ovarian sections revealed that *Mfn1*^*−/−*^ and WT mice ovaries had similar number of primordial, primary, secondary, and atretic follicles. However, no antral follicles were found in *Mfn1*^*−/−*^ ovaries (Fig. 1c, e and g). Arrested follicular development was also observed in the ovaries of *Mfn1*^*−/−*^ mice primed with PMSG (Fig. 1d, f and h).

### *Mfn1* depletion in oocytes inhibits ovarian secondary follicle growth

To determine the specific stage where the secondary follicle development is blocked in *Mfn1*^*−/−*^ mice, we quantified type 4 follicles (with two layers of cuboidal granulosa cells), type 5a follicles (with three layers of granulosa cells), and type 5b follicles (with many layers of granulosa cells but no follicle fluid) in *Mfn1*^*−/−*^ and WT mice, as described by Pedersen and Peters^19^. *Mfn1*^*−/−*^ ovaries showed few type 5b secondary follicles, while they had significantly higher number of type 4 follicles (Fig. 1i and Fig. S1E). In addition, type 5a and 5b secondary follicles were significantly smaller in size in *Mfn1*^*−/−*^ ovaries (Fig. 1j), and there were fewer PCNA-positive (proliferating) granulosa cells in *Mfn1*^*−/−*^ mice secondary follicles compared to WT (30.07% ± 1.135 vs. 68.57% ± 4.88, *p* *<* 0.01) (Fig. 1m, n). These data indicate that MFN1 depletion in oocytes blocks the transition of secondary follicles from two layers to multiple layers of granulosa cells.

To characterize the factors contributing to abnormal follicular maturation in *Mfn1*^*−/−*^ mice, we assessed the expression of genes that mediate communication between oocytes and granulosa cells using qRT-PCR. The expression levels of oocyte-specific genes *Gdf9* and *Bmp15*, which regulate follicle development and stimulate granulosa cell proliferation, were significantly lower in *Mfn1*^*−/−*^ oocytes (Fig. 1k). Furthermore, the expression levels of *ActivinβB* and *Follistatin*, which are implicated in the regulation of granulosa cell proliferation and follicle growth were lower in granulosa cells of *Mfn1*^*−/−*^ mice compared to WT (Fig. 1l), whereas *kit-ligand* expression, which promotes oocyte growth, was significantly increased (Fig. 1l). Consistent with granulosa cell dysfunction, serum follicle stimulating hormone (FSH) levels were higher (25.68 ± 6.19 vs. 7.719 ± 1.59 ng/ml, *p* < 0.01) in *Mfn1*^*−/−*^ mice compared to WT (Fig. 1o).

### Mitochondrial function is impaired in *Mfn1*^*−/−*^ oocytes

**Fig. 2:**
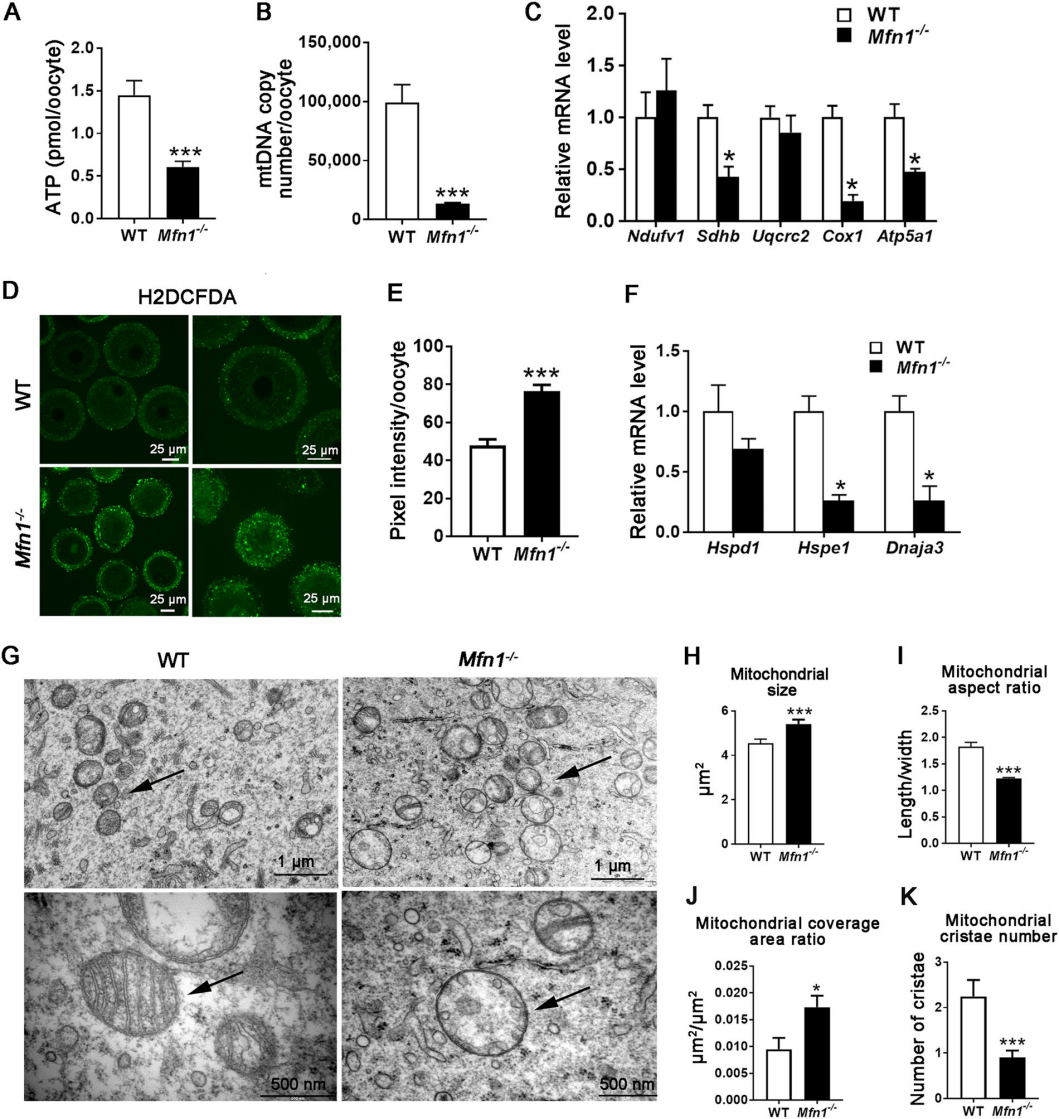
Mitochondrial function and dynamics are impaired in *Mfn1*^*−/−*^ oocytes. **a** ATP levels were measured in *Mfn1*^*−/−*^ and WT female mice (8-week-old). **b** mtDNA copy number was determined by qPCR in individual oocytes collected from *Mfn1*^*−/−*^ and WT mice. **c** Expression of electron transport chain genes was assessed using qRT-PCR in oocytes collected from *Mfn1*^*−/−*^ and WT mice. **d** ROS levels were measured in *Mfn1*^*−/−*^ and WT oocytes after treatment with H_2_O_2_. **e** Fluorescence intensity of Carboxy-H_2_DCFDA was used to measure ROS levels. **f** Expression of mitochondrial unfolded protein response (mtUPR) genes was assessed using qRT-PCR in oocytes collected from *Mfn1*^*−/−*^ and WT mice. **g** Representative electron microscopic photographs of oocytes from 8-week-old *Mfn1*^*−/−*^ and WT mice ovaries (*n* = 3 mice assessed in each group). Arrows show mitochondria. **h**–**k** Mitochondrial size (**h**), aspect ratio (mitochondrial length/ width) (**i**), coverage area ratio (mitochondrial area/cytoplasm area) (**j**), and number of mitochondrial cristae (**k**), in *Mfn1*^*−/−*^ oocytes compared to WT. Data presented as mean ± SEM. *p < 0.05, ***p < 0.001, vs. WT using *t*-test. ATP adenosine triphosphate, *Ndufv1* NADH dehydrogenase (ubiquinone) flavoprotein 1, *Sdhb* succinate dehydrogenase complex iron sulfur subunit B, *Uqcrc2* ubiquinol cytochrome c reductase core protein 2, *Cox1* cytochrome c oxidase subunit I, *Atp5a1* ATP synthase, H + transporting, mitochondrial F1 complex, alpha subunit 1

Next, we assessed mitochondrial function in *Mfn1*^*−/−*^ mice oocytes compared to WT. As *Mfn1*^*−/−*^ ovaries were devoid of antral follicles, secondary follicle-enclosed oocytes were collected for analysis in both *Mfn1*^*−/−*^ and WT mice. *Mfn1*^*−/−*^ oocytes had lower ATP levels (0.61 ± 0.07 vs. 1.45 ± 0.17, *p* *<* 0.001) (Fig. 2a), lower expression of mRNAs coding for electron transport chain (ETC) complex II (*Sdhb*), IV (*Cox1*) and V (*Atp5a1*) proteins (Fig. 2c), and higher ROS levels (76.52 ± 3.25 vs. 47.86 ± 3.206 pixel intensity, *p* *<* 0.001) (Fig. 2d, e), compared to WT. These findings were consistent with mitochondrial dysfunction in *Mfn1*^*−/−*^ oocytes. We also found that *Mfn1*^*−/−*^ oocytes had dramatically lower mtDNA copy number compared to WT (13,398 ± 870.4 vs. 99,108 ± 15,060, *p* *<* 0.001) (Fig. 2b), and decreased expression of mitochondrial unfolded response (mtUPR) genes *Hspe1* and *Dnaja3* (Fig. 2f), suggesting a defect in mounting a stress response. Furthermore, increased mitochondrial clustering was observed in *Mfn1*^*−/−*^ secondary follicle-enclosed oocytes compared to the homogeneous distribution of mitochondria in WT (Fig. S8).

Electron microscopy (EM) analysis showed that mitochondria in *Mfn1*^*−/−*^ oocytes were larger in size (5.409 ± 0.1975 vs. 4.538 ± 0.1941 μm^2^, *p* *<* 0.001), had increased mitochondrial coverage area (0.0173 ± 0.0022 vs. 0.0095 ± 0.0021 μm^2^/ μm^2^, *p* *<* 0.05), and a smaller aspect ratio (length/width) (1.226 ± 0.015 vs. 1.824 ± 0.081, *p* *<* 0.001) with a more rounded contour (Fig. 2g-j). Additional abnormalities in the mitochondrial internal structure including decreased number of cristae (0.90 ± 0.15 vs. 2.24 ± 0.36, *p* *<* 0.001) and formation of inner membrane vesicles were also observed in *Mfn1*^*−/−*^ oocytes (Fig. 2g, k). Collectively these data suggest that mitochondrial function and dynamics are severely impaired in *Mfn1*^*−/−*^ oocytes.

### Gene expression is altered in *Mfn1*^*−/−*^ oocytes

**Fig. 3:**
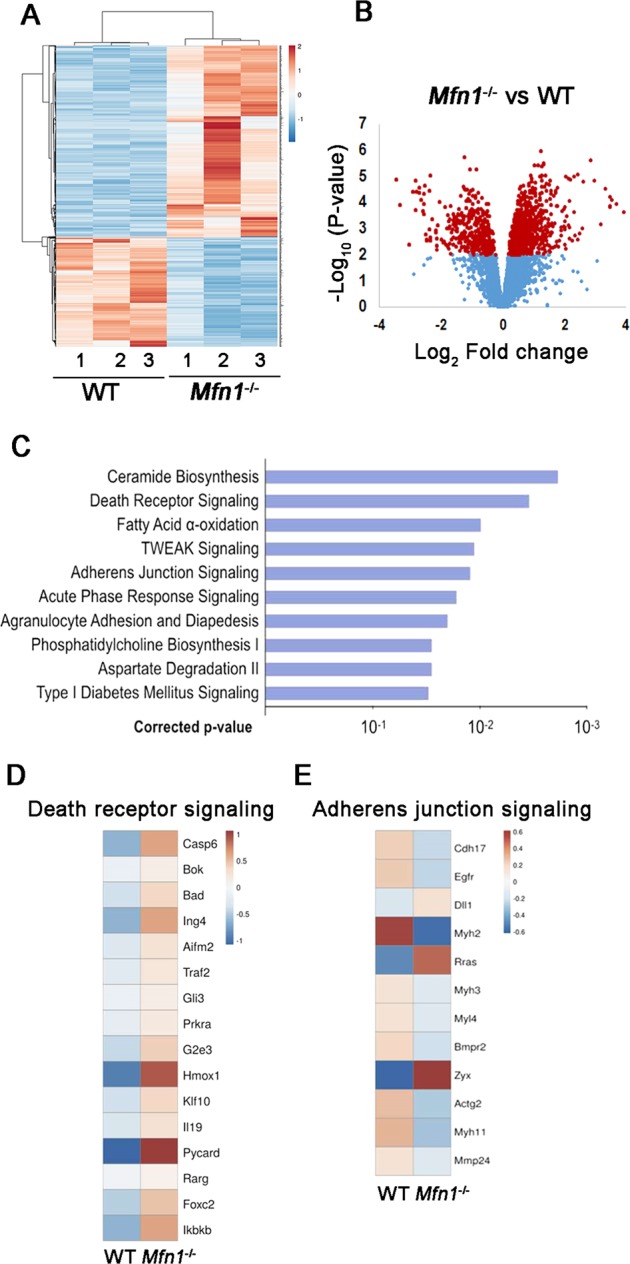
Gene expression is altered in *Mfn1*^*−/−*^ oocytes. **a** Heatmap illustration showing differentially expressed genes in *Mfn1*^*−/−*^ and WT secondary follicle-enclosed oocytes from 8-week-old mice. The color spectrum ranging from red to blue indicates normalized levels of gene expression from high to low. **b** Volcano plots for RNA-seq comparing *Mfn1*^*−/−*^ and WT oocytes. Red spot represents –log10 (*p*-value) ≥2; blue spot represents the –log10 (*p*-value) <2. **c** Pathway enrichment analysis in *Mfn1*^*−/−*^ oocytes compared to WT. **d**, **e** Heatmap illustration showing differentially expressed genes involved in death receptor signaling and adherens junction signaling in *Mfn1*^*−/−*^ oocytes compared to WT. The color spectrum ranging from red to blue indicates normalized levels of gene expression from high to low

To identify the genes and pathways affected by absence of MFN1 in oocytes, we adopted an unbiased approach with comprehensive genome-wide transcriptomic analysis. Hierarchal clustering of the differentially expressed genes partitioned into two distinct clusters to separate *Mfn1*^*−/−*^ and WT oocytes (Fig. 3a), suggesting high reproducibility of the sequencing data. A total of 982 genes were significantly differentially expressed (*p* *<* 0.05) in *Mfn1*^*−/−*^ oocytes compared to WT, with 654 upregulated and 328 downregulated genes (Fig. 3b). Pathway analysis of regulated genes indicated significant over-representation of elements involved in regulation of adherens junction signaling, death receptor signaling, and ceramide biosynthesis (Fig. 3c).

Hierarchal clustering of the differentially expressed genes involved in death receptor signaling partitioned into two distinct clusters to separate *Mfn1*^*−/−*^ and WT oocytes (Fig. 3d). qRT-PCR confirmed increased mRNA expression of death receptor signaling genes *Bad* and *G2e3* in *Mfn1*^*−/−*^ oocytes compared to WT (Fig. S2A), validating RNAseq data. Hierarchal clustering of the differentially expressed genes in adherens junction signaling also partitioned into two distinct clusters to separate *Mfn1*^*−/−*^ and WT oocytes (Fig. 3e). Differential expression of these genes (*Cdh17* and *Myh2)* was also confirmed by qRT-PCR (Fig. S2B).

### *Mfn1* depletion in oocytes results in impaired oocyte and granulosa cell communication

As we found follicle developmental arrest in *Mfn1*^*−/−*^ mice (Figs. 1 and 2), and RNAseq analysis revealed differential expression of adherens junction signaling genes in *Mfn1*^*−/−*^ oocytes, we next assessed the expression of E-cadherin and N-cadherin in WT and *Mfn1*^*−/−*^ oocytes and ovarian sections, by qPCR and immunofluorescence (IF), respectively. E-cadherin is expressed exclusively in the oocyte and localized to oocyte membrane while N-cadherin is located mainly in granulosa cells and localizes to the interface between granulosa cells, as well as granulosa cells and the oocyte^20^.

**Fig. 4:**
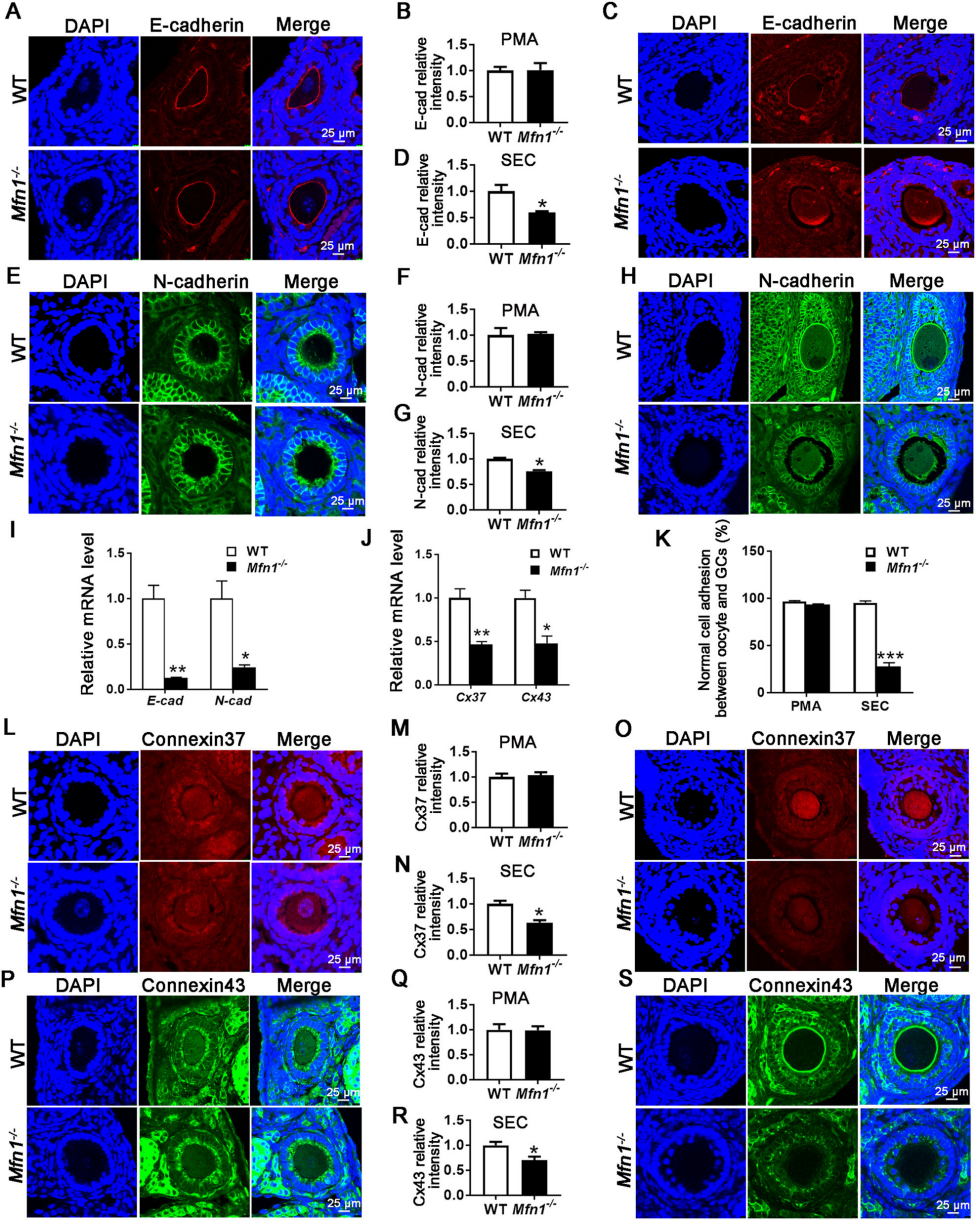
Deletion of *Mfn1* in oocytes inhibits adhesion and gap junction protein expression. **a**, **e** E-cadherin and N-cadherin immunofluorescence in primary follicles of *Mfn1*^*−/−*^ and WT mice ovaries. **c**, **h** E-cadherin and N-cadherin immunofluorescence in secondary follicles from *Mfn1*^*−/−*^ and WT ovaries. **b**, **d**, **f**, **g** Quantitative analysis of E-cadherin and N-cadherin immunofluorescence in primary and secondary follicles from *Mfn1*^*−/−*^ and WT mice ovaries. **i** Secondary follicle-enclosed oocytes and granulosa cells were collected from *Mfn1*^*−/−*^ and WT ovaries. *E-cadherin* and *N-cadherin* mRNA expression was assessed using qRT-PCR in oocytes and granulosa cells, respectively. **j** *Cx37* and *Cx43* mRNA expression assessed using qRT-PCR in oocytes and granulosa cells, respectively. **k** Quantification of normal adhesion between oocytes and granulosa cells in secondary follicles of *Mfn1*^*−/−*^ and WT mice. Abnormal adhesion was described as low expression of E/N cadherins and increased distance between the oocyte and granulosa cells. **l**, **p** Cx37 and Cx43 immunofluorescence in primary follicles of *Mfn1*^*−/−*^ and WT ovaries. **o**, **s** Cx37 and Cx43 immunofluorescence in secondary follicles from *Mfn1*^*−/−*^ and WT mice ovaries. **m**, **n**, **q**, **r** Quantitative analysis of Cx37 and Cx43 immunofluorescence in primary and secondary follicles from *Mfn1*^*−/−*^ and WT mice ovaries. Data presented as mean ± SEM. *p *<* 0.05, **p *<* 0.01 vs. WT from *t*-test. PMA primary follicle, SEC secondary follicle, E-cad E-cadherin, N-cad E-cadherin, Cx37 Connexin37, Cx43 Connexin43

IF results showed proper localization and expression of E-cadherin and N-cadherin in the primary follicles of *Mfn1*^*−/−*^ and WT mice (Fig. 4a, b, e, f). However, levels of E-cadherin (0.59 ± 0.026 vs. 1 ± 0.124, *p* *<* 0.05) and N-cadherin (0.7615 ± 0.019 vs. 1 ± 0.024, *p* *<* 0.01) proteins were significantly decreased in the secondary follicles of *Mfn1*^*−/−*^ mice compared to WT (Fig. 4c, d, g, h). Consistent with these data, *E-cadherin* and *N-cadherin* mRNA levels were also decreased in *Mfn1*^*−/−*^ oocytes and granulosa cells, respectively (Fig. 4i). In addition, in *Mfn1*^*−/−*^ mice, oocytes were detached from the surrounding granulosa cells of arrested secondary follicles. Percentage of normal adhesion between oocyte and granulosa cell was dramatically lower in *Mfn1*^*−/−*^ secondary follicles compared to WT (27.78% ± 4.005 vs. 95.33% ± 2.028, *p* *<* 0.001) (Fig. 4k). Consistent with this finding, numerous denuded oocytes were detected following ovarian puncture during GV stage oocyte collection in *Mfn1*^*−/−*^ mice (Fig. S3). These results suggest that *Mfn1* depletion in oocytes inhibits oocyte granulosa cell interactions in secondary follicles by downregulating the E-cadherin and N-cadherin expression.

### Expression of connexin 37 and connexin 43 is decreased by *Mfn1* deficiency

After establishing aberrant expression of E-cadherin and N-cadherin, we examined whether gap junction formation is also compromised in *Mfn1*^*−/−*^ mice. First, we quantified connexin37 (Cx37) expression since it is the primary connexin produced by the oocyte and is involved in forming gap junctions between oocytes and granulosa cells^21^. IF results showed that protein levels of Cx37 were similar in the primary follicle-enclosed oocytes (Fig. 4l, m), but significantly decreased in secondary follicle-enclosed oocytes of *Mfn1*^*−/−*^ mice compared to WT (0.63 ± 0.048 vs. 1 ± 0.063, *p* *<* 0.05) (Fig. 4o, n). Next, we assessed connexin 43 (Cx43), which is expressed by granulosa cells and is important in formation of gap junctions between granulosa cells^21^. Similarly, while there was no significant difference in Cx43 immunofluorescence in primary follicles (Fig. 4p, q), secondary follicles showed decreased expression in *Mfn1*^*−/−*^ mice compared to WT (0.70 ± 0.068 vs. 1 ± 0.068, *p* *<* 0.05) (Fig. 4s, r). In addition, expression of *Cx37* and *Cx43* at the mRNA level were significantly decreased in the *Mfn1*^*−/−*^ oocytes and granulosa cells, respectively. (Fig. 4j).

### Transzonal processes are impaired in *Mfn1*^*−/−*^ follicle-enclosed oocytes

Transzonal processes (TZPs) anchor the first layer of granulosa cells to the oocyte, enabling the formation of intercellular gap junctions necessary for follicular development^22^. Here, we assessed the structure of TZPs by detecting expression of F-actin. Secondary follicles from *Mfn1*^*−/−*^ and WT mice were fixed and labeled with rhodamine-phalloidin. WT follicles showed compact and organized TZPs distributed through the zona pellucida, however, *Mfn1*^*−/−*^ oocytes displayed very weak and disorganized TZPs (Fig. S4A). Linescan profiles were used to measure and quantify TZP density. We calculated the total fluorescence under the peaks corresponding to the plasma membrane and zona pellucida (Fig. S4B). In *Mfn1*^*−/−*^ oocytes, the total TZP fluorescence was significantly lower compared to WT (1852 ± 159.8 vs. 6807 ± 1004, *p* *<* 0.001) (Fig. S4C). EM analysis also revealed that secondary follicles showed defective contact between oocyte and granulosa cells (Fig. S4D). Furthermore, gap junctions were significantly decreased in the secondary follicles of *Mfn1*^*−/−*^ mice compared to WT (Fig. S4E, black arrow). Together, these findings indicated that oocyte–granulosa cell connections were disrupted in *Mfn1*^*−/−*^ mice.

### *Mfn1*^−*/−*^ follicle-enclosed oocytes show increased expression of pro-apoptotic genes

**Fig. 5:**
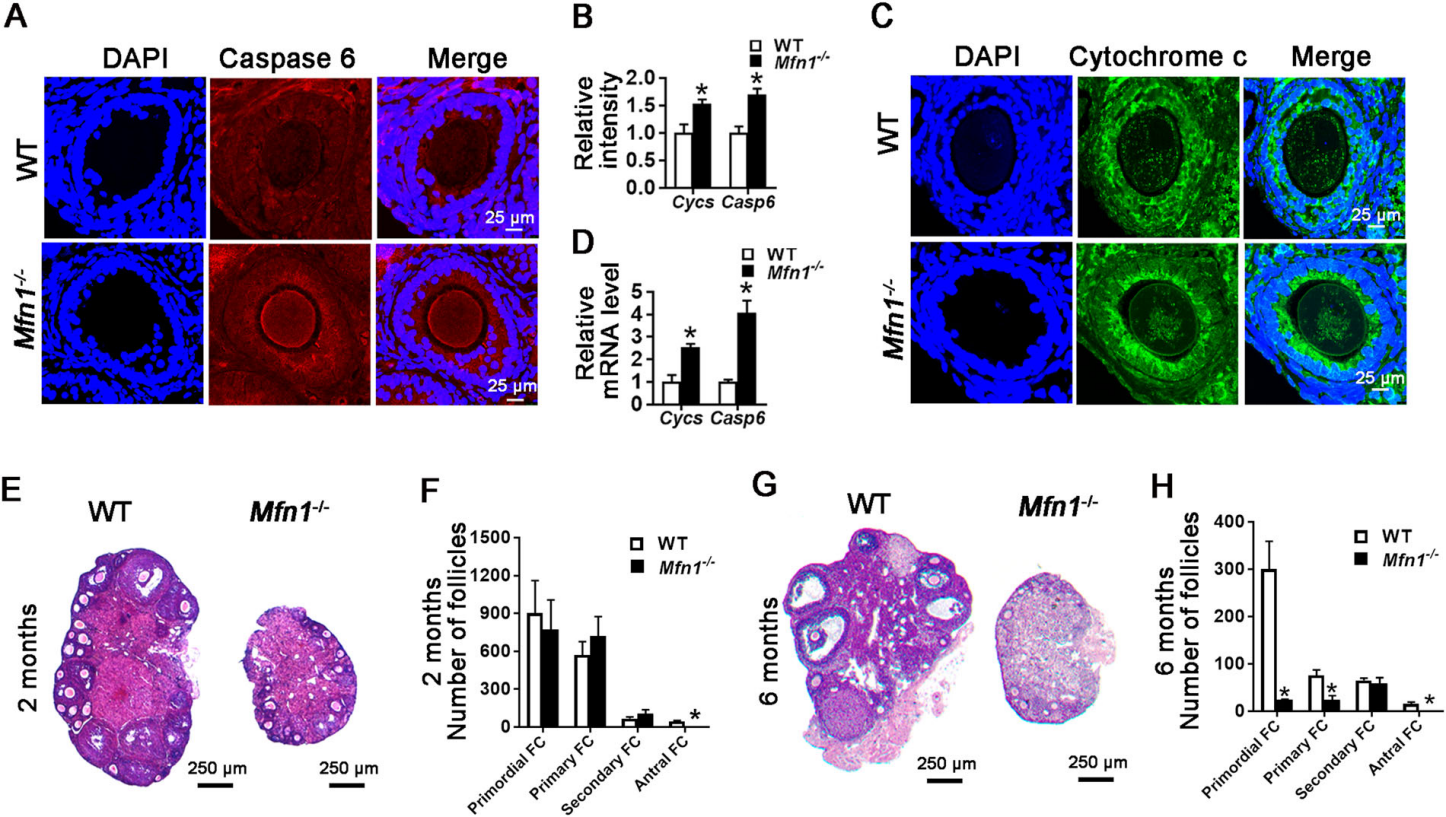
Oocyte apoptosis and follicular depletion in *Mfn1*^*−/−*^ mice. **a**, **c** Caspase 6 and cytochrome c immunofluorescence in secondary-follicle enclosed oocytes from WT and *Mfn1*^*−/−*^ mice ovaries. **b** Quantitative analysis of caspase 6 and cytochrome c immunofluorescence in secondary follicle-enclosed oocytes from WT and *Mfn1*^*−/−*^ mice ovaries. **d** Secondary follicle-enclosed oocytes were collected from *Mfn1*^*−/−*^ and WT ovaries. *Caspase 6* and *cytochrome c* mRNA expression was assessed by qRT-PCR in oocytes from WT and *Mfn1*^*−/−*^ mice. **e**, **g** Follicle development was assessed in ovaries of unstimulated 2 and 6-month-old *Mfn1*^*−/−*^ and WT mice. **f**, **h** Bar charts showing follicle counts from 4 mice for each genotype and time point. Data represent mean ± SEM. *p *<* 0.05, ***p *<* 0.001 vs. WT using *t*-test. *Cycs* cytochrome c, *Casp6* caspase 6

Oocyte apoptosis leads to follicular developmental arrest^23^. As the pathway analysis of RNAseq data identified death receptor signaling genes as being significantly upregulated in *Mfn1*^*−/−*^ oocytes, we next assessed the expression of caspase 6 (CASP6) and cytochrome c (CYCS), two key mediators of this pathway, in *Mfn1*^*−/−*^ and WT mice. IF results showed significantly increased CASP6 expression in *Mfn1*^*−/−*^ oocytes compared to WT (1.7 ± 0.1072 vs. 1 ± 0.1228, *p* < 0.05) (Fig. 5a, b). Similarly, CYCS expression was also elevated in *Mfn1*^*−/−*^ mice oocytes (1.53 ± 0.08 vs. 1 ± 0.15, *p* < 0.05) (Fig. 5c, b). In addition, abnormal mitochondrial aggregation, which is considered to be an upstream event to CYCS release during apoptosis^24^, was observed in *Mfn1*^*−/−*^ oocytes (Fig. 5c). Consistent with these data, *Casp6* and *Cycs* mRNA expressions were greatly increased in *Mfn1*^*−/−*^ oocytes (Fig. 5d).

### Oocyte specific deletion of *Mfn1* results in accelerated depletion of ovarian follicular reserve

During ovarian aging, increased apoptosis is observed in female germ cells^25^. As we found higher expression of pro-apoptotic genes in *Mfn1*^*−/−*^ oocytes, and previous studies have shown accelerated follicle depletion in mice deficient for mitochondrial stress response gene *Clpp*^26^, we next assessed follicle numbers in unstimulated *Mfn1*^−/−^ and WT mice ovaries at different stages of female reproductive lifespan. At 2 months, number of primordial, primary and secondary follicles did not differ between WT and *Mfn1*^−/−^ ovaries, while *Mfn1*^−/−^ ovaries had no antral follicles (Fig. 5e, f). By 6 months, *Mfn1*^−/−^ ovaries had significantly lower number of primordial and primary follicles (Fig. 5g, h). By 9 months, *Mfn1*^−/−^ ovaries showed very low numbers of primordial, primary, and secondary follicles (Fig. S6A and S6B). At 12 months (Fig. S6D and S6E), *Mfn1*^−/−^ ovaries continued to show depletion of follicles in all stages. Serum Anti-Mullerian hormone (AMH) levels were also significantly lower in 2-, 6-, 9-, and 12-months-old *Mfn1*^−/−^ mice compared to WT (Fig. S6C). These results demonstrate that oocyte-specific deletion of *Mfn1* results in accelerated follicular depletion, leading to a phenotype similar to that observed in women with diminished ovarian reserve.

### Ceramide is accumulated in *Mfn1*^*−/−*^ oocytes and ceramide inhibitor myriocin partially rescues folliculogenesis in *Mfn1*^*−/−*^ mice

**Fig. 6:**
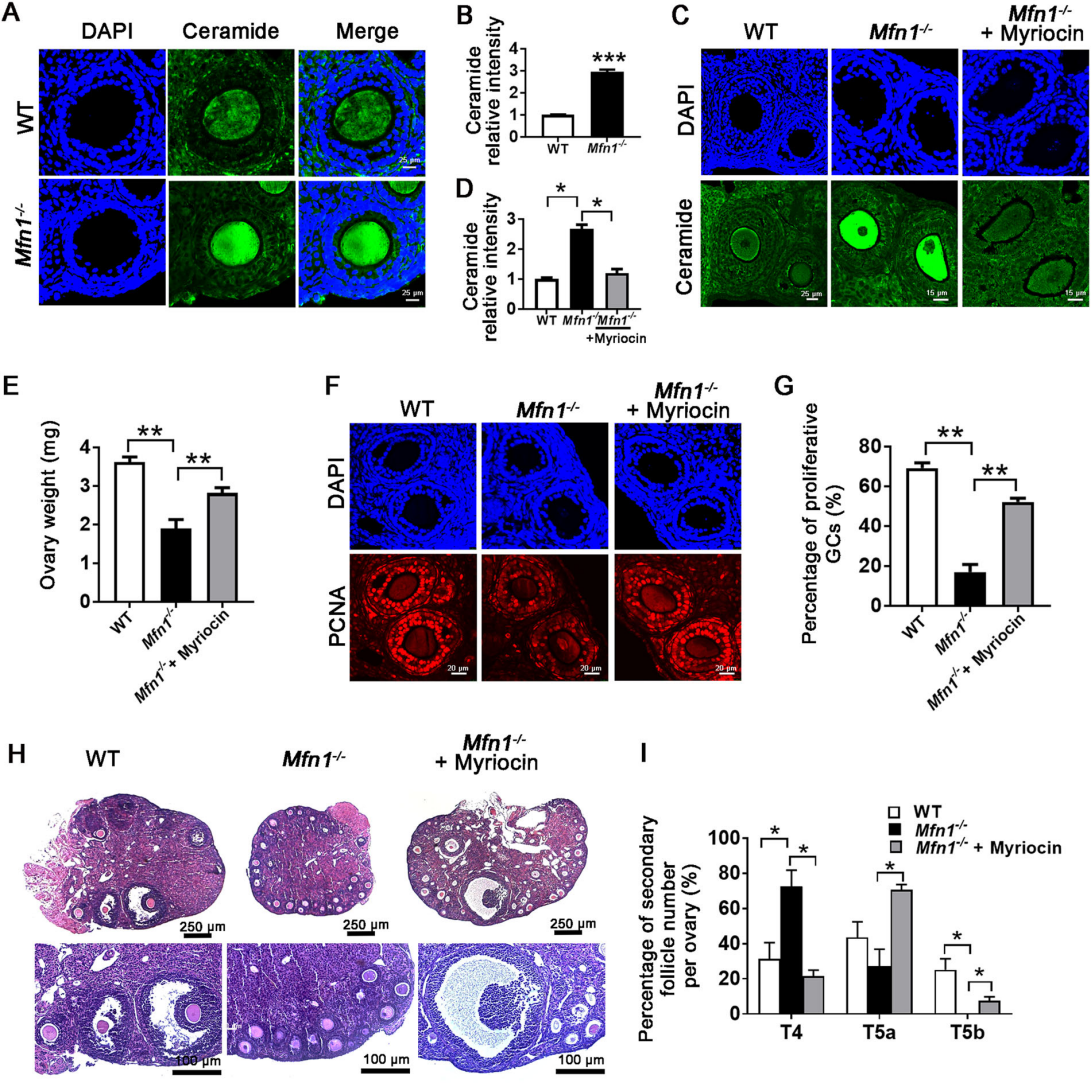
Ceramide accumulation and rescue in *Mfn1*^*−/−*^ mice. **a**, **b** Ceramide immunofluorescence in secondary follicle-enclosed oocytes from *Mfn1*^*−/−*^ and WT ovarian sections. Bar chart shows the quantitative analysis of ceramide intensity in *Mfn1*^*−/−*^ and WT ovarian sections. **c**, **d** *Mfn1*^*−/−*^ and WT mice (8-week-old) were injected with myriocin 1.5 mg/kg (*Mfn1*^*−/−* *+* ^myriocin) once a day for 21 consecutive days, and compared to *Mfn1*^*−/−*^ or WT controls injected with saline. Ovaries were extracted and ceramide immunofluorescence in secondary follicle-enclosed oocytes was assessed. Bar chart shows the quantitative analysis of ceramide intensity. **e** Ovary weight in WT, *Mfn1*^*−/−*^ and myriocin-treated *Mfn1*^*−/−*^ (*Mfn1*^*−/−* *+* ^myriocin) mice. **f**, **g** PCNA immunofluorescence assays and the quantification of PCNA-positive granulosa cells in WT, *Mfn1*^*−/−*^, and *Mfn1*^*−/−* ^*+* myriocin mice ovarian sections. **h**, **i** Representative ovarian section micrographs of WT, *Mfn1*^*−/−*^, and *Mfn1*^*−/−* ^*+* myriocin mice. The percentage of secondary follicle subtypes per ovary was quantified (*n* = 5–8 mice per group). Data presented as mean ± SEM. *p < 0.05 vs. WT using *t*-test. T4: follicles with two layers of cuboidal granulosa cells, T5a: follicles with 3 layers of granulosa cells, T5b: follicles with many layers of granulosa cells but no antral cavity

In addition to showing significant upregulation of death receptor signaling, RNAseq analysis revealed increased ceramide biosynthesis signaling in *Mfn1*^*−/−*^ oocytes. Ceramide has been reported to induce apoptosis by releasing of cytochrome c from mitochondria and by activating effector caspases^24^. By performing IF in ovarian sections, we found ceramide content of *Mfn1*^*−/−*^ oocytes to be significantly higher compared to WT (3.56 ± 0.09 vs. 1 ± 0.03, *p* *<* 0.001) (Fig. 6a, b). In addition, *Cer4* and *Smpd2* (enzymes required for ceramide synthesis) were upregulated in *Mfn1*^*−/−*^ oocytes compared to WT (Fig. S7).

We then tested whether ceramide synthesis inhibitor myriocin could rescue *Mfn1*^*−/−*^ oocyte function and follicle growth. *Mfn1*^*−/−*^ mice were injected with myriocin (1.5 mg/kg) daily for 21 consecutive days before harvesting ovaries, and compared to *Mfn1*^*−/−*^ mice injected with saline, and WT mice injected with saline. IF results showed significantly lower ceramide levels in oocytes of *Mfn1*^*−/−*^ mice treated with myriocin compared with untreated *Mfn1*^*−/−*^ mice (Fig. 6c, d). Ovaries of *Mfn1*^*−/−*^ mice treated with myriocin were heavier (Fig. 6e), and showed a higher number of PCNA-positive granulosa cells in secondary follicles (52.21% ± 1.85 vs. 17.01% ± 3.87, *p* *<* 0.01) (Fig. 6f, g). In addition, ovaries of *Mfn1*^*−/−*^ mice treated with myriocin had an increased number of follicles with more than two layers of granulosa cells (type 5a and 5b) compared to untreated *Mfn1*^*−/−*^ mice ovaries (Fig. 6h, i). Most importantly, *Mfn1*^*−/−*^ mice treated with myriocin developed antral follicles (Fig. 6h). Collectively, our findings indicate that inhibition of ceramide synthesis using myriocin partially rescues impaired follicle development in *Mfn1*^*−/−*^ mice.

## Discussion

Mitochondria play an essential role in generating energy to support oocyte and embryo development^27^. In addition, they have a number of additional functions including regulation of calcium metabolism, signal transduction, and apoptosis^28^, with yet undefined implications for early development. In the current study, we aimed to investigate whether oocyte-specific targeted deletion of *Mfn1*, a key regulator of mitochondrial fusion, affects female fertility. *Mfn1*^−/−^ mice did not produce any pups (Fig. 1a) and did not generate mature oocytes (Fig. 1b). The communication between *Mfn1*^−/−^ oocytes and surrounding granulosa cells through paracrine factors and cell junctions were impaired (Figs. 1k, l, 4) and follicles of *Mfn1*^−/−^ mice arrested at the pre-antral stage (Fig. 1c-j). Collectively, these findings demonstrate that folliculogenesis and oogenesis require MFN1 in oocytes, and that MFN2, the other mammalian mitofusin with approximately 60% sequence homology to MFN1 (and similar N-terminal GTPase domain including canonical G1–G4 motifs), cannot compensate for the absence of MFN1^29^.

During the past two decades, sphingolipids have emerged as essential second messengers in a variety of signal transduction pathways^30^. Most of this research has focused on ceramide, a membrane sphingolipid and an important inducer of programmed cell death^24,25,30^, and cell cycle arrest^31–33^. Previous studies showed that ceramide accumulation in porcine oocytes induced by palmitic acid is associated with mitochondrial dysfunction^34^, and that acid ceramidase (a ceramide metabolizing enzyme), improves the quality of oocytes and embryos and the outcome of IVF^35^. Interestingly, ceramide has been implicated in age-related acceleration of apoptosis in the female germline^25,36^. Based on these data and the RNAseq analyses, which identified ceramide biosynthesis pathway as a key target affected by *Mfn1* deletion, we tested whether elevated ceramide levels contribute to the *Mfn1*^*−/−*^ reproductive phenotype by inducing apoptosis and promoting cell cycle arrest. We found ceramide expression in *Mfn1*^*−/−*^ oocytes to be dramatically increased compared to WT. In addition, treatment of *Mfn1*^*−/−*^ mice with myriocin, which blocks de novo ceramide synthesis by inhibiting the key enzyme serine palmitoyltransferase^37–39^, resulted in improved growth of secondary follicles and formation of antral follicles, partially rescuing the reproductive phenotype. Our findings suggest that drugs targeting down-stream metabolic by products, such as ceramide, can potentially treat impaired oocyte viability in the setting of metabolic dysfunction.

The number of resting follicles available in the ovary constitutes the ovarian reserve, and is the primary determinant of response to the ovarian stimulation in women undergoing infertility treatment^40^. In addition, ovarian reserve is a key determinant of age at menopause. Recently, global germline deletion of *Clpp*, which regulates mtUPR, was found to cause accelerated follicular depletion^26^. In the current study, we observed a depletion of primordial and primary follicles starting at 6 months, and depletion of all follicular stages at 9 and 12 months, consistent with an accelerated reproductive aging phenotype in *Mfn1*^*−/−*^ mice. Mitochondrial dysfunction has been reported to accelerate aging by causing increased ROS levels^41^, accumulation of mtDNA mutations^42^, defective ETC function^43^, altered mitochondrial metabolism^44^ and lower mitochondrial membrane potential^45^. In addition, targeted deletion of PolgA (mtDNA polymerase) causes increased mtDNA mutations and a premature aging phenotype^46^. Our observations constitute a novel paradigm connecting impaired mitochondrial dynamics with accelerated follicular depletion and diminished ovarian reserve.

Depletion of primordial follicles in *Mfn1*^*−/−*^ mice is likely due to accelerated recruitment rather than atresia, as Zp3-mediated targeted deletion occurs only after follicles start growing (at the primary follicle stage)^37–39^. AMH, a hormone produced by the granulosa cells of preantral and small antral follicles, beginning when primordial follicles start developing into primary follicles^47,48^, plays a key role in limiting follicle recruitment. Small antral follicles are likely the primary source of AMH due to the larger numbers of granulosa cells they contain and their more developed microvasculature^48^. As *Mfn1*^*−/−*^ mice have smaller secondary follicles with a lower number of granulosa cells, and do not form antral follicles, it is not surprising that their AMH levels were lower compared to WT as early as 2 months of age (Fig. S6), possibly contributing to accelerated follicle recruitment.

**Fig. 7:**
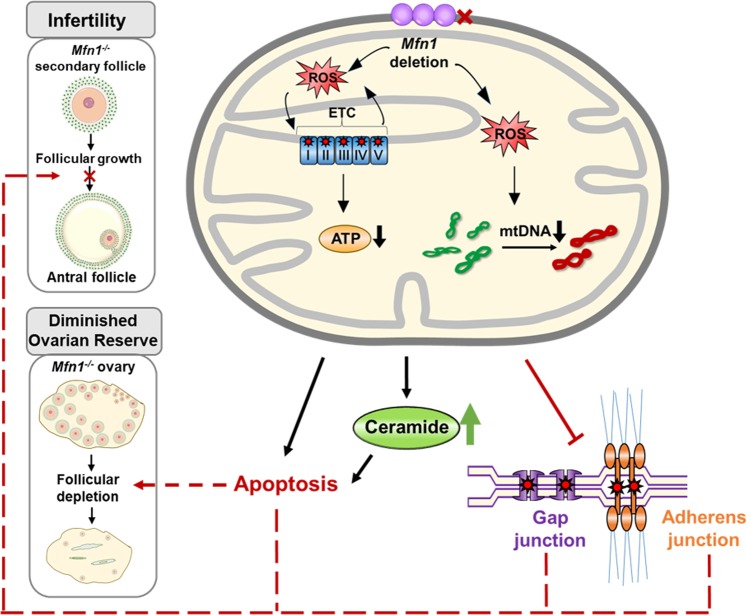
A putative model on the role of MFN1 in female reproductive function. Mitochondrial fusion protein MFN1 is required for female fertility and oocyte and follicle development. In the absence of MFN1, oocytes show mitochondrial dysfunction and accumulation of ceramide, leading to impaired oocyte-granulosa cell communication, increased apoptosis, and accelerated follicular depletion

In this study, we have three important and potentially related findings regarding MFN1′s role on female reproduction. First, we uncovered that oocyte specific deletion of *Mfn1* results in female infertility due to defective oocyte maturation and follicular development. Second, we observed that targeted deletion of *Mfn1* in oocyte results in disruption of adherens and gap junctions between oocytes and granulosa cells and increased apoptosis caused at least in part by accumulation of ceramide. Third, we observed that targeted deletion of *Mfn1* in oocytes results in accelerated follicular depletion, which represents a phenotype reminiscent of diminished ovarian reserve and premature ovarian aging (Fig. 7).

## Methods

### Animals

All animal care and experimental procedures were conducted in accordance with Yale University animal research requirements, using protocols approved by the Institutional Animal Care and Use Committee (protocol # 2017–11207). *Mfn1*^flox/flox^ mice were purchased from The Jackson Laboratory (stock number 026401). Zp3-Cre mice^17,18^ (where Cre is driven by Zp3 promoter) in C57BL/6 background were also obtained from The Jackson Laboratory (stock number 003651). *Mfn1*^flox/flox^ mice were crossbred with *Zp3-Cre* mice to produce mice with oocyte-specific *Mfn1* deletion (*Mfn1*^fl/fl^/*Zp3-Cre* mice), and for simplicity, they are referred to as *Mfn1*^−/−^ mice. Female *Mfn1*^*−/*−^ mice and wild-type (WT) littermates were used in experiments. Genotyping was carried out using the primers shown in Table S1.

### Assessment of fertility

To evaluate the fertility of *Mfn1*^*−/−*^ female mice, seven female mice from each group (*Mfn1*^*−/*−^ or WT, 8-week-old) were mated with adult (12-week-old) WT males of proven fertility for 12 weeks. Two female mice were housed with one adult male mouse, and male mice were rotated weekly. Mating cages were monitored daily, and the number of litters and pups were recorded.

### Histomorphometric analysis of folliculogenesis in ovaries

For hematoxylin and eosin (H&E) staining, ovaries from 8-week-old *Mfn1*^*−/−*^ and WT mice primed (or not primed) with pregnant mare serum gonadotropin (PMSG) 48-h before collection, were fixed in 4% (w/v) paraformaldehyde in Dulbecco’s phosphate buffered saline (DPBS; Sigma) at room temperature overnight, and stored at 4 °C in fresh 70% ethanol until processed. Ovaries were then dehydrated, embedded in paraffin, and 5 μm serial sections were stained with H&E using standard protocol^49^. Every fifth section was assessed, and the total number of follicles for each ovary was determined by counting the follicles containing oocytes with a visible nucleus. Primordial, primary, secondary, and antral follicles were classified as described previously^50^. Briefly, primordial follicles were defined as an oocyte surrounded by a single layer of squamous granulosa cells. Primary follicles possessed an oocyte surrounded by a single layer of cuboidal granulosa cell layer. Secondary follicles consisted of an oocyte surrounded by two or more layers of cuboidal granulosa cells with no visible antrum. Antral follicles contained four or more layers of granulosa cells with a clearly defined single antral space. Secondary follicles were further categorized into type 4 follicles (with two layers of cuboidal granulosa cells), type 5a follicles (with 3 layers of granulosa cells) and type 5b follicles (with many layers of granulosa cells but no follicle fluid), as described by Pedersen and Peters^19^. The diameters of sub-grouped secondary follicles per ovary were measured using ImageJ software (National Institute of Health, NIH, Bethesda, MD, USA).

### Follicle and oocyte collection

Secondary follicles were collected from 8-week-old *Mfn1*^*−/−*^ and WT mice by digesting the ovaries with 1.5 mg/mL collagenase type V (Sigma, St. Louis, MO) for 1 h at 37 °C in M2 medium (Sigma, St. Louis, MO)^5^. Secondary follicle-enclosed oocytes (referred as the oocytes) and granulosa cells were collected by further digesting the harvested secondary follicle with Accutase solution (Sigma, St. Louis, MO) for 15 min. To collect germinal vesicle (GV) stage oocytes, ovaries were obtained from 8-week-old *Mfn1*^*−/−*^ and WT mice^51^ 44–48 h after intraperitoneal injection of 5 IU PMSG (Sigma, St. Louis, MO). Ovaries were then punctured with a 26-gauge needle, and GV stage oocytes were collected in M2 medium (Sigma, St. Louis, MO) and 10 μM milrinone (Sigma, St. Louis, MO) to prevent meiotic resumption. To obtain mature oocytes, an additional injection of 5 IU of human chorionic gonadotrophin (hCG; Sigma, St. Louis, MO) to induce oocyte maturation and ovulation was given 48 h after the PMSG injection. Unfertilized oocytes at metaphase of the second meiotic division (MII) were collected from oviducts 14 h after the hCG injection.

### Electron microscopic analysis

For transmission electron microscopy, 3 *Mfn1*^*−/−*^ and 3 WT female mice were deeply anesthetized 44 h after PMSG injection and perfused with 4% paraformaldehyde/PBS. Both ovaries were removed and fixed overnight at 4 °C with the fixative solution (paraformaldehyde 2%, glutaraldehyde 2.5% in cacodylate buffer 0.1 M, pH 7.4). After ovaries were rinsed in the same buffer twice, they were postfixed in 1% OsO4 in 0.1 M cacodylate buffer at room temperature for 60 min. Specimens were stained en bloc with 2% aqueous uranyl acetate for 30 min, dehydrated in a graded series of ethanol to 100% and embedded in Poly/bed 812 resin. Blocks were polymerized in a 60 °C oven for 24 h. Thin sections (60 nm) were cut by a Leica ultramicrotome and post-stained with 2% uranyl acetate and lead citrate. Cell sections were examined with a FEI Tecnai transmission electron microscope and digital images were recorded with an Olympus Morada CCD camera and iTEM imaging software. Follicle-enclosed oocytes were imaged at ×11500 magnification. Images were taken covering the entire follicle. ImageJ software was used to analyze the mitochondria morphology. Mitochondria area, cytoplasmic area, total mitochondria number, number of cristae, mitochondria length and width were measured. Mitochondrial coverage area was calculated by dividing the total area of mitochondria to the total area of the cytoplasm.

### Quantification of mtDNA copy number in oocytes

To quantify mtDNA levels in oocytes, *Cox3* fragment was amplified using the primers shown in Table S1 and subcloned into pCR™2.1-TOPO®—cloning vector (Invitrogen, Carlsbad, CA) as previously described^16^. One Shot TOP10 Chemically Competent *E. coli* were transformed and grown overnight at 37 °C. Recombinant plasmids were purified using Qiagen plasmid isolation kit and the inserted mtDNA fragment was confirmed by DNA sequence analysis. Plasmid DNA was quantified using NanoDrop 2000 spectrophotometer (Thermo Scientific, Waltham, MA). A standard curve from 10^8^ to 10^1^ plasmid molecules was generated by serial 10-fold dilutions. Single oocytes from *Mfn1*^*−/−*^ and WT mice were individually lysed in 10 μl lysis solution containing 125 μg/ml Proteinase K and 17 μM SDS in sterile water by incubating at 55 °C for 2 h. Then, proteinase K was inactivated by heating the lysis mix at 95 °C for 10 min and the mix was used directly for downstream PCR. Reactions were performed in triplicates. Each 10 μl reaction contained 5 μl of SYBR Green supermix (Bio-Rad Laboratories, Hercules, CA), approximately 0.3 μM of each primer, and 1/3 of oocyte’s total DNA. Each individual oocyte’s mtDNA copy number was extrapolated from the standard curve.

### Determination of ROS levels

6-carboxy-2′,7′-dichlorodihydrofluorescein diacetate (carboxy-H2DCFDA) (Life Technologies, Carlsbad, CA) was used to assess reactive oxygen species (ROS) levels in mouse follicle-enclosed oocytes. This nonfluorescent chemical passes through the plasma membrane and converts to green fluorescent form upon oxidation with ROS and it stays inside the cell for a prolonged period because of its negative charges^52^. We induced ROS generation by exposing follicle enclosed oocytes to 20 mM H_2_O_2_ for 5 min and then incubated these oocytes with 30 µM H2DCFDA in M2 medium for 20 min. Oocytes were washed three times in H2DCFDA-free media and images were captured using Leica SP5 confocal microscope. ImageJ software was used to quantify the fluorescence.

### Quantification of ATP

ATP content of individual single oocytes was determined using the ATP bioluminescent somatic cell assay kit (Sigma, St. Louis, MO). Single oocytes were collected, lysed, and stored individually in 100 μl of somatic cell ATP releasing reagent at −80 °C before use. 100 μl ATP Assay Mix Working Solution was added individually to 96-well plate wells and kept at room temperature for 3–5 min. To a separate vial containing 100 μl of 1× ice-cold somatic cell ATP releasing reagent, 50 μl of samples to be assayed (or standards) were added and swirled briskly; 100 μl of this mix was then transferred individually to the 96-well plate containing 100 μl of ATP Assay Mix Working Solution, and the amount of light emitted was measured immediately with Dynex MLX microliter plate luminometer (Dynex Technologies, Chantilly, VA). Background luminescence was subtracted from all readings. ATP in single oocyte samples was calculated by comparison to a standard curve generated over the range 2.5–500 fmol/100 μl.

### Quantitative reverse-transcription polymerase chain reaction (qRT-PCR)

Total RNA was obtained from follicle-enclosed oocytes and the granulosa cells using RNAqueous Microkit (Thermo Fisher Scientific, Waltham, MA). Reverse transcription was performed using the RETROscript kit (Thermo Fisher Scientific) in two steps: first, template RNA and random primers were incubated at 85 °C for 3 min to eliminate any secondary structures, and then the buffer and enzyme were added and the reaction was carried out at 42 °C for 1 h. qRT-PCR was carried out in an iCycler (Bio-Rad Laboratories, Hercules, CA). cDNA was prepared as described above, and assayed in triplicates. Each experiment was repeated at least three times using individual animals from each genotype. Each 10-µl reaction contained 5 µl of SYBR Green Supermix (Bio-Rad Laboratories), 3 µl of H_2_O, 0.5 µl of each primer, and 1 µl of cDNA. TaqMan Gene expression assays (Life Technologies, Carlsbad, CA) were also used following manufacturer’s instructions. Briefly, each 20-µl reaction contained 1 µl of 20× TaqMan gene expression assay, 10 µl of 2× TaqMan Gene expression master mix, 4 µl of cDNA template, and 5 µl of H_2_O. The 2^−∆∆^CT (cycle threshold) method was used to calculate relative expression levels after normalization to *β-actin* levels. The primers used for real-time PCR reactions were included in Table S1.

### Staining of transzonal processes

Secondary follicles were fixed in 4% formaldehyde (Sigma, St. Louis, MO) for 1 h at 37 °C, washed in phosphate buffered saline (PBS) with 0.1% TritonX-100 and 0.01% polyvinyl alcohol (PVA), and blocked with 3% BSA for 30 min at room temperature. F-actin transzonal processes (TZPs) were labeled using rhodamine-phalloidin (Life Technologies, Carlsbad, CA). After staining, the follicles were washed three times in PBS/PVA and observed with a Leica SP5 spectral scanning confocal microscope. Image J software was used to quantify the data by using the plot profile tool. A line was drawn through the entire follicle to get a line-scan profile. The peaks in the profile corresponding the zona pellucida and plasma membrane were used for quantification of the total fluorescence value.

### Immunofluorescence staining

Paraffin embedded tissue sections were used for staining. For deparaffinization, slides were heated for 45 min at 65 °C prior to rehydration step. In the rehydration step, slides were treated three times with 100% xylene and three times with 100% ethanol. For antigen retrieval, rehydrated slides were incubated with citrate buffer (pH 6.0) in a pressure cooker for 1 h. After cooling down to 27 °C, tissues were permeabilized in 0.5% Triton X-100 for 10 min. This step was followed by a blocking step, where slides were incubated in BSA for 45 min, then washed in 0.5% BSA diluted in PBS (PBB), three times. Then, slides were incubated overnight at 4 °C with primary antibodies for PCNA, E-cadherin, N-cadherin, connexin 37, connexin 43, cytochrome c, caspase 6 (Santa Cruz Biotechnology, Dallas, TX) or ceramide (Enzo Life Sciences, Farmingdale, NY) diluted 1:50. The following day, slides were washed three times with PBB and incubated for 60 min at room temperature with Alexa fluor 594-conjugated or Alexa fluor 488-conjugated secondary antibodies (Thermo Fisher Scientific, Waltham, MA) diluted 1:200. Slides were then washed three times in PBS, incubated with 4′, 6-diamidino-2-phenylindole (DAPI; 1:1000) (Life Technologies, Carlsbad, CA), and washed in PBS. Slides were stored at 4 °C until imaging.

### FSH and AMH testing

To determine serum follicle stimulating hormone (FSH) and Anti-Mullerian hormone (AMH) levels, samples were sent to University of Virginia Center for Research in Reproduction Ligand Assay and Analysis Core, where an in-house RIA (radioimmunoassay) was used to measure FSH, ANSH ELISA kit was used to measure AMH levels.

### RNA sequencing and data analysis

Five secondary follicle enclosed oocytes collected from *Mfn1*^−/−^ or WT mice (*n* = 3) were pooled and cDNA was amplified using Smart-Seq2 protocol as previously described^53,54^. Briefly, poly(A) RNAs were reverse-transcribed directly in whole cell lysate in 0.2% Triton X-100 and 2 U/ul of RNase inhibitors (Invitrogen) in RNase free water. cDNA was then amplified and measured by Qubit 3.0 (Life Technologies, Carlsbad, CA) for concentration and Tapestation 4200 (Agilent Technologies, Santa Clara, CA) for size distribution. RNA sequencing libraries were constructed using Nextera® XT DNA Library Preparation Kit (Illumina, San Diego, CA) and multiplexed using Nextera® XT Index Kit (Illumina). Libraries were quantified by Qubit and Tapestation 4200. Indexed libraries were then pooled and sequenced on Illumina’s Hiseq 2500 platform with 75 bp pair-end reads. In total, we analyzed 6 samples and obtained approximately 212 million reads. The raw FASTQ files and normalized read counts are available at Gene Expression Omnibus (GEO) (www.ncbi.nlm.nih.gov/geo) under the accession number (GSE125699).

Multiplexed sequencing reads that passed filters were trimmed to remove adapters using Cutadapt and low-quality reads were pre-filtered by FASTX-Toolkit before mapping. Clean reads were aligned to the mouse genome (GRCm38/mm10) using STAR with default parameters. Individual mapped reads were quantified to annotation model to calculate gene counts. Differentially expression analysis between *Mfn1*^−/−^ or WT groups was performed using DESeq 2 package. Differentially expressed genes between *Mfn1*^−/−^ and WT groups were determined using false discovery rate (FDR) of *p* value <0.05, foldchange >2 and a minimal of 5 reads as cutoffs. Expression pattern clusters were generated by the K-means clustering algorithm using R. DAVID and Ingenuity Pathway Analysis (IPA) software were used to perform Gene Ontology (GO) and pathways analyses, respectively.

### Myriocin rescue treatment

*Mfn1*^*−/−*^ or WT mice were intraperitoneally injected with Myriocin 1.5 mg/kg (Sigma, St. Louis, MO) or saline once a day for 21 days prior to experiments. Ovaries were then extracted to assess follicular development and junction protein expression through H&E and immunofluorescence staining, respectively.

### Statistical analysis

Quantitative data are expressed as mean ± SEM. Student’s *t*-test was used to analyze the statistical significance between two groups. Data are representative of at least three independent experiments unless otherwise specified. All statistical analyses were done using Graph Pad Prism software version 7 and significance was assessed at *p* < 0.05.

## Supplementary information

Supplemental Figure and Table
