# Supplementary material for: Mitofusin 1 is required for female fertility and to maintain ovarian follicular reserve
Source: Cell Death Dis. 2019 Jul 22;10(8):560. doi: 10.1038/s41419-019-1799-3 (PMC6646343; doi:10.1038/s41419-019-1799-3)
Supplement: Supplementary file 1 — Supplemental Figure and Table [file 41419_2019_1799_MOESM1_ESM.docx]

**Supplemental Information**

**
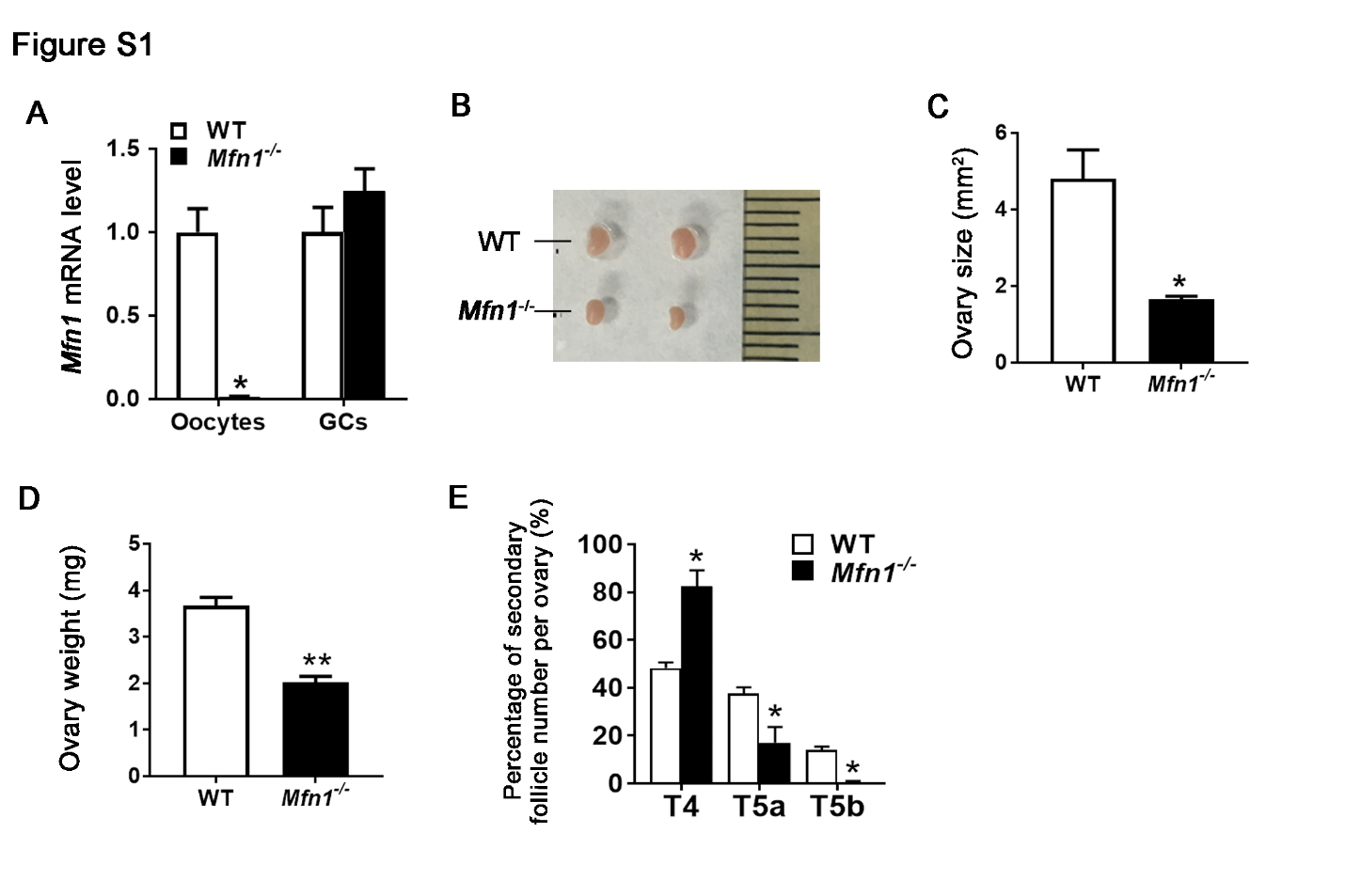
**

**Figure S1. Ovarian size and weight are decreased in *Mfn1^-/-^* mice**

**(A)** *Mfn1* mRNA expression of was assessed using qRT-PCR in secondary follicle-enclosed oocytes and granulosa cells collected from *Mfn1^-/-^* and WT mice. **(B)** Representative photographs of ovaries from 8-week-old *Mfn1^-/-^* and WT mice. **(C)** Ovary size of *Mfn1^-/-^* compared to WT mice. **(D)** Ovary weight of *Mfn1^-/-^* compared to WT mice. **(E)** Relative percentage of secondary follicle subtypes per ovary was quantified as described in Materials and Methods (n=4 mice per genotype). Data presented as mean ± SEM. **p* < 0.05, ***p* < 0.01 vs. WT from *t*-test.

**
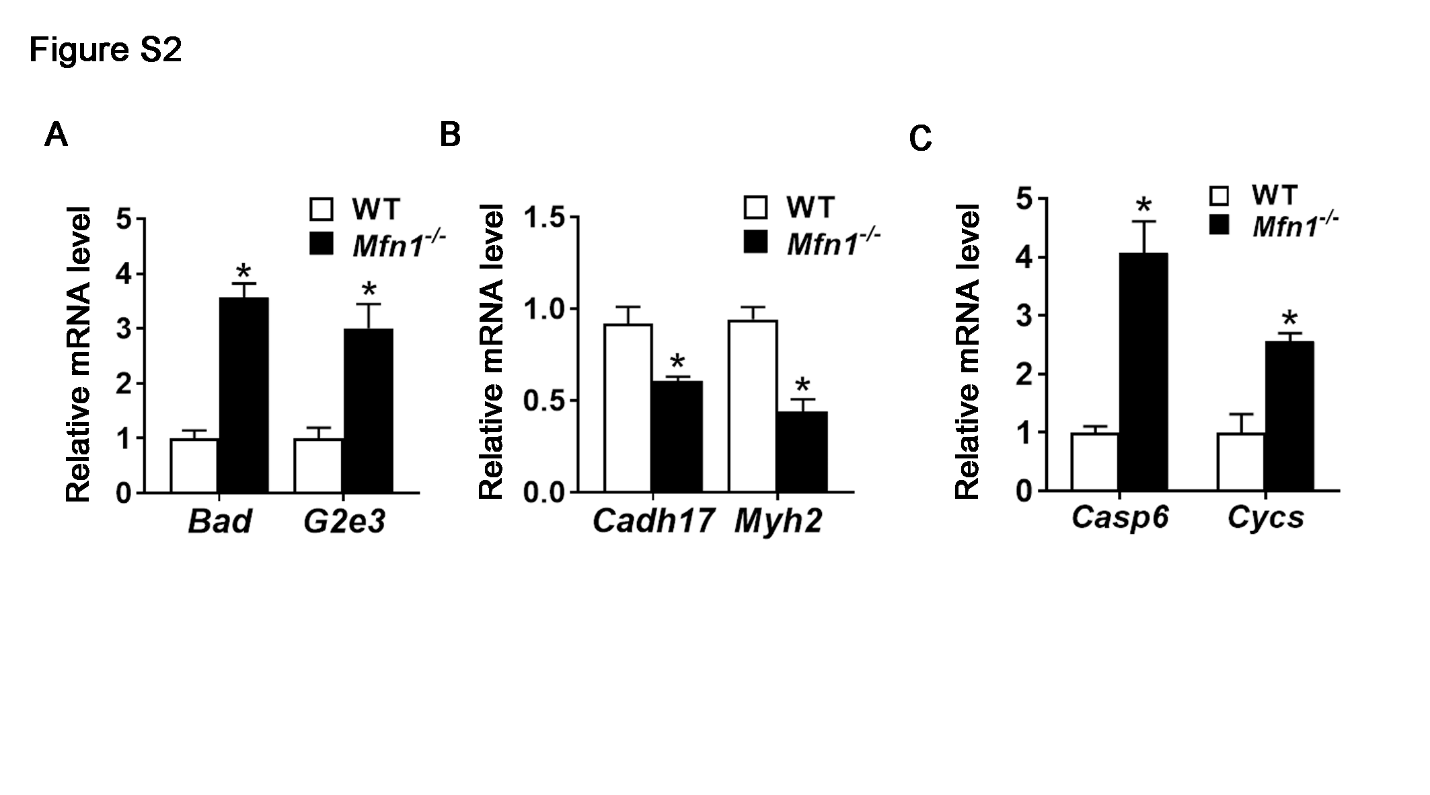
**

**Figure S2. Adherens junction signaling and death receptor signaling pathway genes are differentially expressed in *Mfn1^-/-^* compared to WT mice oocytes.**

**(A, B)** qRT-PCR analysis of *Bad*, *G2e3*, *Cdh17* and *Myh2* in *Mfn1^-/-^* and WT oocytes. Data presented as mean ± SEM. **p* < 0.05, ***p* < 0.01 vs. WT using *t*-test. *Cdh17*: Cadherin 17.

**
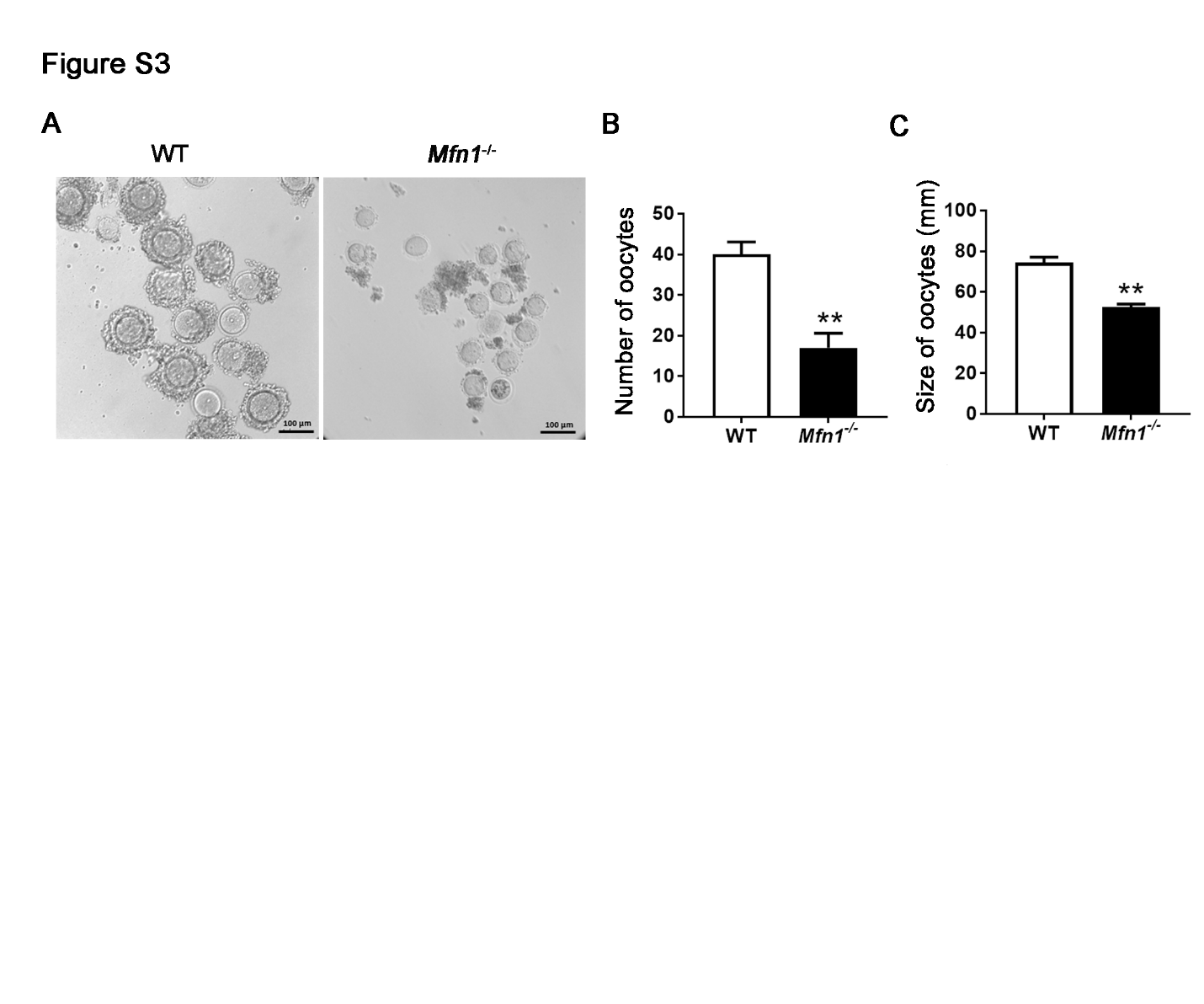
**

**Figure S3. Number and size of oocytes obtained after PMSG-stimulation is decreased in *Mfn1^-/-^* mice**

**(A)** Representative photographs of oocytes collected from PMSG-primed 8-week-old *Mfn1^-/-^* and WT mice. **(B, C)** Number and size of GV oocytes collected from *Mfn1^-/-^* and WT mice. Data presented as mean ± SEM. ***p* < 0.01 vs. WT from *t*-test.

**
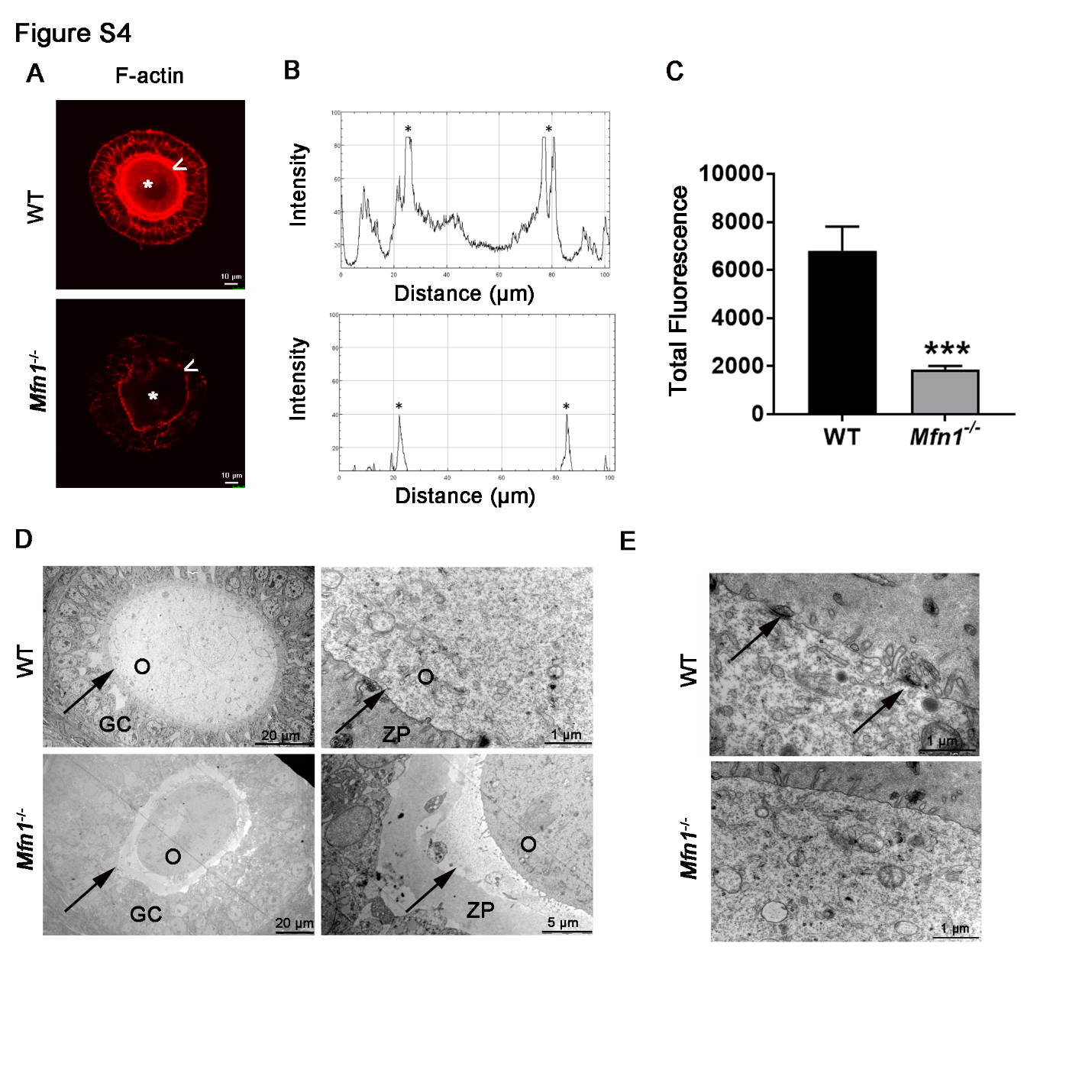
**

**Figure S4. TZP formation is impaired in *Mfn1^-/-^* secondary follicles**

**(A)** Secondary follicles were isolated from the ovaries of *Mfn1^-/-^* and WT mice, fixed, labeled with rhodamine-phalloidin, and examined by confocal microscopy. Asterisk designates oocyte; open arrow designates labeling of TZPs. **(B, C)** Line-scan profiles were used to measure the frequency and distribution of TZPs. The peaks in fluorescence corresponding to the zona pellucida are denoted by an asterisk. The total peak intensity for the zona pellucida and plasma membrane was calculated. Data shown as the mean ± SEM. **p* < 0.05 vs. WT from *t*-test. **(D, E)** Transmission electron microscopic photographs of *Mfn1^-/-^* and WT ovaries. **(D)** Zona pellucida (ZP) between oocyte (O) and granulosa cell (GC) in secondary follicles are indicated by black arrows. **(E)** Gap junctions are indicated by black arrows.

**
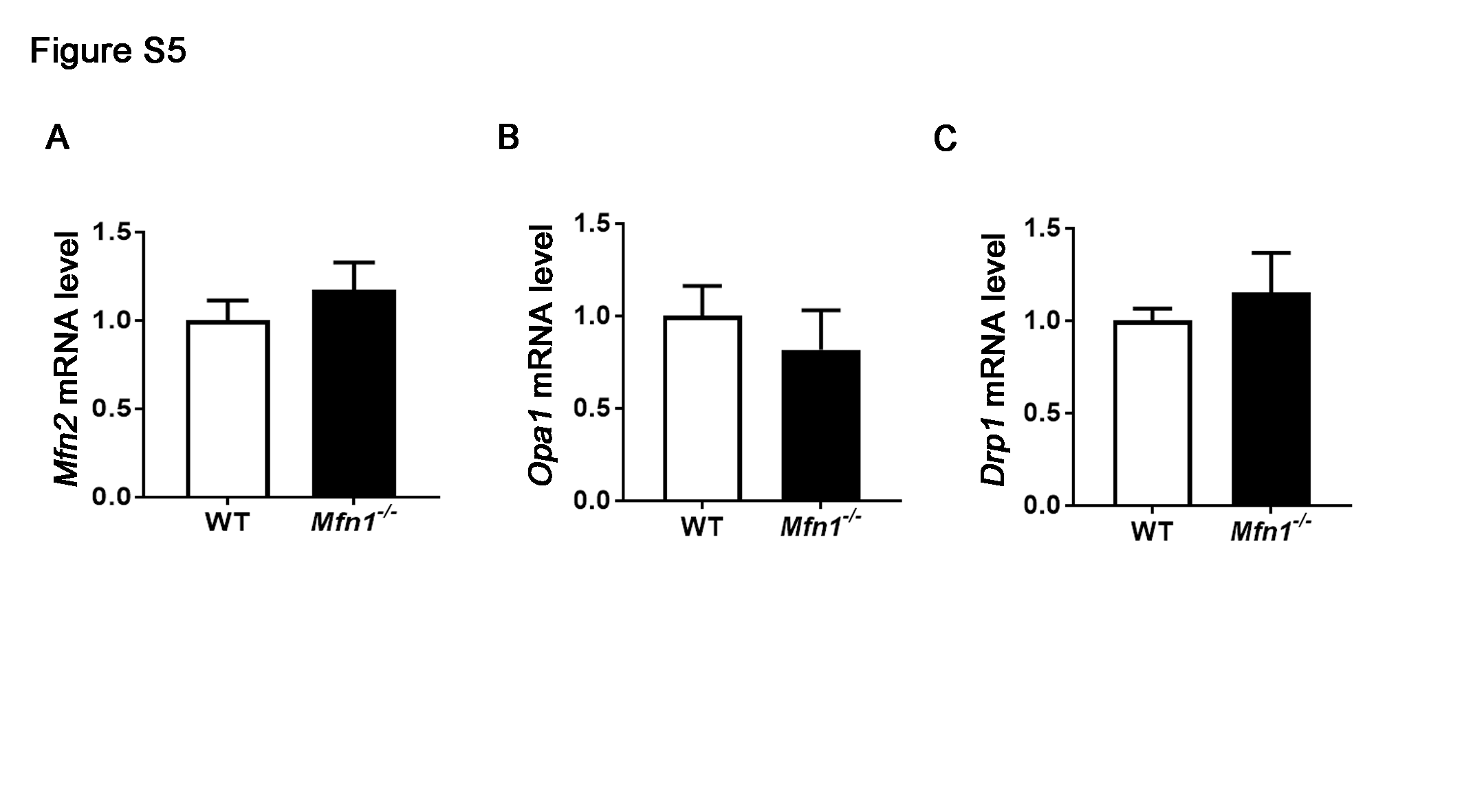
**

**Figure S5. Expression of the mitochondrial dynamics genes *Mfn2*, *Opa1* and *Drp1* are not altered in *Mfn1^-/-^* oocytes**

**(A-C)** *Mfn2, Opa1 and Drp1* mRNA expression was assessed by qRT-PCR in oocytes collected from *Mfn1^-/-^* and WT mice. Data presented as mean ± SEM.


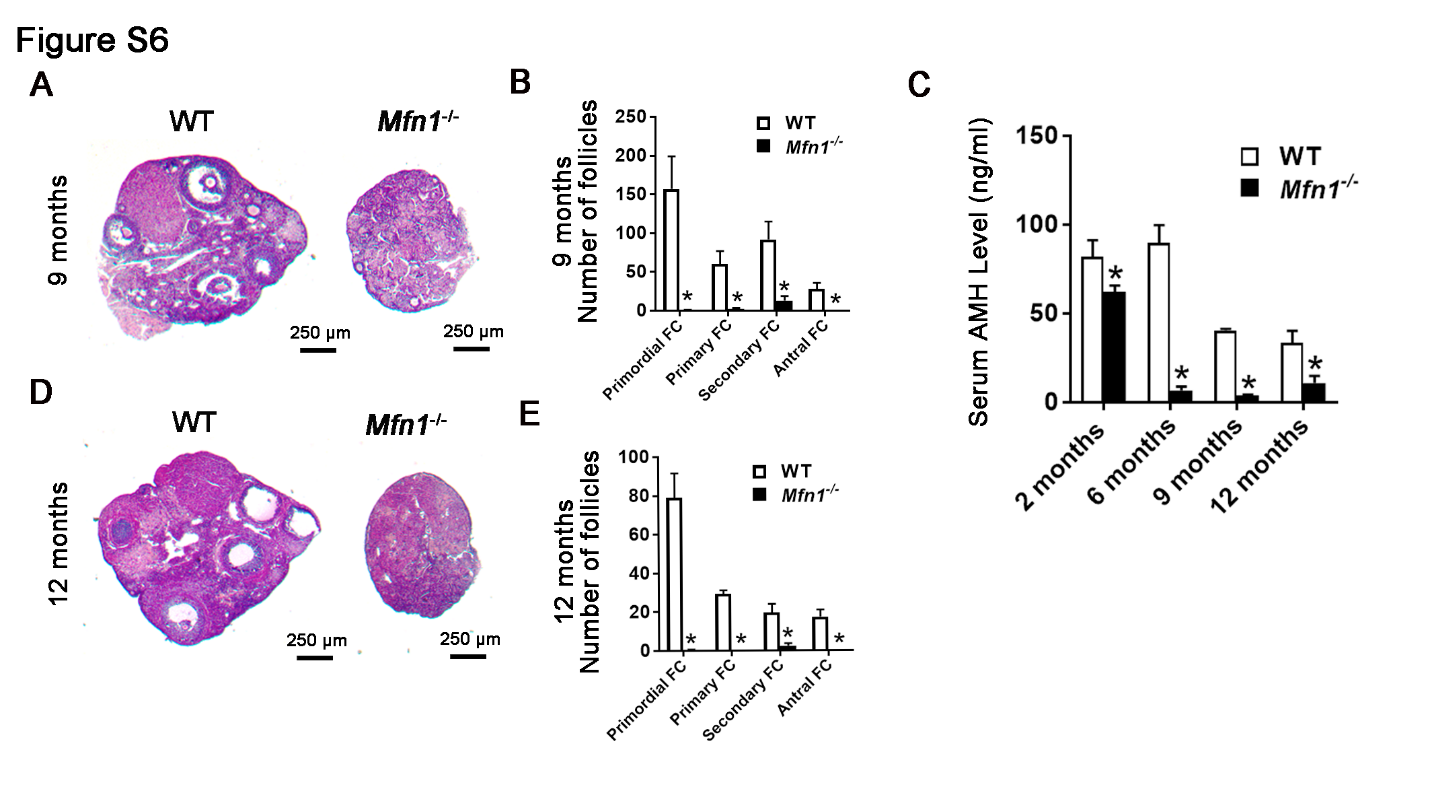


**Figure S6. Serum AMH level and follicular depletion in *Mfn1^-/-^* mice**

**(A, D)** Follicle development was assessed in ovaries of unstimulated 9- and 12-month-old *Mfn1^-/-^* and WT mice. **(B, E)** Bar charts showing follicle counts from 4 mice for each genotype and time point. **(C)** Serum AMH levels in 2-, 6-, 9-, 12-month-old *Mfn1^-/-^* and WT mice (n = 5 per genotype). Data presented as mean ± SEM. **p* < 0.05, vs. WT from *t*-test.

**
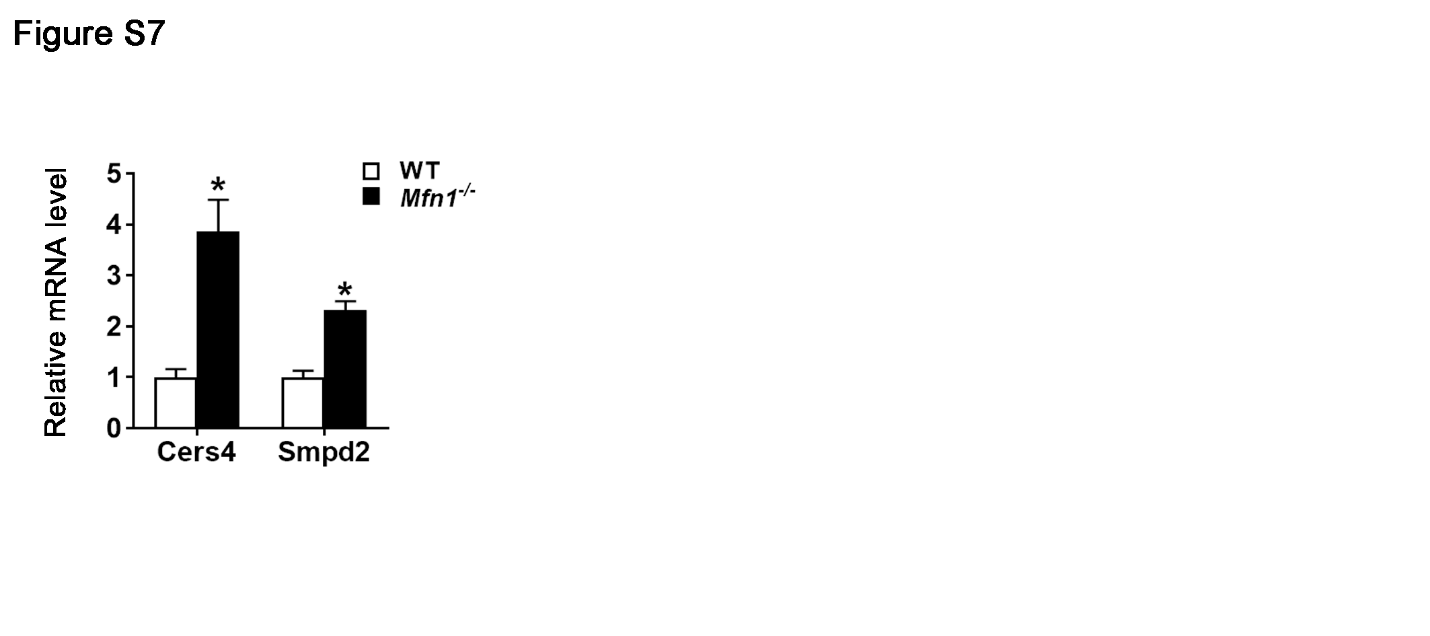
**

**Figure S7. *Cer4 and Smpd2* expressions are decreased in *Mfn1^-/-^* mice oocytes**

**(A)** *Cer4* and *Smpd2* mRNA expression was assessed using qRT-PCR in oocytes collected from *Mfn1^-/-^* and WT mice. Data presented as mean ± SEM. **p* < 0.05, vs. WT from *t*-test.


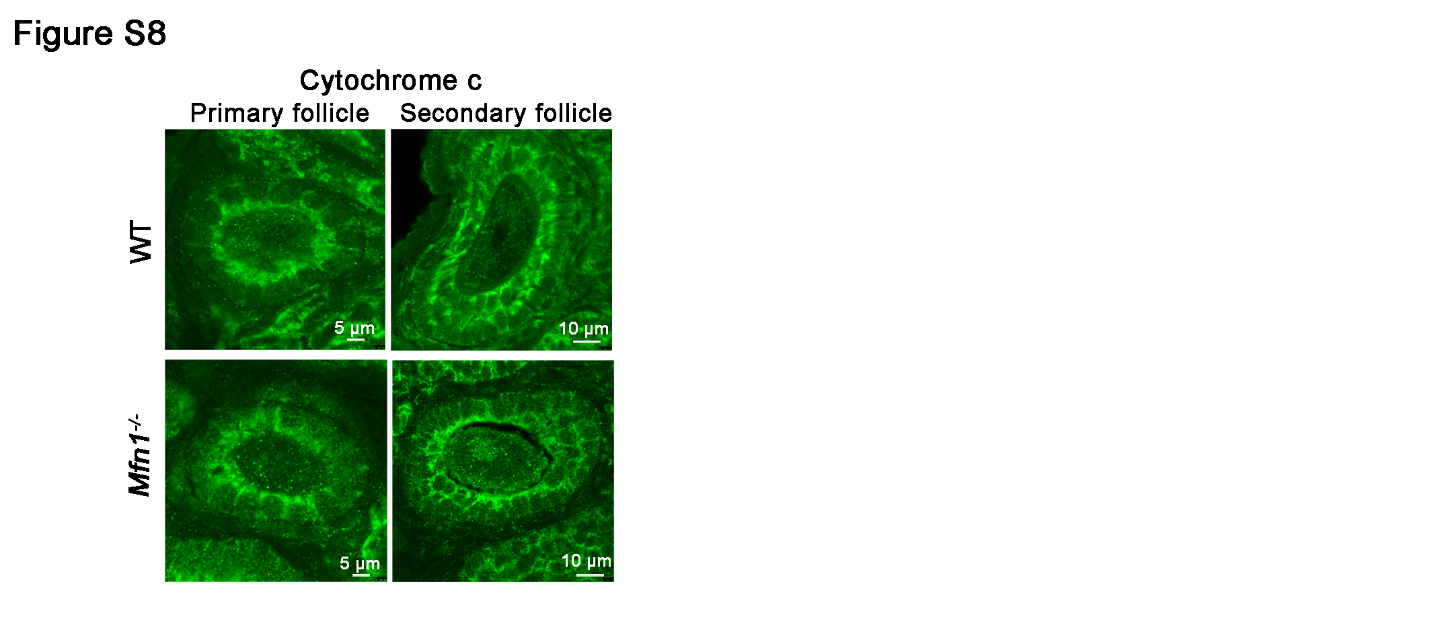


**Figure S8. Mitochondrial aggregation in secondary follicles.**

Cytochrome c immunofluorescence in primary and secondary follicles of *Mfn1^-/-^* and WT mice ovaries.

**Table S1. The list of primers used for quantitative RT-PCR**

| **Gene** | **TaqMan assay number or Primer sequences**  **(5' to 3'; F, forward; R, reverse)** |
| --- | --- |
| *β-actin* | Mm00607939_s1 |
| *β-actin* | F: GGCTGTATTCCCCTCCATCGR: CCAGTTGGTAACAATGCCATGT |
| *Atp5a1* | Mm00431960_m1 |
| *Bmp15* | Mm00437797_m1 |
| *activin-β* | F: AGATCCCGCACCTCGACG: TGGTTGCCTTCATTAGAGACG |
| *Connexin37* | F: ACAGCGGTTGAGTCAGCTTGR: GAGAGATGGGGAAGGACTTGT |
| *Connexin43* | F: TCCCACATCCGATACTGGGTG: CCCGCCGAGACAGGTAGAT |
| *Cox1* | Mm04225243_g1 |
| *Cox3* | F: TTTGCAGGATTCTTCTGAGCR: TGAGCTCATGTAATTGAAACACC |
| *Cre* | F: ATGCTTCTGTCCGTTTGCCR: CAACACCATTTTTTCTGACCC |
| *Drp1* | F: TCCCAATTCCATTATCCTCGCR: CATCAGTACCCGCATCCATG |
| *Dnaja3* | Mm00469723_m1 |
| *E-cadherin* | F: CAGTTCCGAGGTCTACACCTTG: TGAATCGGGAGTCTTCCGAAAA |
| *Follistatin* | F: TGCCTCCTGCTGCTGCTACTR: CACTCTTCCTTGCTCAGTTCTGTC |
| *Gdf9* | Mm00433565_m1 |
| *Hspd1* | Mm00849835_g1 |
| *Hspe1* | Mm00434083_m1 |
| *KIT-ligand* | F: AGTGGATGACCTCGTGTTATGCG: GGATCTAGTTTCTGGCCTCTTCG |
| *Mfn1 flox* | F: TGGTAATCTTTAGCGGTGCTCR: GGAGGACTTTATCCCACAGC |
| *Mfn1* | Mm00612599_m1 |
| *Mfn1* | F: ATGGCAGAAACGGTATCTCCAG: GCCCTCAGTAACAAACTCCAGT |
| *Mfn2* | Mm00500120_m1 |
| *N-cadherin* | F: AGGCTTCTGGTGAAATTGCATG: GTCCACCTTGAAATCTGCTGG |
| *Ndufv1* | Mm00504941_m1 |
| *Opa1* | Mm01349707_g1 |
| *Sdhb* | Mm00458272_m1 |
| *Uqcrc2* | Mm00445961_m1 |

Abbreviations: *Atp5a1*: ATP synthase, H+ transporting, mitochondrial F1 complex, alpha subunit 1; *Bmp15*: bone morphogenetic protein 15; *Cox1*: cytochrome c oxidase subunit I; *Cox3*: cytochrome c oxidase subunit III; *Drp1:* Dynamin related protein 1;*Gapdh*, glyceraldehyde-3-phosphate dehydrogenase; *Gdf9*: growth differentiation factor 9; *Mfn1*: mitofusin 1; *Mfn2*: mitofusin 1; *Ndufv1*: NADH dehydrogenase (ubiquinone) flavoprotein 1; *Opa1*: mitochondrial dynamin like GTPase; *Sdhb*: succinate dehydrogenase complex iron sulfur subunit B; *Uqcrc2*: ubiquinol cytochrome c reductase core protein 2.

**Table S2. Significantly differentially expressed genes between WT *and Mfn1^-/-^* oocytes.**

**Up-regulated genes in MFN1^-/-^ oocytes compared to WT.**

| **Gene ID** | **FDR PValue** | **Ratio** | **Fold change** | **Mean normalized counts (KO)** | **Mean normalized counts (WT)** |
| --- | --- | --- | --- | --- | --- |
| **Krtap4-13** | 8.44E-03 | 2.13E+02 | 212.9852732 | 2.13E+00 | 1.00E-02 |
| **Fgf21** | 2.94E-02 | 1.65E+02 | 165.420289 | 1.86E+01 | 1.13E-01 |
| **Ptgds** | 6.94E-03 | 9.76E+01 | 97.57421883 | 2.27E+01 | 2.33E-01 |
| **BB287469** | 2.18E-02 | 8.47E+01 | 84.73592025 | 8.47E-01 | 1.00E-02 |
| **Btbd6** | 2.04E-03 | 7.97E+01 | 79.69924826 | 7.97E-01 | 1.00E-02 |
| **Krt79** | 4.36E-02 | 5.94E+01 | 59.35492556 | 2.15E+00 | 3.62E-02 |
| **Herpud1** | 1.40E-02 | 5.81E+01 | 58.10856865 | 3.97E+00 | 6.83E-02 |
| **BC080695** | 1.59E-02 | 5.62E+01 | 56.22490007 | 7.98E-01 | 1.42E-02 |
| **Pycard** | 1.43E-02 | 5.55E+01 | 55.52155453 | 1.90E+00 | 3.43E-02 |
| **Usp17lb** | 3.22E-02 | 5.35E+01 | 53.46977942 | 5.24E+00 | 9.80E-02 |
| **Hist1h3c** | 5.25E-03 | 5.11E+01 | 51.14456724 | 5.11E-01 | 1.00E-02 |
| **Gm13040** | 4.53E-02 | 4.89E+01 | 48.88729643 | 2.11E+00 | 4.32E-02 |
| **Hes5** | 1.36E-02 | 4.60E+01 | 45.98326335 | 3.02E+00 | 6.58E-02 |
| **Sp5** | 1.89E-02 | 4.16E+01 | 41.59817093 | 1.51E+00 | 3.63E-02 |
| **Gm11232** | 3.59E-03 | 4.15E+01 | 41.50448201 | 4.15E-01 | 1.00E-02 |
| **Usp17la** | 1.16E-02 | 3.88E+01 | 38.77955685 | 3.88E-01 | 1.00E-02 |
| **B3gnt9** | 2.08E-02 | 3.70E+01 | 36.99818422 | 3.70E-01 | 1.00E-02 |
| **Sohlh1** | 1.53E-02 | 3.51E+01 | 35.10232338 | 3.68E+00 | 1.05E-01 |
| **Mpdu1** | 1.44E-02 | 3.42E+01 | 34.15459989 | 1.43E+00 | 4.19E-02 |
| **Hist1h2bp** | 1.20E-02 | 3.40E+01 | 33.95601435 | 3.40E-01 | 1.00E-02 |
| **Prrg3** | 1.42E-03 | 3.32E+01 | 33.20199655 | 3.32E-01 | 1.00E-02 |
| **Renbp** | 7.65E-03 | 3.29E+01 | 32.92162401 | 5.11E-01 | 1.55E-02 |
| **Gm13119** | 3.19E-02 | 3.24E+01 | 32.37129106 | 1.39E+00 | 4.29E-02 |
| **Pramef25** | 3.41E-02 | 2.86E+01 | 28.60496942 | 3.90E+00 | 1.37E-01 |
| **Usp17le** | 1.66E-02 | 2.78E+01 | 27.75715537 | 7.49E-01 | 2.70E-02 |
| **Kremen2** | 2.45E-02 | 2.77E+01 | 27.65981985 | 1.19E+00 | 4.32E-02 |
| **H2-D1** | 1.96E-02 | 2.69E+01 | 26.93095973 | 8.62E-01 | 3.20E-02 |
| **Zscan4-ps2** | 4.97E-02 | 2.69E+01 | 26.90987551 | 1.71E+00 | 6.37E-02 |
| **Msx1** | 2.04E-03 | 2.66E+01 | 26.60887967 | 2.66E-01 | 1.00E-02 |
| **Zscan4-ps3** | 4.76E-02 | 2.61E+01 | 26.05773367 | 6.92E-01 | 2.66E-02 |
| **Gm6351** | 2.02E-02 | 2.56E+01 | 25.58637468 | 9.81E-01 | 3.84E-02 |
| **Fut1** | 2.17E-02 | 2.54E+01 | 25.42801399 | 2.54E-01 | 1.00E-02 |
| **Nradd** | 1.41E-02 | 2.29E+01 | 22.86681686 | 2.29E-01 | 1.00E-02 |
| **Hist1h3d** | 3.38E-02 | 2.27E+01 | 22.72116422 | 1.55E+00 | 6.80E-02 |
| **4931408C20Rik** | 6.12E-03 | 2.01E+01 | 20.14807245 | 2.93E-01 | 1.45E-02 |
| **Mt1** | 3.26E-02 | 1.96E+01 | 19.565262 | 2.77E+02 | 1.42E+01 |
| **Krt78** | 2.43E-02 | 1.84E+01 | 18.38280604 | 6.98E-01 | 3.80E-02 |
| **Vamp2** | 4.26E-03 | 1.75E+01 | 17.54768479 | 7.64E-01 | 4.35E-02 |
| **Mdk** | 8.06E-03 | 1.75E+01 | 17.4833155 | 1.75E-01 | 1.00E-02 |
| **Tmc8** | 4.26E-03 | 1.74E+01 | 17.44713057 | 5.52E-01 | 3.16E-02 |
| **Hmgn3** | 6.94E-03 | 1.65E+01 | 16.48361403 | 1.04E+00 | 6.31E-02 |
| **Mesp1** | 6.81E-03 | 1.60E+01 | 16.00445394 | 1.60E-01 | 1.00E-02 |
| **Gm17087** | 4.10E-03 | 1.56E+01 | 15.62411531 | 1.56E-01 | 1.00E-02 |
| **Adra2c** | 2.04E-03 | 1.56E+01 | 15.59129177 | 1.56E-01 | 1.00E-02 |
| **Dusp5** | 3.92E-03 | 1.55E+01 | 15.46962254 | 5.24E-01 | 3.39E-02 |
| **Zscan4f** | 1.44E-02 | 1.53E+01 | 15.25625659 | 4.90E+00 | 3.21E-01 |
| **Usp17lc** | 1.33E-02 | 1.52E+01 | 15.20488413 | 2.27E+00 | 1.50E-01 |
| **Hmox1** | 4.93E-02 | 1.51E+01 | 15.05535059 | 1.13E+00 | 7.49E-02 |
| **Spsb2** | 5.59E-03 | 1.44E+01 | 14.37677271 | 9.14E-01 | 6.36E-02 |
| **Foxc2** | 3.76E-02 | 1.31E+01 | 13.13689168 | 3.86E-01 | 2.94E-02 |
| **Gm10330** | 3.30E-03 | 1.31E+01 | 13.11649818 | 1.31E-01 | 1.00E-02 |
| **Zfp872** | 1.51E-02 | 1.30E+01 | 12.97066225 | 2.29E-01 | 1.76E-02 |
| **Tsga8** | 1.04E-02 | 1.29E+01 | 12.91935235 | 6.19E+00 | 4.79E-01 |
| **Amn** | 4.03E-02 | 1.27E+01 | 12.72406169 | 1.92E-01 | 1.51E-02 |
| **B020004J07Rik** | 2.16E-02 | 1.25E+01 | 12.47960674 | 1.21E+00 | 9.73E-02 |
| **Slc24a4** | 9.26E-03 | 1.24E+01 | 12.44186096 | 2.04E-01 | 1.64E-02 |
| **Rab42** | 4.26E-03 | 1.23E+01 | 12.2813316 | 1.23E-01 | 1.00E-02 |
| **Maz** | 2.53E-02 | 1.21E+01 | 12.09309852 | 2.47E-01 | 2.04E-02 |
| **Retn** | 2.83E-02 | 1.18E+01 | 11.84059596 | 3.32E-01 | 2.81E-02 |
| **Mterf2** | 4.26E-03 | 1.17E+01 | 11.70460765 | 1.68E+00 | 1.44E-01 |
| **Gm7682** | 9.26E-03 | 1.16E+01 | 11.62037107 | 3.41E+00 | 2.94E-01 |
| **Zscan4c** | 1.15E-02 | 1.14E+01 | 11.35020728 | 1.38E+01 | 1.22E+00 |
| **Klf2** | 8.44E-03 | 1.13E+01 | 11.28004808 | 4.49E+00 | 3.98E-01 |
| **Cd68** | 1.21E-02 | 1.13E+01 | 11.27487802 | 7.58E+00 | 6.72E-01 |
| **Gm6502** | 2.99E-02 | 1.10E+01 | 10.97792008 | 2.47E+00 | 2.25E-01 |
| **Emc9** | 3.13E-02 | 1.08E+01 | 10.80954623 | 2.54E-01 | 2.35E-02 |
| **Il19** | 1.43E-02 | 1.05E+01 | 10.54754104 | 2.58E-01 | 2.45E-02 |
| **Tmem121** | 2.10E-02 | 1.05E+01 | 10.53145222 | 1.05E-01 | 1.00E-02 |
| **Irx2** | 3.30E-03 | 1.04E+01 | 10.38174207 | 1.04E-01 | 1.00E-02 |
| **Hoxc13** | 1.75E-02 | 1.02E+01 | 10.23276834 | 1.38E-01 | 1.35E-02 |
| **Hist1h1c** | 6.71E-03 | 1.02E+01 | 10.211286 | 1.10E+01 | 1.07E+00 |
| **Dnd1** | 1.61E-02 | 1.00E+01 | 10.0349644 | 9.26E-01 | 9.23E-02 |
| **Cldn7** | 1.53E-02 | 9.76E+00 | 9.757566054 | 2.87E+00 | 2.94E-01 |
| **Gnpda1** | 2.44E-02 | 9.75E+00 | 9.749931745 | 1.94E+00 | 1.99E-01 |
| **Sprr1a** | 4.26E-03 | 9.55E+00 | 9.548784707 | 9.55E-02 | 1.00E-02 |
| **Gstt3** | 2.08E-02 | 9.16E+00 | 9.161230582 | 3.68E+00 | 4.01E-01 |
| **Tpbgl** | 1.33E-02 | 8.96E+00 | 8.957600993 | 8.96E-02 | 1.00E-02 |
| **Gdf1** | 1.57E-02 | 8.57E+00 | 8.569021672 | 8.57E-01 | 1.00E-01 |
| **Arhgdig** | 1.38E-02 | 8.46E+00 | 8.460782211 | 1.04E-01 | 1.23E-02 |
| **Slc25a45** | 2.10E-02 | 8.34E+00 | 8.341820524 | 2.43E-01 | 2.91E-02 |
| **Hes1** | 2.53E-02 | 8.30E+00 | 8.30092397 | 8.46E-01 | 1.02E-01 |
| **Ovca2** | 3.98E-02 | 8.16E+00 | 8.159800138 | 1.16E+00 | 1.42E-01 |
| **Trap1a** | 2.91E-02 | 8.13E+00 | 8.125323099 | 2.78E-01 | 3.43E-02 |
| **Prr15** | 3.84E-02 | 8.12E+00 | 8.120524005 | 3.69E-01 | 4.54E-02 |
| **Sv2a** | 1.27E-02 | 8.06E+00 | 8.0645078 | 1.52E-01 | 1.89E-02 |
| **Gm5662** | 1.33E-02 | 8.04E+00 | 8.042580662 | 8.04E-02 | 1.00E-02 |
| **Smim10l2a** | 3.93E-02 | 8.00E+00 | 7.997891712 | 2.08E-01 | 2.59E-02 |
| **Tcf15** | 2.76E-02 | 7.93E+00 | 7.929153548 | 1.42E+00 | 1.79E-01 |
| **Maged1** | 4.88E-03 | 7.91E+00 | 7.907231904 | 5.00E+00 | 6.33E-01 |
| **Pdf** | 2.34E-02 | 7.65E+00 | 7.650042048 | 1.35E-01 | 1.76E-02 |
| **Ifngr1** | 8.49E-03 | 7.60E+00 | 7.599855517 | 9.61E-02 | 1.26E-02 |
| **Glb1l3** | 1.92E-02 | 7.36E+00 | 7.360645526 | 1.54E-01 | 2.09E-02 |
| **Lrrc3** | 3.13E-02 | 7.35E+00 | 7.346757969 | 7.35E-02 | 1.00E-02 |
| **Teddm1b** | 3.29E-03 | 7.30E+00 | 7.296270013 | 1.40E+01 | 1.92E+00 |
| **Slc35e3** | 4.22E-02 | 7.14E+00 | 7.141573119 | 1.89E-01 | 2.64E-02 |
| **Olfr1477** | 1.92E-02 | 7.13E+00 | 7.125226497 | 1.04E-01 | 1.45E-02 |
| **Ilvbl** | 4.98E-02 | 7.10E+00 | 7.104402331 | 2.41E-01 | 3.39E-02 |
| **Rpp25** | 4.96E-02 | 7.05E+00 | 7.04818227 | 7.17E-01 | 1.02E-01 |
| **Nptxr** | 1.73E-02 | 7.02E+00 | 7.01660577 | 1.19E-01 | 1.69E-02 |
| **Glyctk** | 1.84E-02 | 7.00E+00 | 7.000108876 | 7.00E-02 | 1.00E-02 |
| **Acaa1a** | 6.30E-03 | 6.89E+00 | 6.887640057 | 2.50E+00 | 3.62E-01 |
| **Krt8** | 2.04E-02 | 6.86E+00 | 6.8605176 | 1.68E+00 | 2.45E-01 |
| **Slfn8** | 1.89E-02 | 6.75E+00 | 6.750331257 | 3.77E-01 | 5.59E-02 |
| **Tulp2** | 1.41E-02 | 6.70E+00 | 6.699371981 | 2.45E-01 | 3.66E-02 |
| **Gpr143** | 1.15E-02 | 6.68E+00 | 6.679366478 | 1.32E+00 | 1.98E-01 |
| **Rassf7** | 3.72E-02 | 6.48E+00 | 6.481563255 | 1.77E+00 | 2.73E-01 |
| **Zscan22** | 2.76E-02 | 6.45E+00 | 6.445964759 | 1.26E-01 | 1.95E-02 |
| **Alox8** | 4.67E-02 | 6.38E+00 | 6.381623751 | 1.01E-01 | 1.59E-02 |
| **Hs3st6** | 2.51E-02 | 6.31E+00 | 6.308110053 | 9.32E-02 | 1.48E-02 |
| **Ppp1r1a** | 1.36E-02 | 6.30E+00 | 6.298555178 | 8.39E-02 | 1.33E-02 |
| **Phf11a** | 4.76E-02 | 6.30E+00 | 6.295729446 | 1.12E-01 | 1.78E-02 |
| **Tcirg1** | 3.17E-02 | 6.23E+00 | 6.232192451 | 1.82E-01 | 2.92E-02 |
| **Mtfp1** | 1.76E-02 | 6.23E+00 | 6.228574154 | 4.74E-01 | 7.61E-02 |
| **4930579G24Rik** | 2.82E-02 | 6.21E+00 | 6.205836456 | 4.11E-01 | 6.62E-02 |
| **Tmc6** | 9.89E-03 | 6.17E+00 | 6.174820117 | 7.15E-02 | 1.16E-02 |
| **Zscan4d** | 5.25E-03 | 6.12E+00 | 6.122519748 | 1.74E+01 | 2.84E+00 |
| **Slc27a3** | 4.94E-02 | 6.12E+00 | 6.121094293 | 1.63E-01 | 2.66E-02 |
| **Dkkl1** | 3.67E-02 | 6.10E+00 | 6.102974879 | 6.10E-02 | 1.00E-02 |
| **Isx** | 2.03E-02 | 6.03E+00 | 6.027152419 | 1.60E-01 | 2.65E-02 |
| **Tspan17** | 9.89E-03 | 6.00E+00 | 6.004542175 | 1.40E+00 | 2.33E-01 |
| **Chpf2** | 1.79E-02 | 5.77E+00 | 5.765745851 | 5.77E-02 | 1.00E-02 |
| **Prob1** | 3.25E-02 | 5.73E+00 | 5.733508465 | 9.02E-02 | 1.57E-02 |
| **Eif4ebp1** | 2.92E-02 | 5.65E+00 | 5.648696284 | 2.92E+00 | 5.16E-01 |
| **Spats1** | 1.65E-02 | 5.63E+00 | 5.634818871 | 6.22E-02 | 1.10E-02 |
| **Trip6** | 1.11E-02 | 5.63E+00 | 5.633705418 | 9.94E-01 | 1.76E-01 |
| **N4bp2l1** | 1.09E-02 | 5.63E+00 | 5.62821853 | 4.57E-01 | 8.12E-02 |
| **Dnajb3** | 2.31E-02 | 5.54E+00 | 5.54090881 | 1.40E+00 | 2.53E-01 |
| **Themis2** | 4.03E-02 | 5.49E+00 | 5.493458778 | 6.52E-02 | 1.19E-02 |
| **Mta2** | 1.76E-02 | 5.49E+00 | 5.485427004 | 4.48E-01 | 8.17E-02 |
| **Gm5039** | 3.62E-02 | 5.45E+00 | 5.453937961 | 3.50E+00 | 6.41E-01 |
| **Sord** | 1.23E-02 | 5.44E+00 | 5.443606584 | 4.89E-01 | 8.99E-02 |
| **Dll1** | 2.08E-02 | 5.43E+00 | 5.431960942 | 1.16E-01 | 2.13E-02 |
| **Ftl1** | 4.26E-03 | 5.41E+00 | 5.411679279 | 3.38E+02 | 6.25E+01 |
| **Spdef** | 2.30E-02 | 5.40E+00 | 5.400245376 | 9.46E-02 | 1.75E-02 |
| **Fam89a** | 2.17E-02 | 5.40E+00 | 5.398291848 | 4.36E-01 | 8.07E-02 |
| **Bhlhb9** | 1.23E-02 | 5.39E+00 | 5.390324566 | 8.10E-02 | 1.50E-02 |
| **Shisa4** | 2.36E-02 | 5.34E+00 | 5.344674459 | 1.23E+00 | 2.30E-01 |
| **Sec61a2** | 7.09E-03 | 5.23E+00 | 5.226741197 | 5.89E-01 | 1.13E-01 |
| **H2-Eb1** | 1.51E-02 | 5.22E+00 | 5.22224913 | 2.13E-01 | 4.07E-02 |
| **Pex10** | 3.74E-02 | 5.22E+00 | 5.220114286 | 4.03E-01 | 7.72E-02 |
| **Asah1** | 7.40E-03 | 5.20E+00 | 5.203476428 | 5.44E-01 | 1.05E-01 |
| **Aldh3b1** | 3.52E-02 | 5.14E+00 | 5.142601583 | 3.57E-01 | 6.95E-02 |
| **Paxx** | 1.76E-02 | 5.13E+00 | 5.132947638 | 1.26E+00 | 2.46E-01 |
| **Enho** | 3.09E-02 | 5.13E+00 | 5.129597292 | 3.13E+00 | 6.10E-01 |
| **Slc37a2** | 2.92E-02 | 5.11E+00 | 5.105432174 | 1.02E-01 | 1.99E-02 |
| **Vmn2r94** | 4.95E-02 | 5.09E+00 | 5.08785759 | 5.09E-02 | 1.00E-02 |
| **Wnt5a** | 1.61E-02 | 5.07E+00 | 5.068412825 | 1.55E-01 | 3.05E-02 |
| **Ftl1-ps1** | 4.26E-03 | 5.04E+00 | 5.038170838 | 9.17E+01 | 1.82E+01 |
| **Aldh3b2** | 2.50E-02 | 5.03E+00 | 5.032491693 | 1.08E-01 | 2.14E-02 |
| **Sigirr** | 1.60E-02 | 5.02E+00 | 5.022753209 | 6.39E-02 | 1.27E-02 |
| **Nfic** | 1.51E-02 | 5.02E+00 | 5.022143478 | 1.05E-01 | 2.09E-02 |
| **Prcd** | 4.46E-02 | 5.00E+00 | 5.002368883 | 8.41E-02 | 1.68E-02 |
| **Kdelr3** | 4.14E-02 | 4.97E+00 | 4.968807475 | 1.38E-01 | 2.78E-02 |
| **Slc16a4** | 2.98E-02 | 4.97E+00 | 4.968163462 | 1.83E-01 | 3.69E-02 |
| **Rnh1** | 3.95E-03 | 4.93E+00 | 4.931273351 | 1.31E+00 | 2.65E-01 |
| **Fezf1** | 5.98E-03 | 4.92E+00 | 4.917750984 | 4.92E-02 | 1.00E-02 |
| **Nat8l** | 2.03E-02 | 4.90E+00 | 4.895985726 | 1.26E-01 | 2.57E-02 |
| **Jade1** | 9.70E-03 | 4.85E+00 | 4.852244802 | 6.25E-02 | 1.29E-02 |
| **Asic3** | 1.01E-02 | 4.83E+00 | 4.831981373 | 4.83E-02 | 1.00E-02 |
| **Slc4a11** | 1.95E-02 | 4.83E+00 | 4.831776964 | 5.84E-02 | 1.21E-02 |
| **Nlrc3** | 9.70E-03 | 4.80E+00 | 4.799608382 | 4.47E-01 | 9.31E-02 |
| **Olfr1371** | 4.07E-02 | 4.80E+00 | 4.798159374 | 1.13E+00 | 2.36E-01 |
| **Tmem86a** | 9.65E-03 | 4.76E+00 | 4.762355041 | 1.87E+00 | 3.92E-01 |
| **Gm20431** | 4.97E-02 | 4.75E+00 | 4.751224012 | 1.89E-01 | 3.97E-02 |
| **Angptl8** | 2.85E-02 | 4.71E+00 | 4.70906197 | 7.91E-01 | 1.68E-01 |
| **Fes** | 4.39E-02 | 4.70E+00 | 4.698379138 | 4.70E-02 | 1.00E-02 |
| **Fndc10** | 1.44E-02 | 4.68E+00 | 4.679221315 | 1.94E+00 | 4.15E-01 |
| **Creb3l1** | 1.01E-02 | 4.67E+00 | 4.674712722 | 5.83E-02 | 1.25E-02 |
| **Irx1** | 4.07E-02 | 4.66E+00 | 4.655310227 | 6.91E-02 | 1.48E-02 |
| **Xbp1** | 4.88E-03 | 4.61E+00 | 4.61095663 | 4.60E+00 | 9.98E-01 |
| **Gm7971** | 5.32E-03 | 4.59E+00 | 4.58980253 | 4.59E-02 | 1.00E-02 |
| **Cldn6** | 1.33E-02 | 4.59E+00 | 4.589686719 | 4.39E-01 | 9.57E-02 |
| **Tbc1d17** | 2.73E-02 | 4.56E+00 | 4.561682877 | 3.34E-01 | 7.32E-02 |
| **Hspb1** | 9.70E-03 | 4.56E+00 | 4.561247493 | 1.79E+01 | 3.93E+00 |
| **Ndufa4l2** | 3.60E-02 | 4.55E+00 | 4.548644875 | 4.76E+00 | 1.05E+00 |
| **Asns** | 4.54E-02 | 4.51E+00 | 4.514446813 | 8.80E-01 | 1.95E-01 |
| **4930550L24Rik** | 3.28E-02 | 4.50E+00 | 4.502280759 | 5.74E-01 | 1.27E-01 |
| **Tmed1** | 2.31E-02 | 4.50E+00 | 4.496875438 | 3.25E+00 | 7.22E-01 |
| **Gal3st3** | 1.61E-02 | 4.49E+00 | 4.486865026 | 4.96E-01 | 1.11E-01 |
| **2510039O18Rik** | 2.17E-02 | 4.46E+00 | 4.464595772 | 9.65E-01 | 2.16E-01 |
| **Arl2bp** | 2.92E-02 | 4.46E+00 | 4.46398884 | 2.49E+00 | 5.58E-01 |
| **Rhog** | 2.24E-02 | 4.45E+00 | 4.449772545 | 7.87E-02 | 1.77E-02 |
| **Selenos** | 4.84E-03 | 4.43E+00 | 4.429272706 | 6.29E+00 | 1.42E+00 |
| **Retreg2** | 2.10E-02 | 4.41E+00 | 4.409763693 | 3.25E+00 | 7.36E-01 |
| **Zfp771** | 2.04E-02 | 4.39E+00 | 4.385116576 | 1.97E+00 | 4.49E-01 |
| **Unc93b1** | 4.20E-02 | 4.30E+00 | 4.304645751 | 1.22E-01 | 2.82E-02 |
| **Tcf24** | 3.93E-02 | 4.28E+00 | 4.277618066 | 8.51E-01 | 1.99E-01 |
| **Lgals1** | 2.62E-02 | 4.27E+00 | 4.272361389 | 1.07E+00 | 2.51E-01 |
| **Nup62cl** | 2.05E-02 | 4.25E+00 | 4.252436304 | 1.01E-01 | 2.38E-02 |
| **Dmrtc1c1** | 3.59E-03 | 4.25E+00 | 4.25016907 | 4.25E-02 | 1.00E-02 |
| **Gpha2** | 1.61E-02 | 4.25E+00 | 4.248539156 | 3.15E+00 | 7.42E-01 |
| **Cpeb2** | 1.49E-02 | 4.25E+00 | 4.245781103 | 1.14E-01 | 2.69E-02 |
| **Entpd2** | 4.26E-03 | 4.21E+00 | 4.205150306 | 3.41E+00 | 8.11E-01 |
| **Dolpp1** | 2.19E-02 | 4.19E+00 | 4.193282893 | 6.73E-01 | 1.60E-01 |
| **Zyx** | 1.84E-02 | 4.15E+00 | 4.154900709 | 1.03E+00 | 2.48E-01 |
| **Fam212b** | 2.80E-02 | 4.15E+00 | 4.14908253 | 7.41E-02 | 1.79E-02 |
| **Ano8** | 4.20E-02 | 4.13E+00 | 4.12887665 | 5.13E-02 | 1.24E-02 |
| **B020031M17Rik** | 3.87E-02 | 4.12E+00 | 4.122934246 | 1.88E+00 | 4.57E-01 |
| **Cers4** | 1.50E-02 | 4.10E+00 | 4.096348985 | 1.11E-01 | 2.70E-02 |
| **Bok** | 2.46E-02 | 4.09E+00 | 4.094116537 | 6.27E-02 | 1.53E-02 |
| **Etfdh** | 1.70E-02 | 4.09E+00 | 4.089693693 | 5.87E-01 | 1.44E-01 |
| **Foxc1** | 2.83E-02 | 4.07E+00 | 4.070048979 | 1.15E+00 | 2.83E-01 |
| **Rrs1** | 3.55E-02 | 4.07E+00 | 4.068776984 | 2.27E+00 | 5.59E-01 |
| **Acadl** | 2.61E-02 | 4.07E+00 | 4.068769386 | 3.04E-01 | 7.47E-02 |
| **Neu1** | 4.52E-02 | 4.06E+00 | 4.055930527 | 6.31E+00 | 1.55E+00 |
| **Mcrip1** | 1.84E-02 | 4.05E+00 | 4.054075797 | 3.09E+00 | 7.62E-01 |
| **Cib2** | 1.42E-02 | 4.05E+00 | 4.052419168 | 4.69E-02 | 1.16E-02 |
| **Slc50a1** | 1.22E-02 | 4.04E+00 | 4.042634989 | 4.21E+00 | 1.04E+00 |
| **Gal3st2b** | 3.45E-02 | 4.04E+00 | 4.042108697 | 4.51E-02 | 1.12E-02 |
| **Slc22a13** | 2.80E-02 | 4.03E+00 | 4.030803077 | 2.89E-01 | 7.16E-02 |
| **Praf2** | 1.33E-02 | 4.01E+00 | 4.005828608 | 7.29E+00 | 1.82E+00 |
| **Cyp2a5** | 2.26E-02 | 4.00E+00 | 4.001458792 | 4.00E-02 | 1.00E-02 |
| **Npdc1** | 2.38E-02 | 3.99E+00 | 3.990046385 | 1.70E+00 | 4.25E-01 |
| **Casp6** | 5.02E-03 | 3.96E+00 | 3.959542914 | 1.78E+00 | 4.49E-01 |
| **Tsen15** | 2.61E-02 | 3.96E+00 | 3.9568301 | 5.91E-01 | 1.49E-01 |
| **Tmem30c** | 4.61E-02 | 3.95E+00 | 3.951357854 | 4.33E-02 | 1.10E-02 |
| **Gm10112** | 3.60E-02 | 3.94E+00 | 3.943762048 | 1.79E+00 | 4.53E-01 |
| **Baiap2l2** | 1.53E-02 | 3.94E+00 | 3.938435035 | 8.81E-01 | 2.24E-01 |
| **Dlk1** | 3.45E-02 | 3.93E+00 | 3.934498324 | 3.93E-02 | 1.00E-02 |
| **Rflnb** | 2.17E-02 | 3.92E+00 | 3.921834777 | 1.08E+00 | 2.76E-01 |
| **Tmem240** | 2.33E-02 | 3.92E+00 | 3.916951655 | 3.92E-02 | 1.00E-02 |
| **Trim12c** | 8.18E-03 | 3.91E+00 | 3.908905324 | 4.84E-01 | 1.24E-01 |
| **1700014D04Rik** | 2.93E-02 | 3.91E+00 | 3.907746001 | 5.51E-02 | 1.41E-02 |
| **Comp** | 1.51E-02 | 3.89E+00 | 3.891204415 | 2.30E-01 | 5.92E-02 |
| **Cbx6** | 2.46E-02 | 3.88E+00 | 3.878565645 | 3.88E-02 | 1.00E-02 |
| **Zfp422** | 4.21E-02 | 3.87E+00 | 3.872989321 | 3.43E-01 | 8.87E-02 |
| **Gstp2** | 1.43E-02 | 3.83E+00 | 3.833840002 | 1.96E+01 | 5.10E+00 |
| **Actr1b** | 2.83E-02 | 3.81E+00 | 3.812040474 | 1.05E+00 | 2.75E-01 |
| **Pls3** | 6.36E-03 | 3.80E+00 | 3.800369932 | 4.20E-01 | 1.11E-01 |
| **Chmp6** | 2.46E-02 | 3.79E+00 | 3.790371433 | 1.62E+00 | 4.27E-01 |
| **Rasgrp4** | 3.47E-02 | 3.78E+00 | 3.784419496 | 2.77E-01 | 7.31E-02 |
| **Wdr81** | 3.18E-02 | 3.78E+00 | 3.780624651 | 1.39E-01 | 3.69E-02 |
| **9930012K11Rik** | 2.59E-02 | 3.76E+00 | 3.764261785 | 6.28E+00 | 1.67E+00 |
| **B3galt6** | 3.24E-02 | 3.75E+00 | 3.753181969 | 1.04E+00 | 2.78E-01 |
| **Cd209e** | 4.83E-02 | 3.72E+00 | 3.722109518 | 6.64E-02 | 1.78E-02 |
| **Gm21560** | 4.85E-02 | 3.72E+00 | 3.715987091 | 6.82E-02 | 1.83E-02 |
| **Orai3** | 3.52E-02 | 3.72E+00 | 3.715925921 | 6.22E-01 | 1.67E-01 |
| **Nes** | 2.88E-02 | 3.70E+00 | 3.696674665 | 8.28E-01 | 2.24E-01 |
| **Ssh3** | 3.81E-02 | 3.64E+00 | 3.639669041 | 7.56E-02 | 2.08E-02 |
| **Rusc1** | 2.82E-02 | 3.63E+00 | 3.626398774 | 5.93E-01 | 1.64E-01 |
| **Rorc** | 3.08E-02 | 3.62E+00 | 3.618859561 | 5.10E-02 | 1.41E-02 |
| **Tfec** | 2.41E-02 | 3.62E+00 | 3.618203103 | 9.23E-02 | 2.55E-02 |
| **Smim24** | 3.04E-02 | 3.61E+00 | 3.61115834 | 2.33E+00 | 6.45E-01 |
| **Espn** | 2.03E-02 | 3.61E+00 | 3.60936749 | 1.14E-01 | 3.16E-02 |
| **Cyb5r1** | 3.44E-02 | 3.61E+00 | 3.606138614 | 4.87E-01 | 1.35E-01 |
| **Zscan5b** | 1.92E-02 | 3.60E+00 | 3.600170141 | 7.03E-01 | 1.95E-01 |
| **Cox8c** | 3.93E-02 | 3.60E+00 | 3.599412694 | 2.44E+00 | 6.78E-01 |
| **Lrfn1** | 2.78E-02 | 3.59E+00 | 3.593972439 | 9.30E-02 | 2.59E-02 |
| **Smarcd3** | 4.17E-02 | 3.58E+00 | 3.584588984 | 1.45E-01 | 4.05E-02 |
| **Olfr847** | 1.49E-02 | 3.58E+00 | 3.57721724 | 4.85E-01 | 1.36E-01 |
| **Fads1** | 3.71E-02 | 3.57E+00 | 3.574633567 | 3.57E-02 | 1.00E-02 |
| **Ehmt2** | 2.82E-02 | 3.57E+00 | 3.574271182 | 4.64E-01 | 1.30E-01 |
| **Dennd1c** | 2.00E-02 | 3.57E+00 | 3.572117615 | 4.27E-02 | 1.19E-02 |
| **Fzd5** | 1.73E-02 | 3.56E+00 | 3.564212328 | 6.88E-01 | 1.93E-01 |
| **Eid2** | 4.88E-02 | 3.56E+00 | 3.562910065 | 6.69E+00 | 1.88E+00 |
| **Prr12** | 9.26E-03 | 3.56E+00 | 3.560250321 | 4.28E-02 | 1.20E-02 |
| **Prrg4** | 2.02E-02 | 3.56E+00 | 3.557363078 | 6.97E-02 | 1.96E-02 |
| **Bglap3** | 4.74E-02 | 3.55E+00 | 3.553412181 | 2.02E+00 | 5.67E-01 |
| **Snx10** | 2.86E-02 | 3.53E+00 | 3.52948271 | 1.07E-01 | 3.04E-02 |
| **Prph** | 2.80E-02 | 3.52E+00 | 3.515715787 | 5.36E-01 | 1.52E-01 |
| **Zfp54** | 4.05E-02 | 3.51E+00 | 3.512509638 | 1.25E-01 | 3.56E-02 |
| **Krt7** | 3.64E-02 | 3.50E+00 | 3.498452031 | 6.79E-02 | 1.94E-02 |
| **Ptrh1** | 1.17E-02 | 3.46E+00 | 3.459463011 | 3.10E+00 | 8.95E-01 |
| **Mmp15** | 2.33E-02 | 3.46E+00 | 3.457339029 | 5.07E-02 | 1.47E-02 |
| **Rbm43** | 2.31E-02 | 3.45E+00 | 3.445261269 | 3.45E-02 | 1.00E-02 |
| **Ormdl2** | 4.78E-02 | 3.44E+00 | 3.439079668 | 1.29E+00 | 3.76E-01 |
| **Rabep2** | 2.98E-02 | 3.43E+00 | 3.432146829 | 2.48E-01 | 7.22E-02 |
| **Csrp1** | 4.91E-02 | 3.42E+00 | 3.424062939 | 1.13E+00 | 3.29E-01 |
| **Dusp2** | 1.15E-02 | 3.42E+00 | 3.418284661 | 1.44E+01 | 4.20E+00 |
| **Ap3b2** | 3.63E-02 | 3.42E+00 | 3.416887165 | 1.11E-01 | 3.26E-02 |
| **Zfyve27** | 2.16E-02 | 3.41E+00 | 3.410941244 | 1.92E-01 | 5.64E-02 |
| **Prkag2os1** | 3.01E-02 | 3.40E+00 | 3.400913278 | 4.86E-01 | 1.43E-01 |
| **Tsen34** | 1.20E-02 | 3.39E+00 | 3.387017182 | 1.31E+00 | 3.87E-01 |
| **Irf2bp2** | 1.84E-02 | 3.39E+00 | 3.386996995 | 1.06E+00 | 3.13E-01 |
| **Gm3776** | 4.91E-02 | 3.39E+00 | 3.386350916 | 4.75E-02 | 1.40E-02 |
| **Nipsnap3b** | 2.45E-02 | 3.38E+00 | 3.384492467 | 9.78E-01 | 2.89E-01 |
| **Vrk3** | 1.71E-02 | 3.37E+00 | 3.371075842 | 1.13E+00 | 3.35E-01 |
| **Ogfr** | 3.13E-02 | 3.37E+00 | 3.369305621 | 9.05E-01 | 2.69E-01 |
| **Rdm1** | 1.44E-02 | 3.36E+00 | 3.357398694 | 1.52E+00 | 4.52E-01 |
| **Fbxl21** | 9.70E-03 | 3.35E+00 | 3.3503279 | 1.03E+00 | 3.07E-01 |
| **Pacsin3** | 3.38E-02 | 3.34E+00 | 3.341784134 | 3.14E-01 | 9.40E-02 |
| **Gli3** | 3.09E-02 | 3.34E+00 | 3.339787363 | 9.98E-02 | 2.99E-02 |
| **Cgrrf1** | 3.21E-02 | 3.34E+00 | 3.339132244 | 4.17E-01 | 1.25E-01 |
| **Cilp2** | 3.74E-02 | 3.34E+00 | 3.338573938 | 8.01E-01 | 2.40E-01 |
| **Hist3h2a** | 6.94E-03 | 3.33E+00 | 3.333229886 | 2.56E+01 | 7.67E+00 |
| **Cited1** | 1.84E-02 | 3.33E+00 | 3.330960792 | 7.68E-01 | 2.31E-01 |
| **Mettl26** | 9.26E-03 | 3.32E+00 | 3.32320867 | 9.64E-01 | 2.90E-01 |
| **1700123O20Rik** | 1.46E-02 | 3.32E+00 | 3.320784641 | 2.12E+00 | 6.39E-01 |
| **Endov** | 1.43E-02 | 3.32E+00 | 3.317392154 | 2.45E-01 | 7.39E-02 |
| **Pigt** | 2.96E-02 | 3.31E+00 | 3.313448879 | 1.84E-01 | 5.56E-02 |
| **Socs2** | 2.24E-02 | 3.31E+00 | 3.312622009 | 2.30E-01 | 6.94E-02 |
| **Lad1** | 3.13E-02 | 3.30E+00 | 3.303804558 | 2.69E-01 | 8.16E-02 |
| **Glmp** | 7.40E-03 | 3.30E+00 | 3.30284419 | 7.13E+00 | 2.16E+00 |
| **Zfp619** | 3.19E-02 | 3.30E+00 | 3.29541866 | 5.96E-02 | 1.81E-02 |
| **Slc25a33** | 1.02E-02 | 3.29E+00 | 3.288442014 | 5.67E-01 | 1.73E-01 |
| **Pomgnt2** | 2.46E-02 | 3.27E+00 | 3.272932635 | 6.89E-01 | 2.11E-01 |
| **Caskin1** | 2.17E-02 | 3.27E+00 | 3.267141212 | 5.87E-02 | 1.80E-02 |
| **Kmt2a** | 9.70E-03 | 3.26E+00 | 3.255335209 | 4.02E-02 | 1.23E-02 |
| **Mrm1** | 1.71E-02 | 3.25E+00 | 3.251087455 | 6.49E-01 | 2.00E-01 |
| **Gm21762** | 4.94E-02 | 3.23E+00 | 3.234395949 | 2.70E+00 | 8.36E-01 |
| **Impdh1** | 1.92E-02 | 3.23E+00 | 3.230680237 | 1.98E-01 | 6.14E-02 |
| **Rap2c** | 2.63E-02 | 3.23E+00 | 3.230537067 | 3.15E-01 | 9.75E-02 |
| **Fgf18** | 2.00E-02 | 3.23E+00 | 3.229556759 | 4.03E-02 | 1.25E-02 |
| **B4galt3** | 3.87E-02 | 3.22E+00 | 3.216769835 | 1.77E+00 | 5.50E-01 |
| **Tppp3** | 2.02E-02 | 3.22E+00 | 3.216495548 | 4.88E+00 | 1.52E+00 |
| **Hspbp1** | 2.91E-02 | 3.20E+00 | 3.203171785 | 3.95E-01 | 1.23E-01 |
| **A930004D18Rik** | 4.10E-02 | 3.19E+00 | 3.194280124 | 1.90E-01 | 5.95E-02 |
| **Pepd** | 1.49E-02 | 3.18E+00 | 3.184476892 | 1.70E-01 | 5.32E-02 |
| **Klhl7** | 2.03E-02 | 3.18E+00 | 3.179577704 | 1.08E-01 | 3.39E-02 |
| **Marcksl1** | 1.42E-02 | 3.17E+00 | 3.173286549 | 1.34E+00 | 4.23E-01 |
| **Tmem160** | 8.18E-03 | 3.17E+00 | 3.172271274 | 4.24E+00 | 1.34E+00 |
| **Smco2** | 2.03E-02 | 3.17E+00 | 3.165729379 | 4.97E-01 | 1.57E-01 |
| **Prnp** | 2.46E-02 | 3.15E+00 | 3.152438461 | 4.53E-01 | 1.44E-01 |
| **Psph** | 1.61E-02 | 3.15E+00 | 3.145287124 | 9.39E-01 | 2.98E-01 |
| **Ppif** | 2.24E-02 | 3.12E+00 | 3.117112215 | 7.25E+00 | 2.33E+00 |
| **Gpr83** | 4.81E-02 | 3.12E+00 | 3.115370423 | 7.85E-01 | 2.52E-01 |
| **Dhrs7b** | 7.65E-03 | 3.12E+00 | 3.115145371 | 8.32E-01 | 2.67E-01 |
| **Slc5a2** | 7.74E-03 | 3.11E+00 | 3.110034757 | 1.44E+00 | 4.63E-01 |
| **Etv3** | 4.07E-02 | 3.11E+00 | 3.106090851 | 1.59E-01 | 5.11E-02 |
| **Pvr** | 3.64E-02 | 3.11E+00 | 3.106051085 | 1.45E-01 | 4.67E-02 |
| **Klf3** | 3.09E-02 | 3.10E+00 | 3.103622688 | 9.84E-02 | 3.17E-02 |
| **Mak16** | 9.70E-03 | 3.10E+00 | 3.100083885 | 1.34E+00 | 4.31E-01 |
| **Dpagt1** | 1.36E-02 | 3.10E+00 | 3.097649376 | 9.57E-01 | 3.09E-01 |
| **Plp2** | 1.38E-02 | 3.09E+00 | 3.094011801 | 1.52E+00 | 4.90E-01 |
| **Grhl3** | 3.47E-02 | 3.08E+00 | 3.081246205 | 6.37E-02 | 2.07E-02 |
| **Yjefn3** | 2.92E-02 | 3.08E+00 | 3.080594446 | 3.26E-01 | 1.06E-01 |
| **Zrsr1** | 4.01E-02 | 3.08E+00 | 3.078330987 | 3.02E-01 | 9.81E-02 |
| **Ano7** | 1.79E-02 | 3.08E+00 | 3.077450933 | 8.16E-02 | 2.65E-02 |
| **Angptl6** | 2.23E-02 | 3.07E+00 | 3.074073993 | 1.59E+00 | 5.17E-01 |
| **Gm20390** | 3.52E-02 | 3.07E+00 | 3.065823841 | 2.92E-01 | 9.52E-02 |
| **Zfp800** | 1.92E-02 | 3.06E+00 | 3.057918602 | 7.64E-02 | 2.50E-02 |
| **Selenom** | 3.71E-02 | 3.05E+00 | 3.053115079 | 1.31E+00 | 4.30E-01 |
| **Zfp367** | 4.85E-02 | 3.05E+00 | 3.047757615 | 3.28E-01 | 1.08E-01 |
| **Cxcl14** | 3.52E-02 | 3.04E+00 | 3.044890413 | 1.55E+00 | 5.08E-01 |
| **Pim2** | 2.03E-02 | 3.04E+00 | 3.042177704 | 2.49E+00 | 8.18E-01 |
| **Isyna1** | 2.82E-02 | 3.04E+00 | 3.036024294 | 2.01E+01 | 6.61E+00 |
| **Cbx7** | 3.88E-02 | 3.04E+00 | 3.035347014 | 1.25E-01 | 4.12E-02 |
| **Rai1** | 1.71E-02 | 3.02E+00 | 3.020368418 | 8.11E-02 | 2.68E-02 |
| **Fcgrt** | 2.84E-02 | 3.02E+00 | 3.015631479 | 3.73E-02 | 1.24E-02 |
| **Tmx1** | 1.51E-02 | 3.02E+00 | 3.015135135 | 9.63E-01 | 3.19E-01 |
| **Ly6a** | 2.92E-02 | 3.00E+00 | 3.001884633 | 3.71E+00 | 1.24E+00 |
| **Tmed4** | 7.65E-03 | 3.00E+00 | 3.001195006 | 3.70E+00 | 1.23E+00 |
| **P2ry1** | 4.84E-02 | 3.00E+00 | 2.997227345 | 7.99E-01 | 2.67E-01 |
| **G2e3** | 7.40E-03 | 2.99E+00 | 2.9941738 | 9.28E-01 | 3.10E-01 |
| **E130309D02Rik** | 3.79E-02 | 2.99E+00 | 2.994031654 | 5.53E-01 | 1.85E-01 |
| **Gstp1** | 1.09E-02 | 2.99E+00 | 2.989984464 | 7.40E+01 | 2.47E+01 |
| **Dpp7** | 7.02E-03 | 2.99E+00 | 2.988997806 | 5.04E+00 | 1.69E+00 |
| **Scrn2** | 2.50E-02 | 2.98E+00 | 2.984481361 | 1.72E+00 | 5.75E-01 |
| **Kdf1** | 2.83E-02 | 2.98E+00 | 2.983704141 | 2.61E+00 | 8.74E-01 |
| **Tmie** | 3.16E-02 | 2.97E+00 | 2.973345743 | 3.47E-02 | 1.17E-02 |
| **Nxt2** | 2.00E-02 | 2.97E+00 | 2.969333215 | 7.37E-01 | 2.48E-01 |
| **Ubtfl1** | 3.72E-02 | 2.97E+00 | 2.96831326 | 2.97E-02 | 1.00E-02 |
| **Slc16a11** | 1.53E-02 | 2.97E+00 | 2.968048614 | 2.34E+00 | 7.89E-01 |
| **Serpinb6c** | 4.03E-02 | 2.96E+00 | 2.964688769 | 1.53E-01 | 5.16E-02 |
| **Hdhd3** | 1.92E-02 | 2.95E+00 | 2.949548995 | 1.74E+00 | 5.90E-01 |
| **Nme2** | 4.39E-02 | 2.95E+00 | 2.948785452 | 1.31E+00 | 4.45E-01 |
| **Ctsl** | 8.44E-03 | 2.93E+00 | 2.929525766 | 1.92E+00 | 6.54E-01 |
| **Dennd6b** | 3.21E-02 | 2.93E+00 | 2.925273753 | 1.62E-01 | 5.52E-02 |
| **Nxnl2** | 1.59E-02 | 2.92E+00 | 2.919451251 | 1.31E+00 | 4.47E-01 |
| **Ccdc126** | 2.29E-02 | 2.92E+00 | 2.918569981 | 2.81E-01 | 9.64E-02 |
| **Dpf2** | 8.85E-03 | 2.91E+00 | 2.909553147 | 3.90E+00 | 1.34E+00 |
| **Hist1h2bk** | 4.88E-03 | 2.91E+00 | 2.909055715 | 2.94E+01 | 1.01E+01 |
| **Gnb5** | 1.09E-02 | 2.91E+00 | 2.906585528 | 9.20E-01 | 3.17E-01 |
| **Cyp2b19** | 4.68E-02 | 2.90E+00 | 2.903168669 | 7.82E-02 | 2.69E-02 |
| **Tmigd1** | 4.74E-02 | 2.90E+00 | 2.896349938 | 3.40E-01 | 1.17E-01 |
| **Bcor** | 2.17E-02 | 2.89E+00 | 2.888743554 | 1.17E-01 | 4.04E-02 |
| **Dusp22** | 2.93E-02 | 2.88E+00 | 2.881308007 | 1.50E-01 | 5.21E-02 |
| **Ttc25** | 2.10E-02 | 2.88E+00 | 2.879191722 | 3.44E-02 | 1.20E-02 |
| **Gpat2** | 1.53E-02 | 2.87E+00 | 2.87211732 | 8.57E-01 | 2.98E-01 |
| **Snrpb** | 5.98E-03 | 2.87E+00 | 2.869440503 | 1.41E+01 | 4.91E+00 |
| **Mthfsd** | 9.69E-03 | 2.87E+00 | 2.867748616 | 5.59E-01 | 1.95E-01 |
| **Rarg** | 4.13E-02 | 2.85E+00 | 2.849532741 | 3.86E-02 | 1.35E-02 |
| **Zc3hc1** | 1.33E-02 | 2.83E+00 | 2.834531722 | 8.85E-01 | 3.12E-01 |
| **Cbx8** | 1.73E-02 | 2.83E+00 | 2.825724003 | 1.24E+00 | 4.38E-01 |
| **Cldn34c1** | 1.84E-02 | 2.82E+00 | 2.819428465 | 2.70E-01 | 9.57E-02 |
| **Il13ra1** | 3.46E-02 | 2.81E+00 | 2.813062325 | 3.65E-02 | 1.30E-02 |
| **Vhl** | 2.19E-02 | 2.81E+00 | 2.811576128 | 6.12E-01 | 2.18E-01 |
| **Vma21-ps** | 2.17E-02 | 2.81E+00 | 2.806438559 | 7.89E+00 | 2.81E+00 |
| **Sac3d1** | 2.02E-02 | 2.80E+00 | 2.801364691 | 1.76E+01 | 6.27E+00 |
| **Lemd3** | 3.29E-02 | 2.80E+00 | 2.801164445 | 3.57E-02 | 1.28E-02 |
| **Upk3b** | 4.49E-02 | 2.79E+00 | 2.79327017 | 8.44E-01 | 3.02E-01 |
| **Calml4** | 4.49E-02 | 2.79E+00 | 2.79294586 | 4.41E-01 | 1.58E-01 |
| **Uqcrc1** | 1.74E-02 | 2.78E+00 | 2.783174428 | 9.20E+00 | 3.30E+00 |
| **Colgalt1** | 4.16E-02 | 2.78E+00 | 2.778569286 | 1.81E-01 | 6.52E-02 |
| **Cystm1** | 2.42E-02 | 2.78E+00 | 2.775894601 | 4.82E-01 | 1.74E-01 |
| **Dnajb9** | 1.61E-02 | 2.77E+00 | 2.771650982 | 1.81E+00 | 6.53E-01 |
| **Aifm2** | 4.35E-02 | 2.77E+00 | 2.765325127 | 2.58E-01 | 9.33E-02 |
| **Mettl22** | 2.39E-02 | 2.76E+00 | 2.763576269 | 3.05E-01 | 1.10E-01 |
| **Arf2** | 7.09E-03 | 2.76E+00 | 2.762951237 | 7.48E-01 | 2.71E-01 |
| **B3galt4** | 4.56E-02 | 2.76E+00 | 2.760126909 | 2.49E+00 | 9.00E-01 |
| **Dnajc12** | 4.57E-02 | 2.76E+00 | 2.760023609 | 7.16E-02 | 2.59E-02 |
| **Tcp11l1** | 1.14E-02 | 2.76E+00 | 2.755795365 | 4.91E-01 | 1.78E-01 |
| **Trim25** | 4.96E-02 | 2.76E+00 | 2.755535504 | 3.39E-02 | 1.23E-02 |
| **Akr1c19** | 1.44E-02 | 2.75E+00 | 2.754956853 | 8.45E-01 | 3.07E-01 |
| **Camk1** | 2.41E-02 | 2.75E+00 | 2.749001759 | 9.08E-01 | 3.30E-01 |
| **Gas2l1** | 3.67E-02 | 2.75E+00 | 2.745920959 | 6.98E-01 | 2.54E-01 |
| **Traf2** | 3.70E-02 | 2.74E+00 | 2.740198758 | 2.27E-01 | 8.28E-02 |
| **Pim1** | 3.21E-02 | 2.71E+00 | 2.709169655 | 1.47E-01 | 5.44E-02 |
| **Coq10a** | 3.21E-02 | 2.71E+00 | 2.705997069 | 3.36E+00 | 1.24E+00 |
| **Ufc1** | 9.70E-03 | 2.70E+00 | 2.702362606 | 1.07E+01 | 3.95E+00 |
| **Pou6f1** | 1.92E-02 | 2.70E+00 | 2.698296816 | 2.98E-02 | 1.10E-02 |
| **Ing4** | 2.17E-02 | 2.70E+00 | 2.695689948 | 1.74E+00 | 6.44E-01 |
| **Tsr2** | 5.73E-03 | 2.69E+00 | 2.694767136 | 4.27E+00 | 1.59E+00 |
| **Fam136a** | 6.94E-03 | 2.69E+00 | 2.693160491 | 6.39E+00 | 2.37E+00 |
| **Relb** | 4.91E-02 | 2.69E+00 | 2.690219178 | 2.22E-01 | 8.27E-02 |
| **Commd7** | 1.52E-02 | 2.69E+00 | 2.6881235 | 2.13E+00 | 7.92E-01 |
| **Cspg5** | 2.62E-02 | 2.68E+00 | 2.677752948 | 5.37E-01 | 2.00E-01 |
| **Card19** | 2.62E-02 | 2.67E+00 | 2.671103414 | 2.24E+00 | 8.38E-01 |
| **Pdxk** | 3.87E-02 | 2.67E+00 | 2.670770156 | 7.90E-02 | 2.96E-02 |
| **Tomm5** | 2.68E-02 | 2.67E+00 | 2.669406875 | 2.03E+00 | 7.62E-01 |
| **Ccdc158** | 1.62E-02 | 2.67E+00 | 2.668932617 | 8.83E-01 | 3.31E-01 |
| **Acrbp** | 3.22E-02 | 2.66E+00 | 2.664041037 | 3.20E-02 | 1.20E-02 |
| **Gm7334** | 4.26E-03 | 2.66E+00 | 2.65847738 | 2.53E+01 | 9.53E+00 |
| **Tex19.1** | 3.21E-02 | 2.65E+00 | 2.652105658 | 5.35E+01 | 2.02E+01 |
| **Vamp5** | 2.04E-02 | 2.65E+00 | 2.64960637 | 1.63E-01 | 6.16E-02 |
| **Ggnbp2** | 1.77E-02 | 2.65E+00 | 2.647598845 | 8.73E-01 | 3.30E-01 |
| **Grwd1** | 2.19E-02 | 2.64E+00 | 2.641256858 | 5.18E+00 | 1.96E+00 |
| **Xkr8** | 3.50E-02 | 2.64E+00 | 2.639576773 | 1.12E+00 | 4.25E-01 |
| **Slc9a8** | 2.50E-02 | 2.62E+00 | 2.624284766 | 1.63E-01 | 6.21E-02 |
| **Fam178b** | 2.10E-02 | 2.62E+00 | 2.620645655 | 2.86E-01 | 1.09E-01 |
| **AI854703** | 4.29E-02 | 2.62E+00 | 2.61608767 | 1.45E+00 | 5.52E-01 |
| **Cuedc2** | 1.76E-02 | 2.62E+00 | 2.615883617 | 5.46E-01 | 2.09E-01 |
| **Tnfrsf12a** | 2.43E-02 | 2.62E+00 | 2.615044603 | 3.52E+00 | 1.35E+00 |
| **Simc1** | 9.96E-03 | 2.61E+00 | 2.612435955 | 4.32E-01 | 1.65E-01 |
| **H2-K1** | 1.69E-02 | 2.61E+00 | 2.609099009 | 7.32E+00 | 2.80E+00 |
| **Eral1** | 1.41E-02 | 2.61E+00 | 2.606974342 | 3.42E+00 | 1.31E+00 |
| **Hist1h4i** | 2.99E-02 | 2.60E+00 | 2.602493054 | 7.92E+00 | 3.04E+00 |
| **Clp1** | 3.42E-02 | 2.60E+00 | 2.596493826 | 7.84E+00 | 3.02E+00 |
| **Hist1h2ap** | 4.74E-02 | 2.59E+00 | 2.59233375 | 1.93E+01 | 7.43E+00 |
| **Gcsh** | 6.30E-03 | 2.58E+00 | 2.580031605 | 2.02E+00 | 7.83E-01 |
| **Urgcp** | 2.43E-02 | 2.57E+00 | 2.567467191 | 5.22E-02 | 2.03E-02 |
| **Tmcc1** | 1.01E-02 | 2.57E+00 | 2.56693159 | 1.14E+00 | 4.43E-01 |
| **Tnip2** | 3.68E-02 | 2.56E+00 | 2.564779849 | 2.76E-01 | 1.08E-01 |
| **Mplkip** | 6.30E-03 | 2.56E+00 | 2.56302556 | 4.70E+00 | 1.83E+00 |
| **Osgin2** | 2.25E-02 | 2.56E+00 | 2.557353529 | 3.59E-01 | 1.40E-01 |
| **Hist2h2aa1** | 3.30E-03 | 2.55E+00 | 2.55155319 | 2.82E+02 | 1.10E+02 |
| **Pfkl** | 2.33E-02 | 2.55E+00 | 2.547763746 | 6.74E-01 | 2.65E-01 |
| **Tspan4** | 4.61E-02 | 2.55E+00 | 2.545147594 | 1.35E-01 | 5.32E-02 |
| **Rasd2** | 4.03E-02 | 2.54E+00 | 2.542044154 | 6.76E-01 | 2.66E-01 |
| **Zfp36l2** | 3.30E-03 | 2.54E+00 | 2.535947837 | 3.32E+00 | 1.31E+00 |
| **Vps26b** | 4.10E-02 | 2.53E+00 | 2.532761686 | 1.78E-01 | 7.04E-02 |
| **Gipc1** | 1.92E-02 | 2.53E+00 | 2.530679729 | 8.32E-01 | 3.29E-01 |
| **Art1** | 2.42E-02 | 2.53E+00 | 2.52903103 | 4.54E-01 | 1.79E-01 |
| **Prkra** | 4.82E-02 | 2.53E+00 | 2.52745563 | 2.14E-01 | 8.47E-02 |
| **Nop16** | 1.43E-02 | 2.53E+00 | 2.52679682 | 2.84E+00 | 1.12E+00 |
| **Klf10** | 1.33E-02 | 2.52E+00 | 2.516759959 | 7.00E-01 | 2.78E-01 |
| **Smim1** | 2.82E-02 | 2.52E+00 | 2.51606037 | 1.91E+00 | 7.59E-01 |
| **Glrb** | 4.23E-02 | 2.51E+00 | 2.513617527 | 4.85E-02 | 1.93E-02 |
| **Zfp292** | 2.57E-02 | 2.51E+00 | 2.507815363 | 5.45E-02 | 2.17E-02 |
| **Sqstm1** | 3.01E-02 | 2.50E+00 | 2.501272637 | 3.10E+00 | 1.24E+00 |
| **D930020B18Rik** | 3.49E-02 | 2.50E+00 | 2.498707216 | 2.96E-01 | 1.18E-01 |
| **Coq8b** | 2.70E-02 | 2.50E+00 | 2.497789985 | 3.90E-01 | 1.56E-01 |
| **Tctn3** | 2.99E-02 | 2.49E+00 | 2.490138597 | 3.75E-01 | 1.51E-01 |
| **Cacfd1** | 3.53E-02 | 2.48E+00 | 2.483001209 | 2.59E+00 | 1.04E+00 |
| **Syngr2** | 3.12E-02 | 2.48E+00 | 2.482267358 | 2.63E+01 | 1.06E+01 |
| **Pde7a** | 4.96E-02 | 2.48E+00 | 2.479870705 | 3.22E-02 | 1.30E-02 |
| **Slc35e1** | 4.85E-02 | 2.48E+00 | 2.475659856 | 2.02E-01 | 8.14E-02 |
| **P3h3** | 4.10E-02 | 2.48E+00 | 2.47556613 | 3.99E-02 | 1.61E-02 |
| **Gadd45a** | 4.16E-02 | 2.47E+00 | 2.472077092 | 2.94E+00 | 1.19E+00 |
| **Rnf149** | 4.26E-02 | 2.47E+00 | 2.466456507 | 4.94E-01 | 2.00E-01 |
| **Zglp1** | 1.48E-02 | 2.46E+00 | 2.463750014 | 7.35E+00 | 2.98E+00 |
| **Tars2** | 2.27E-02 | 2.46E+00 | 2.462321388 | 6.41E-01 | 2.60E-01 |
| **Wdr45b** | 1.33E-02 | 2.46E+00 | 2.46131777 | 1.15E+00 | 4.67E-01 |
| **Tcea2** | 1.61E-02 | 2.46E+00 | 2.455043137 | 1.78E+00 | 7.24E-01 |
| **Zfp809** | 2.40E-02 | 2.45E+00 | 2.454708459 | 9.98E-01 | 4.07E-01 |
| **Hsd17b10** | 4.26E-03 | 2.45E+00 | 2.454327432 | 4.14E+01 | 1.69E+01 |
| **Itgav** | 4.50E-02 | 2.45E+00 | 2.452812617 | 3.91E-02 | 1.59E-02 |
| **Osgep** | 3.19E-02 | 2.45E+00 | 2.448142682 | 7.95E-01 | 3.25E-01 |
| **Rbm5** | 3.52E-03 | 2.45E+00 | 2.447699384 | 2.48E+00 | 1.01E+00 |
| **Mboat7** | 2.19E-02 | 2.44E+00 | 2.440262946 | 5.43E-01 | 2.23E-01 |
| **Cenpt** | 2.25E-02 | 2.44E+00 | 2.439518365 | 8.76E+00 | 3.59E+00 |
| **Ddx17** | 2.08E-02 | 2.44E+00 | 2.439188406 | 9.36E-01 | 3.84E-01 |
| **Fam216a** | 6.30E-03 | 2.44E+00 | 2.438854824 | 7.89E+00 | 3.23E+00 |
| **Mrpl20** | 7.44E-03 | 2.44E+00 | 2.438178195 | 5.73E+00 | 2.35E+00 |
| **Lats2** | 1.51E-02 | 2.43E+00 | 2.432207433 | 3.43E-02 | 1.41E-02 |
| **1700016K19Rik** | 1.84E-02 | 2.43E+00 | 2.429260361 | 1.82E+00 | 7.49E-01 |
| **Acat2** | 3.74E-02 | 2.43E+00 | 2.426078553 | 1.69E+00 | 6.95E-01 |
| **Pnrc2** | 4.96E-03 | 2.42E+00 | 2.424123235 | 1.07E+01 | 4.42E+00 |
| **Hist1h4h** | 1.76E-02 | 2.42E+00 | 2.419895409 | 7.04E+01 | 2.91E+01 |
| **Lap3** | 1.49E-02 | 2.42E+00 | 2.419376006 | 9.99E-01 | 4.13E-01 |
| **Fam76a** | 2.25E-02 | 2.41E+00 | 2.41130083 | 3.30E-01 | 1.37E-01 |
| **Abca2** | 4.39E-02 | 2.40E+00 | 2.403319049 | 3.39E-01 | 1.41E-01 |
| **Eps8l1** | 3.55E-02 | 2.40E+00 | 2.400307536 | 4.61E-02 | 1.92E-02 |
| **Grk1** | 2.64E-02 | 2.40E+00 | 2.399504979 | 2.74E-02 | 1.14E-02 |
| **Gpn3** | 1.96E-02 | 2.40E+00 | 2.397949461 | 2.31E+00 | 9.64E-01 |
| **Cbfb** | 4.98E-02 | 2.40E+00 | 2.397883604 | 1.65E-01 | 6.88E-02 |
| **Prrc2b** | 2.43E-02 | 2.39E+00 | 2.390116384 | 2.96E-02 | 1.24E-02 |
| **Vrk2** | 2.94E-02 | 2.38E+00 | 2.377898364 | 9.55E-02 | 4.02E-02 |
| **Cyc1** | 1.58E-02 | 2.38E+00 | 2.375733289 | 1.85E+01 | 7.80E+00 |
| **Slc35b2** | 4.29E-02 | 2.37E+00 | 2.372374587 | 1.73E+01 | 7.29E+00 |
| **Rab24** | 1.36E-02 | 2.37E+00 | 2.370190332 | 3.57E+00 | 1.50E+00 |
| **Polr2h** | 4.26E-03 | 2.37E+00 | 2.368874619 | 1.25E+01 | 5.27E+00 |
| **Chac2** | 7.76E-03 | 2.37E+00 | 2.367883967 | 1.84E+00 | 7.76E-01 |
| **Irf3** | 1.62E-02 | 2.37E+00 | 2.36534005 | 8.62E+00 | 3.64E+00 |
| **Chchd4** | 2.52E-02 | 2.37E+00 | 2.365210351 | 1.26E+00 | 5.34E-01 |
| **Acaa1b** | 4.87E-02 | 2.36E+00 | 2.362881905 | 5.41E-01 | 2.29E-01 |
| **Slc25a51** | 1.45E-02 | 2.36E+00 | 2.361148132 | 6.46E+00 | 2.74E+00 |
| **H2afz** | 2.04E-03 | 2.36E+00 | 2.358516402 | 8.99E+01 | 3.81E+01 |
| **Zfp637** | 2.59E-02 | 2.35E+00 | 2.354447132 | 2.45E+00 | 1.04E+00 |
| **Calhm2** | 4.45E-02 | 2.35E+00 | 2.353379556 | 2.35E-02 | 1.00E-02 |
| **Yars2** | 8.26E-03 | 2.35E+00 | 2.35137502 | 1.91E+00 | 8.13E-01 |
| **Ostm1** | 2.38E-02 | 2.35E+00 | 2.348270969 | 1.70E-01 | 7.24E-02 |
| **Sat1** | 4.26E-03 | 2.35E+00 | 2.34571691 | 2.75E+01 | 1.17E+01 |
| **Ptar1** | 1.65E-02 | 2.34E+00 | 2.341840824 | 4.69E-01 | 2.00E-01 |
| **M1ap** | 4.11E-02 | 2.34E+00 | 2.340452178 | 4.93E-02 | 2.11E-02 |
| **2300009A05Rik** | 9.26E-03 | 2.33E+00 | 2.332467178 | 4.15E+00 | 1.78E+00 |
| **Fads3** | 4.27E-02 | 2.33E+00 | 2.330674738 | 8.18E-01 | 3.51E-01 |
| **Pdpn** | 4.87E-02 | 2.33E+00 | 2.328320402 | 1.00E+00 | 4.29E-01 |
| **D030056L22Rik** | 3.38E-02 | 2.33E+00 | 2.327749397 | 1.44E+00 | 6.20E-01 |
| **Fam173a** | 4.47E-02 | 2.33E+00 | 2.326604792 | 7.61E+00 | 3.27E+00 |
| **C1qtnf4** | 4.57E-02 | 2.33E+00 | 2.32532086 | 1.01E+00 | 4.33E-01 |
| **Cryzl1** | 1.77E-02 | 2.33E+00 | 2.325144835 | 4.57E-01 | 1.96E-01 |
| **Cacybp** | 8.64E-03 | 2.32E+00 | 2.324043898 | 3.05E+00 | 1.31E+00 |
| **Rwdd2a** | 7.66E-03 | 2.32E+00 | 2.32162011 | 1.92E+01 | 8.26E+00 |
| **Sfn** | 3.33E-02 | 2.32E+00 | 2.320119101 | 1.19E+01 | 5.14E+00 |
| **Tmed6** | 4.48E-02 | 2.32E+00 | 2.31769845 | 8.15E-02 | 3.52E-02 |
| **Zgpat** | 4.16E-02 | 2.31E+00 | 2.311098872 | 3.01E-01 | 1.30E-01 |
| **Piwil2** | 4.10E-02 | 2.30E+00 | 2.304646933 | 2.13E-01 | 9.23E-02 |
| **Alg6** | 2.39E-02 | 2.30E+00 | 2.299566822 | 4.56E-02 | 1.98E-02 |
| **Pkp2** | 4.79E-02 | 2.30E+00 | 2.297609848 | 1.23E-01 | 5.34E-02 |
| **Fam161b** | 2.17E-02 | 2.30E+00 | 2.297579527 | 4.91E-01 | 2.14E-01 |
| **Ccdc27** | 3.93E-02 | 2.30E+00 | 2.297465317 | 6.25E-01 | 2.72E-01 |
| **Psmb11** | 4.03E-02 | 2.29E+00 | 2.290635551 | 1.09E+00 | 4.78E-01 |
| **Eid1** | 2.45E-02 | 2.29E+00 | 2.288271239 | 2.15E+00 | 9.38E-01 |
| **Tmx2** | 1.11E-02 | 2.29E+00 | 2.285564826 | 3.46E+00 | 1.51E+00 |
| **Gemin2** | 1.62E-02 | 2.28E+00 | 2.282072053 | 1.71E+00 | 7.51E-01 |
| **Smad5** | 3.42E-02 | 2.28E+00 | 2.281877133 | 3.65E-01 | 1.60E-01 |
| **Gm28042** | 4.00E-02 | 2.28E+00 | 2.278749371 | 7.59E-02 | 3.33E-02 |
| **Gpaa1** | 1.49E-02 | 2.28E+00 | 2.276929593 | 5.60E+00 | 2.46E+00 |
| **Mydgf** | 1.59E-02 | 2.27E+00 | 2.273669293 | 3.57E+00 | 1.57E+00 |
| **Ppcs** | 2.97E-02 | 2.27E+00 | 2.269182746 | 8.20E-01 | 3.61E-01 |
| **Crisp1** | 4.18E-02 | 2.27E+00 | 2.265269466 | 2.48E-02 | 1.09E-02 |
| **Prmt5** | 4.43E-02 | 2.26E+00 | 2.262552945 | 2.09E+00 | 9.24E-01 |
| **Mapk13** | 1.89E-02 | 2.26E+00 | 2.261860597 | 2.74E+00 | 1.21E+00 |
| **Sh3tc1** | 3.88E-02 | 2.26E+00 | 2.261091055 | 6.02E-02 | 2.66E-02 |
| **Acy1** | 4.67E-02 | 2.26E+00 | 2.260895801 | 5.83E-01 | 2.58E-01 |
| **Pdgfa** | 1.49E-02 | 2.26E+00 | 2.260773782 | 7.72E-01 | 3.42E-01 |
| **Gpr55** | 2.92E-02 | 2.26E+00 | 2.259579268 | 2.76E-02 | 1.22E-02 |
| **Crtap** | 4.53E-02 | 2.26E+00 | 2.257733832 | 5.00E-01 | 2.21E-01 |
| **Cln3** | 4.60E-02 | 2.26E+00 | 2.257107535 | 6.89E-01 | 3.05E-01 |
| **Ak1** | 1.75E-02 | 2.25E+00 | 2.249442709 | 2.25E-02 | 1.00E-02 |
| **Edc3** | 2.82E-02 | 2.25E+00 | 2.24906341 | 1.47E-01 | 6.54E-02 |
| **B230219D22Rik** | 4.88E-03 | 2.25E+00 | 2.247495629 | 1.75E+00 | 7.77E-01 |
| **Nabp1** | 1.05E-02 | 2.24E+00 | 2.238188752 | 1.25E+00 | 5.60E-01 |
| **Pi4k2a** | 4.47E-02 | 2.23E+00 | 2.234711254 | 1.92E+00 | 8.61E-01 |
| **Col6a2** | 2.82E-02 | 2.23E+00 | 2.234296964 | 2.46E-02 | 1.10E-02 |
| **Myl12b** | 3.30E-03 | 2.22E+00 | 2.224196694 | 4.51E+00 | 2.03E+00 |
| **Fyttd1** | 9.89E-03 | 2.22E+00 | 2.222142437 | 9.93E-01 | 4.47E-01 |
| **Coil** | 2.26E-02 | 2.22E+00 | 2.218081794 | 8.67E-01 | 3.91E-01 |
| **Nkiras2** | 1.84E-02 | 2.22E+00 | 2.217868573 | 1.45E+00 | 6.53E-01 |
| **Mill2** | 2.08E-02 | 2.22E+00 | 2.217076174 | 2.44E-02 | 1.10E-02 |
| **Rab25** | 3.56E-02 | 2.22E+00 | 2.21689173 | 4.11E+00 | 1.86E+00 |
| **Sgpl1** | 2.91E-02 | 2.22E+00 | 2.216380812 | 5.04E-02 | 2.27E-02 |
| **Shisa5** | 1.61E-02 | 2.21E+00 | 2.214160464 | 5.28E+00 | 2.39E+00 |
| **Smpd2** | 3.79E-02 | 2.21E+00 | 2.213199184 | 2.59E+00 | 1.17E+00 |
| **Zfp644** | 1.69E-02 | 2.21E+00 | 2.212280616 | 4.93E-01 | 2.23E-01 |
| **Unc119b** | 4.88E-02 | 2.21E+00 | 2.212129085 | 5.53E-01 | 2.50E-01 |
| **Xpnpep1** | 4.59E-02 | 2.21E+00 | 2.211392429 | 6.64E-02 | 3.00E-02 |
| **Psg16** | 9.26E-03 | 2.21E+00 | 2.207047628 | 6.87E-01 | 3.11E-01 |
| **Aaed1** | 4.52E-02 | 2.21E+00 | 2.205399865 | 8.22E-02 | 3.73E-02 |
| **Tusc2** | 3.21E-02 | 2.20E+00 | 2.203331606 | 7.42E+00 | 3.37E+00 |
| **D830044I16Rik** | 1.73E-02 | 2.20E+00 | 2.202685548 | 6.45E-01 | 2.93E-01 |
| **Dbn1** | 2.64E-02 | 2.20E+00 | 2.198302938 | 2.05E-01 | 9.34E-02 |
| **Id3** | 3.04E-02 | 2.20E+00 | 2.196155995 | 8.20E+01 | 3.73E+01 |
| **Zbtb25** | 2.99E-02 | 2.19E+00 | 2.194194098 | 1.54E+00 | 7.04E-01 |
| **Commd4** | 1.95E-02 | 2.19E+00 | 2.193604751 | 2.44E+01 | 1.11E+01 |
| **Abat** | 3.49E-02 | 2.19E+00 | 2.190161869 | 9.93E-02 | 4.53E-02 |
| **Adsl** | 2.15E-02 | 2.18E+00 | 2.182835343 | 2.89E+00 | 1.32E+00 |
| **Mcl1** | 2.36E-02 | 2.18E+00 | 2.177046884 | 3.57E+00 | 1.64E+00 |
| **Fkbp6** | 9.70E-03 | 2.18E+00 | 2.175409257 | 1.53E+00 | 7.01E-01 |
| **Ell3** | 1.62E-02 | 2.17E+00 | 2.173478516 | 1.57E+01 | 7.25E+00 |
| **Ccne1** | 3.21E-02 | 2.17E+00 | 2.171368676 | 3.61E+00 | 1.66E+00 |
| **Ric8a** | 4.20E-02 | 2.17E+00 | 2.17044362 | 7.56E-01 | 3.48E-01 |
| **9530077C05Rik** | 4.27E-02 | 2.17E+00 | 2.170121333 | 6.95E-02 | 3.20E-02 |
| **Rras** | 2.62E-02 | 2.17E+00 | 2.169927614 | 1.49E+00 | 6.88E-01 |
| **Mpnd** | 4.37E-02 | 2.17E+00 | 2.168329375 | 2.74E+00 | 1.27E+00 |
| **Cuta** | 9.65E-03 | 2.17E+00 | 2.168165271 | 4.08E+01 | 1.88E+01 |
| **Tfeb** | 2.17E-02 | 2.17E+00 | 2.166591656 | 2.37E-02 | 1.10E-02 |
| **Zbtb43** | 4.87E-02 | 2.16E+00 | 2.15710333 | 7.02E-01 | 3.26E-01 |
| **Lingo1** | 2.74E-02 | 2.16E+00 | 2.157091398 | 2.95E-02 | 1.37E-02 |
| **Cc2d2a** | 4.60E-02 | 2.16E+00 | 2.156096891 | 5.74E-02 | 2.66E-02 |
| **Zfp263** | 2.92E-02 | 2.16E+00 | 2.155870708 | 1.97E+00 | 9.15E-01 |
| **Fahd2a** | 9.73E-03 | 2.15E+00 | 2.149804956 | 5.35E+00 | 2.49E+00 |
| **Rhebl1** | 4.13E-02 | 2.15E+00 | 2.145751631 | 8.21E+00 | 3.83E+00 |
| **Chkb** | 1.72E-02 | 2.14E+00 | 2.143621877 | 6.59E-01 | 3.07E-01 |
| **Lyrm7** | 4.04E-02 | 2.14E+00 | 2.143406602 | 2.25E-01 | 1.05E-01 |
| **2210016L21Rik** | 2.08E-02 | 2.14E+00 | 2.143161247 | 1.27E+00 | 5.93E-01 |
| **S100pbp** | 3.12E-02 | 2.14E+00 | 2.141518953 | 2.70E-01 | 1.26E-01 |
| **Mpp1** | 4.28E-02 | 2.14E+00 | 2.139351233 | 4.14E-01 | 1.94E-01 |
| **Epb42** | 2.12E-02 | 2.14E+00 | 2.137293174 | 8.76E-01 | 4.10E-01 |
| **Cox11** | 3.22E-02 | 2.13E+00 | 2.133789822 | 1.56E+00 | 7.31E-01 |
| **Tmod1** | 3.07E-02 | 2.13E+00 | 2.130467976 | 2.13E-02 | 1.00E-02 |
| **Lpcat3** | 4.87E-02 | 2.12E+00 | 2.122801411 | 1.39E-01 | 6.55E-02 |
| **Slc38a8** | 2.30E-02 | 2.12E+00 | 2.117062656 | 7.71E-01 | 3.64E-01 |
| **Gtpbp8** | 5.54E-03 | 2.11E+00 | 2.112082476 | 4.00E+00 | 1.89E+00 |
| **Fam89b** | 3.50E-02 | 2.11E+00 | 2.111279572 | 9.22E+00 | 4.36E+00 |
| **Fkbp14** | 4.18E-02 | 2.11E+00 | 2.111021981 | 4.82E-01 | 2.28E-01 |
| **Tfrc** | 2.83E-02 | 2.10E+00 | 2.104290516 | 5.23E-01 | 2.48E-01 |
| **Stox1** | 4.59E-02 | 2.10E+00 | 2.101286083 | 3.83E-02 | 1.82E-02 |
| **Pcbd1** | 7.40E-03 | 2.10E+00 | 2.101128997 | 2.45E+01 | 1.17E+01 |
| **BC017158** | 4.12E-02 | 2.10E+00 | 2.100660806 | 6.97E-01 | 3.32E-01 |
| **Slc25a10** | 4.26E-02 | 2.10E+00 | 2.100288174 | 7.12E-01 | 3.39E-01 |
| **Naprt** | 4.09E-02 | 2.10E+00 | 2.097118149 | 2.23E+00 | 1.06E+00 |
| **Mrps34** | 1.40E-02 | 2.09E+00 | 2.092866963 | 1.59E+01 | 7.61E+00 |
| **Psme2** | 1.22E-02 | 2.09E+00 | 2.091366461 | 3.88E+00 | 1.86E+00 |
| **Nrarp** | 3.32E-02 | 2.09E+00 | 2.090898703 | 2.35E+00 | 1.12E+00 |
| **Map3k3** | 1.71E-02 | 2.09E+00 | 2.088489385 | 2.70E-02 | 1.29E-02 |
| **Parp16** | 2.52E-02 | 2.09E+00 | 2.088085263 | 3.63E-01 | 1.74E-01 |
| **Hmgn2** | 7.76E-03 | 2.09E+00 | 2.087965918 | 2.69E+01 | 1.29E+01 |
| **Gna11** | 2.17E-02 | 2.09E+00 | 2.087592354 | 5.28E+00 | 2.53E+00 |
| **Atp6ap2** | 2.72E-02 | 2.09E+00 | 2.087481476 | 2.61E+00 | 1.25E+00 |
| **Tspan3** | 3.60E-02 | 2.08E+00 | 2.084706876 | 6.00E+00 | 2.88E+00 |
| **Rpl9-ps6** | 1.27E-02 | 2.08E+00 | 2.083750647 | 5.69E+01 | 2.73E+01 |
| **Ivns1abp** | 1.53E-02 | 2.08E+00 | 2.081793357 | 5.40E-01 | 2.59E-01 |
| **Actb** | 3.39E-03 | 2.08E+00 | 2.079890815 | 2.84E+01 | 1.37E+01 |
| **Slc25a39** | 1.73E-02 | 2.08E+00 | 2.078596358 | 1.05E+01 | 5.04E+00 |
| **Ric3** | 3.73E-02 | 2.08E+00 | 2.078018143 | 1.98E-01 | 9.52E-02 |
| **Kifap3** | 4.80E-02 | 2.08E+00 | 2.07792586 | 5.76E-02 | 2.77E-02 |
| **Glul** | 1.36E-02 | 2.08E+00 | 2.077047904 | 2.58E+00 | 1.24E+00 |
| **Srsf6** | 1.81E-02 | 2.08E+00 | 2.075265025 | 3.72E+00 | 1.79E+00 |
| **Ppp1r16b** | 4.99E-02 | 2.07E+00 | 2.065439027 | 3.28E-02 | 1.59E-02 |
| **Chrac1** | 4.80E-02 | 2.06E+00 | 2.06082709 | 1.74E+00 | 8.42E-01 |
| **Myg1** | 3.42E-02 | 2.06E+00 | 2.056071315 | 3.38E+00 | 1.65E+00 |
| **Wdcp** | 3.02E-02 | 2.05E+00 | 2.052681561 | 9.75E-01 | 4.75E-01 |
| **Psmd13** | 1.52E-02 | 2.05E+00 | 2.052135947 | 1.31E+00 | 6.40E-01 |
| **Proca1** | 2.99E-02 | 2.05E+00 | 2.047695926 | 1.88E+00 | 9.18E-01 |
| **Pnkp** | 4.59E-02 | 2.05E+00 | 2.046271805 | 1.96E+00 | 9.57E-01 |
| **Atp5g3** | 6.94E-03 | 2.05E+00 | 2.045431808 | 2.93E+01 | 1.43E+01 |
| **Setd1b** | 4.48E-02 | 2.04E+00 | 2.044580904 | 3.47E-01 | 1.70E-01 |
| **Sde2** | 1.11E-02 | 2.04E+00 | 2.043867724 | 1.66E+00 | 8.11E-01 |
| **Flot2** | 2.77E-02 | 2.04E+00 | 2.041983242 | 1.32E+00 | 6.46E-01 |
| **Slc7a6** | 4.87E-02 | 2.04E+00 | 2.039865398 | 1.76E-01 | 8.64E-02 |
| **Kbtbd4** | 2.30E-02 | 2.04E+00 | 2.03902904 | 8.42E+00 | 4.13E+00 |
| **Esf1** | 2.34E-02 | 2.04E+00 | 2.037657867 | 6.06E-01 | 2.97E-01 |
| **Gstz1** | 1.27E-02 | 2.04E+00 | 2.037341229 | 3.01E+00 | 1.48E+00 |
| **Eef1akmt1** | 9.70E-03 | 2.03E+00 | 2.033809427 | 1.82E+00 | 8.94E-01 |
| **Pus10** | 3.54E-02 | 2.03E+00 | 2.033312581 | 1.89E-01 | 9.32E-02 |
| **Endog** | 4.07E-02 | 2.03E+00 | 2.03298166 | 2.35E+01 | 1.15E+01 |
| **Spint2** | 2.57E-02 | 2.03E+00 | 2.031032264 | 3.55E+00 | 1.75E+00 |
| **Ppdpf** | 2.49E-02 | 2.03E+00 | 2.030829356 | 3.86E+01 | 1.90E+01 |
| **Fam186b** | 2.10E-02 | 2.03E+00 | 2.028202529 | 2.03E-02 | 1.00E-02 |
| **Bad** | 2.88E-02 | 2.02E+00 | 2.02483141 | 9.64E-01 | 4.76E-01 |
| **Tpm3** | 3.09E-02 | 2.02E+00 | 2.018817952 | 1.54E+00 | 7.63E-01 |
| **Tomm20** | 1.52E-02 | 2.01E+00 | 2.013430793 | 4.47E+00 | 2.22E+00 |
| **Ikbkb** | 2.04E-02 | 2.01E+00 | 2.008713596 | 2.09E+00 | 1.04E+00 |

**Down-regulated expressed genes between in MFN1^-/-^ oocytes compared to WT.**

| **Gene ID** | **FDR PValue** | **Ratio** | **Fold change** | **Mean normalized counts (KO)** | **Mean normalized counts (WT)** |
| --- | --- | --- | --- | --- | --- |
| **Bdkrb1** | 2.80E-02 | 4.98E-01 | -2.006271421 | 3.71E+00 | 7.45E+00 |
| **Esr2** | 2.25E-02 | 4.98E-01 | -2.007601789 | 9.29E-01 | 1.86E+00 |
| **Cnst** | 2.54E-02 | 4.97E-01 | -2.012002356 | 2.66E-01 | 5.36E-01 |
| **Zfp472** | 1.53E-02 | 4.96E-01 | -2.016362945 | 2.46E+00 | 4.95E+00 |
| **Tirap** | 3.93E-02 | 4.93E-01 | -2.026357687 | 1.92E-01 | 3.88E-01 |
| **Slx4ip** | 4.76E-02 | 4.92E-01 | -2.032169829 | 7.52E-02 | 1.53E-01 |
| **Ipmk** | 1.53E-02 | 4.92E-01 | -2.033690068 | 1.09E+00 | 2.22E+00 |
| **Fabp7** | 4.74E-02 | 4.91E-01 | -2.03592779 | 4.43E-01 | 9.03E-01 |
| **Chrng** | 4.09E-02 | 4.90E-01 | -2.039672856 | 9.24E-01 | 1.88E+00 |
| **Rasl2-9** | 7.03E-03 | 4.90E-01 | -2.042884117 | 6.76E+00 | 1.38E+01 |
| **Map3k7cl** | 2.92E-02 | 4.88E-01 | -2.048404245 | 1.22E-02 | 2.51E-02 |
| **Ttc41** | 4.28E-02 | 4.86E-01 | -2.059318481 | 1.43E-01 | 2.95E-01 |
| **1700025G04Rik** | 4.55E-02 | 4.85E-01 | -2.06055977 | 6.62E-02 | 1.36E-01 |
| **Crlf1** | 1.59E-02 | 4.85E-01 | -2.06062202 | 1.06E+00 | 2.18E+00 |
| **Igsf3** | 4.07E-02 | 4.83E-01 | -2.070166048 | 2.51E-01 | 5.20E-01 |
| **Gnrh1** | 2.86E-02 | 4.83E-01 | -2.072418116 | 9.61E+00 | 1.99E+01 |
| **Nfe2l2** | 7.66E-03 | 4.81E-01 | -2.077743583 | 5.90E-01 | 1.23E+00 |
| **Ccdc141** | 4.85E-02 | 4.80E-01 | -2.085396168 | 2.64E-02 | 5.51E-02 |
| **Zfp688** | 3.59E-03 | 4.79E-01 | -2.086401769 | 1.46E+00 | 3.06E+00 |
| **Calr3** | 4.00E-02 | 4.79E-01 | -2.088242245 | 1.70E+00 | 3.54E+00 |
| **Mdm4** | 1.71E-02 | 4.78E-01 | -2.092785093 | 1.24E+01 | 2.59E+01 |
| **Fbxw25** | 2.33E-02 | 4.75E-01 | -2.103315919 | 2.74E+00 | 5.76E+00 |
| **Gm13023** | 1.84E-02 | 4.75E-01 | -2.103994465 | 1.00E+02 | 2.10E+02 |
| **Mctp2** | 3.85E-02 | 4.74E-01 | -2.111060415 | 1.03E-01 | 2.17E-01 |
| **Abca12** | 2.80E-02 | 4.73E-01 | -2.113171132 | 1.36E-02 | 2.87E-02 |
| **Ifrd1** | 7.66E-03 | 4.73E-01 | -2.115764919 | 5.06E-01 | 1.07E+00 |
| **BC147527** | 1.71E-02 | 4.72E-01 | -2.116444943 | 7.31E-01 | 1.55E+00 |
| **Cpn1** | 4.75E-02 | 4.72E-01 | -2.117930701 | 4.36E-02 | 9.24E-02 |
| **Tmem265** | 4.29E-02 | 4.72E-01 | -2.119104796 | 3.05E-01 | 6.46E-01 |
| **Psg20** | 3.48E-02 | 4.72E-01 | -2.119652745 | 3.97E-01 | 8.41E-01 |
| **Klhl9** | 3.13E-02 | 4.71E-01 | -2.123911107 | 2.44E+00 | 5.19E+00 |
| **Aire** | 1.63E-02 | 4.69E-01 | -2.13081305 | 2.32E-01 | 4.94E-01 |
| **Ttpal** | 2.57E-02 | 4.69E-01 | -2.133239394 | 3.00E-01 | 6.39E-01 |
| **Cyb5a** | 8.88E-03 | 4.67E-01 | -2.140924283 | 4.97E+00 | 1.06E+01 |
| **Trim71** | 1.59E-02 | 4.66E-01 | -2.147472336 | 1.56E+00 | 3.34E+00 |
| **Dnah5** | 4.35E-02 | 4.64E-01 | -2.156892961 | 2.03E-02 | 4.39E-02 |
| **C1qb** | 1.95E-02 | 4.63E-01 | -2.158796036 | 4.25E+00 | 9.17E+00 |
| **Fn1** | 2.68E-02 | 4.61E-01 | -2.171193992 | 1.00E-01 | 2.17E-01 |
| **C1galt1** | 4.54E-02 | 4.60E-01 | -2.173574331 | 7.38E-02 | 1.60E-01 |
| **Gtf2ird1** | 1.55E-02 | 4.58E-01 | -2.181850737 | 3.70E-01 | 8.08E-01 |
| **Jak2** | 1.95E-02 | 4.55E-01 | -2.196792951 | 6.67E-01 | 1.47E+00 |
| **Lrriq4** | 2.83E-02 | 4.55E-01 | -2.19741047 | 1.17E-01 | 2.56E-01 |
| **Rora** | 3.93E-02 | 4.55E-01 | -2.199729325 | 5.98E-02 | 1.32E-01 |
| **Larp4b** | 4.30E-02 | 4.53E-01 | -2.205505996 | 2.73E-01 | 6.02E-01 |
| **Fam199x** | 4.93E-02 | 4.53E-01 | -2.206315003 | 8.24E-01 | 1.82E+00 |
| **Ccdc3** | 1.72E-02 | 4.53E-01 | -2.207311054 | 2.21E-01 | 4.87E-01 |
| **Myh2** | 1.93E-02 | 4.53E-01 | -2.209596511 | 7.04E-01 | 1.56E+00 |
| **Cfap45** | 1.45E-02 | 4.51E-01 | -2.216477422 | 5.00E-01 | 1.11E+00 |
| **Srgn** | 4.02E-02 | 4.51E-01 | -2.217765068 | 8.24E-01 | 1.83E+00 |
| **Tac1** | 2.27E-02 | 4.51E-01 | -2.217800962 | 2.73E-01 | 6.05E-01 |
| **Gm4985** | 6.94E-03 | 4.50E-01 | -2.22005296 | 1.51E+01 | 3.35E+01 |
| **Litaf** | 3.27E-02 | 4.50E-01 | -2.220097775 | 1.43E-01 | 3.18E-01 |
| **Fbxw4** | 7.66E-03 | 4.50E-01 | -2.221172693 | 6.26E-01 | 1.39E+00 |
| **Gdap1** | 3.55E-02 | 4.49E-01 | -2.228685654 | 1.03E+00 | 2.30E+00 |
| **Btg1-ps1** | 3.60E-02 | 4.48E-01 | -2.230198951 | 5.15E-01 | 1.15E+00 |
| **Gm5134** | 2.45E-02 | 4.48E-01 | -2.230215526 | 2.51E-01 | 5.59E-01 |
| **Nrp1** | 2.42E-02 | 4.47E-01 | -2.235410474 | 8.23E-02 | 1.84E-01 |
| **Arrdc5** | 3.85E-02 | 4.47E-01 | -2.238398728 | 2.57E-01 | 5.75E-01 |
| **Nt5c3** | 3.72E-02 | 4.46E-01 | -2.241659243 | 2.38E-01 | 5.33E-01 |
| **Rcl1** | 4.26E-03 | 4.45E-01 | -2.244958432 | 7.36E-01 | 1.65E+00 |
| **Il6st** | 1.58E-02 | 4.45E-01 | -2.246875851 | 1.79E-01 | 4.03E-01 |
| **Tmtc1** | 2.96E-02 | 4.45E-01 | -2.247692472 | 6.05E-02 | 1.36E-01 |
| **Dnajb4** | 2.04E-02 | 4.44E-01 | -2.250769613 | 3.49E+00 | 7.86E+00 |
| **Supt20** | 2.04E-02 | 4.44E-01 | -2.253834849 | 2.61E+00 | 5.89E+00 |
| **Stard5** | 2.87E-02 | 4.42E-01 | -2.261946121 | 3.20E-01 | 7.24E-01 |
| **Mettl7a2** | 9.69E-03 | 4.42E-01 | -2.262330888 | 1.56E+00 | 3.54E+00 |
| **Rftn2** | 4.60E-02 | 4.41E-01 | -2.266500363 | 5.87E-02 | 1.33E-01 |
| **Oog3** | 1.23E-02 | 4.41E-01 | -2.267145372 | 2.26E+02 | 5.12E+02 |
| **Kbtbd8os** | 4.56E-02 | 4.40E-01 | -2.272655856 | 4.63E-01 | 1.05E+00 |
| **AC158605.2** | 4.03E-02 | 4.40E-01 | -2.273917589 | 1.62E+00 | 3.69E+00 |
| **Ceacam20** | 3.46E-02 | 4.39E-01 | -2.275318438 | 8.89E-01 | 2.02E+00 |
| **Scin** | 1.66E-02 | 4.39E-01 | -2.278102989 | 1.51E-01 | 3.44E-01 |
| **Kcnn2** | 4.52E-02 | 4.37E-01 | -2.290867681 | 7.13E-02 | 1.63E-01 |
| **Sbk3** | 1.66E-02 | 4.36E-01 | -2.291428023 | 3.67E-01 | 8.41E-01 |
| **Cd101** | 2.96E-02 | 4.36E-01 | -2.294282363 | 1.36E-02 | 3.12E-02 |
| **Mcu** | 2.88E-02 | 4.36E-01 | -2.295956794 | 8.76E-02 | 2.01E-01 |
| **Lrriq1** | 3.09E-02 | 4.34E-01 | -2.302725647 | 1.34E-02 | 3.09E-02 |
| **Esrrb** | 1.17E-02 | 4.29E-01 | -2.333480543 | 5.14E-01 | 1.20E+00 |
| **Ehf** | 2.08E-02 | 4.28E-01 | -2.335045645 | 3.43E+00 | 8.01E+00 |
| **Myh3** | 4.80E-02 | 4.27E-01 | -2.33993881 | 2.82E-02 | 6.60E-02 |
| **Azin1** | 2.24E-02 | 4.27E-01 | -2.340406818 | 3.27E-01 | 7.64E-01 |
| **Pfn4** | 2.68E-02 | 4.27E-01 | -2.340974698 | 9.82E-01 | 2.30E+00 |
| **Iqcg** | 3.84E-02 | 4.27E-01 | -2.343235168 | 5.54E-01 | 1.30E+00 |
| **Rsrp1** | 1.61E-02 | 4.24E-01 | -2.356391263 | 4.37E+00 | 1.03E+01 |
| **Tex9** | 2.93E-02 | 4.24E-01 | -2.356435389 | 4.41E-02 | 1.04E-01 |
| **Bbs7** | 2.22E-02 | 4.24E-01 | -2.356591011 | 1.39E-01 | 3.27E-01 |
| **Zfp516** | 4.85E-02 | 4.24E-01 | -2.360219263 | 2.90E-02 | 6.83E-02 |
| **Dpy19l1** | 1.66E-02 | 4.24E-01 | -2.360542079 | 1.38E-01 | 3.26E-01 |
| **Sptlc3** | 3.75E-02 | 4.23E-01 | -2.361946596 | 2.65E-02 | 6.27E-02 |
| **Spire1** | 1.62E-02 | 4.23E-01 | -2.362536361 | 3.42E-01 | 8.09E-01 |
| **Xlr5b** | 1.42E-02 | 4.23E-01 | -2.362734245 | 4.91E-01 | 1.16E+00 |
| **Slco5a1** | 3.31E-02 | 4.21E-01 | -2.376517404 | 2.38E-02 | 5.65E-02 |
| **Sfxn4** | 3.13E-02 | 4.21E-01 | -2.377537451 | 1.12E-01 | 2.65E-01 |
| **Cpm** | 3.13E-02 | 4.20E-01 | -2.380517247 | 1.39E-01 | 3.30E-01 |
| **C130060K24Rik** | 3.00E-02 | 4.20E-01 | -2.381761693 | 4.55E-01 | 1.08E+00 |
| **Rhoh** | 1.73E-02 | 4.19E-01 | -2.387802481 | 1.05E+00 | 2.51E+00 |
| **Lgals9** | 4.33E-02 | 4.18E-01 | -2.394036888 | 3.27E-02 | 7.83E-02 |
| **Fkbp5** | 1.95E-02 | 4.17E-01 | -2.400500157 | 8.81E-02 | 2.11E-01 |
| **Olfr624** | 2.20E-02 | 4.16E-01 | -2.402310763 | 7.84E-01 | 1.88E+00 |
| **Aph1c** | 6.30E-03 | 4.16E-01 | -2.403288298 | 1.92E+00 | 4.61E+00 |
| **Sebox** | 2.92E-02 | 4.16E-01 | -2.403534902 | 3.59E+01 | 8.63E+01 |
| **Rcsd1** | 4.87E-02 | 4.16E-01 | -2.403739495 | 3.20E-02 | 7.68E-02 |
| **Kif5c** | 2.78E-02 | 4.15E-01 | -2.411486859 | 3.93E-02 | 9.47E-02 |
| **Nkrf** | 2.08E-02 | 4.12E-01 | -2.424913585 | 2.48E-01 | 6.02E-01 |
| **Bmpr2** | 4.52E-02 | 4.12E-01 | -2.425262918 | 8.35E-02 | 2.03E-01 |
| **Myl4** | 4.46E-02 | 4.11E-01 | -2.430755729 | 7.38E-02 | 1.79E-01 |
| **Reg1** | 4.99E-02 | 4.11E-01 | -2.435957348 | 2.15E-01 | 5.25E-01 |
| **Vmn2r26** | 4.21E-02 | 4.10E-01 | -2.43743258 | 1.00E-02 | 2.44E-02 |
| **Epha3** | 4.61E-02 | 4.09E-01 | -2.444759576 | 1.21E-02 | 2.96E-02 |
| **Sspn** | 1.03E-02 | 4.08E-01 | -2.449272947 | 2.36E-01 | 5.79E-01 |
| **Cd180** | 2.13E-02 | 4.08E-01 | -2.451434115 | 1.40E-01 | 3.43E-01 |
| **Iglc2** | 1.51E-02 | 4.04E-01 | -2.473621964 | 4.32E+00 | 1.07E+01 |
| **Aamdc** | 2.73E-02 | 4.04E-01 | -2.477648742 | 1.65E-01 | 4.08E-01 |
| **Stxbp6** | 2.64E-02 | 4.01E-01 | -2.492332267 | 1.04E-01 | 2.58E-01 |
| **Ago4** | 2.41E-02 | 4.01E-01 | -2.49356966 | 1.75E-02 | 4.36E-02 |
| **Got1l1** | 4.71E-02 | 4.01E-01 | -2.495143296 | 4.38E-02 | 1.09E-01 |
| **Mei4** | 4.27E-02 | 4.01E-01 | -2.496048062 | 1.96E-02 | 4.88E-02 |
| **Ttll7** | 4.97E-02 | 4.00E-01 | -2.498544247 | 1.58E-02 | 3.96E-02 |
| **Cfap53** | 3.16E-02 | 3.99E-01 | -2.509141305 | 1.97E-01 | 4.94E-01 |
| **Abhd2** | 4.27E-02 | 3.98E-01 | -2.512778295 | 2.13E-02 | 5.36E-02 |
| **Lrguk** | 3.92E-02 | 3.98E-01 | -2.513644277 | 3.55E-02 | 8.91E-02 |
| **Fam84b** | 3.69E-02 | 3.97E-01 | -2.516025816 | 1.85E-01 | 4.65E-01 |
| **Kbtbd7** | 2.92E-02 | 3.97E-01 | -2.516934174 | 7.37E+00 | 1.85E+01 |
| **Slc7a11** | 2.17E-02 | 3.97E-01 | -2.517047266 | 5.79E-02 | 1.46E-01 |
| **Fam229b** | 1.51E-02 | 3.97E-01 | -2.517442843 | 2.74E+00 | 6.89E+00 |
| **Lonrf3** | 8.18E-03 | 3.96E-01 | -2.522320119 | 9.88E-01 | 2.49E+00 |
| **Serpinb1c** | 2.76E-02 | 3.95E-01 | -2.529161965 | 2.52E-01 | 6.36E-01 |
| **Sepsecs** | 2.64E-02 | 3.95E-01 | -2.529718575 | 4.18E-01 | 1.06E+00 |
| **Trim38** | 4.57E-02 | 3.95E-01 | -2.530373296 | 1.14E-01 | 2.88E-01 |
| **Mkrn2os** | 1.71E-02 | 3.95E-01 | -2.532528706 | 3.32E-01 | 8.42E-01 |
| **Zmat3** | 2.64E-02 | 3.94E-01 | -2.536494373 | 1.42E-02 | 3.61E-02 |
| **Daw1** | 2.41E-02 | 3.94E-01 | -2.540530958 | 3.99E-01 | 1.01E+00 |
| **Pzp** | 4.52E-02 | 3.93E-01 | -2.541347758 | 1.79E-02 | 4.55E-02 |
| **Il17b** | 4.69E-02 | 3.93E-01 | -2.541406444 | 3.80E-01 | 9.66E-01 |
| **Egfl6** | 2.45E-02 | 3.92E-01 | -2.549747114 | 1.62E-01 | 4.13E-01 |
| **Styk1** | 3.27E-02 | 3.92E-01 | -2.551267423 | 2.35E-01 | 6.00E-01 |
| **Thegl** | 2.26E-02 | 3.91E-01 | -2.556667964 | 1.95E-01 | 5.00E-01 |
| **Rab8b** | 2.92E-02 | 3.90E-01 | -2.562577849 | 1.26E-01 | 3.23E-01 |
| **Zfp978** | 4.08E-02 | 3.90E-01 | -2.564583132 | 3.57E-01 | 9.16E-01 |
| **Opalin** | 1.89E-02 | 3.89E-01 | -2.567753878 | 9.51E-01 | 2.44E+00 |
| **Fmnl3** | 9.49E-03 | 3.89E-01 | -2.570563944 | 6.40E-01 | 1.65E+00 |
| **Slc7a2** | 4.61E-02 | 3.88E-01 | -2.57600031 | 2.45E-01 | 6.30E-01 |
| **Wwp1** | 4.68E-02 | 3.88E-01 | -2.577381935 | 6.57E-02 | 1.69E-01 |
| **Plekhb2** | 1.75E-02 | 3.88E-01 | -2.578866641 | 9.56E-02 | 2.46E-01 |
| **Gm13078** | 3.47E-02 | 3.87E-01 | -2.582993089 | 1.00E-02 | 2.58E-02 |
| **Sclt1** | 2.64E-02 | 3.87E-01 | -2.584419628 | 1.26E-01 | 3.27E-01 |
| **Hoxa7** | 3.67E-02 | 3.86E-01 | -2.587907639 | 1.51E+00 | 3.92E+00 |
| **Sult2a7** | 3.33E-02 | 3.86E-01 | -2.588122133 | 1.12E-02 | 2.89E-02 |
| **D330045A20Rik** | 4.94E-02 | 3.82E-01 | -2.618828567 | 3.26E-02 | 8.55E-02 |
| **Rgs10** | 1.84E-02 | 3.82E-01 | -2.618892094 | 9.02E-01 | 2.36E+00 |
| **Pir** | 4.85E-02 | 3.78E-01 | -2.643067593 | 2.29E-02 | 6.06E-02 |
| **Plce1** | 2.51E-02 | 3.77E-01 | -2.650815389 | 1.83E-01 | 4.84E-01 |
| **2300002M23Rik** | 4.11E-02 | 3.77E-01 | -2.656010135 | 1.61E+00 | 4.27E+00 |
| **Lrrc34** | 4.12E-02 | 3.75E-01 | -2.663857772 | 1.09E-01 | 2.92E-01 |
| **Abcb1b** | 2.49E-02 | 3.75E-01 | -2.668394742 | 3.92E-01 | 1.05E+00 |
| **Ccdc180** | 2.36E-02 | 3.74E-01 | -2.67600198 | 9.48E-02 | 2.54E-01 |
| **Vstm5** | 2.96E-02 | 3.73E-01 | -2.684029032 | 1.47E-02 | 3.95E-02 |
| **Sptlc2** | 2.32E-02 | 3.72E-01 | -2.685540309 | 3.34E-01 | 8.96E-01 |
| **Ttc6** | 2.99E-02 | 3.70E-01 | -2.700436668 | 3.76E-02 | 1.01E-01 |
| **Fbxo17** | 1.71E-02 | 3.70E-01 | -2.701904766 | 1.23E-01 | 3.32E-01 |
| **Pcyt1b** | 2.82E-02 | 3.70E-01 | -2.70428182 | 7.27E-02 | 1.97E-01 |
| **Csf1** | 3.30E-02 | 3.69E-01 | -2.709285305 | 1.89E-01 | 5.13E-01 |
| **Lrrtm2** | 4.35E-02 | 3.68E-01 | -2.715119852 | 5.03E-01 | 1.37E+00 |
| **Cd300c** | 1.41E-02 | 3.66E-01 | -2.731715143 | 1.52E-01 | 4.15E-01 |
| **Efcab3** | 2.45E-02 | 3.66E-01 | -2.734666127 | 1.16E-01 | 3.17E-01 |
| **Fam213a** | 2.53E-02 | 3.65E-01 | -2.738357637 | 1.25E-01 | 3.41E-01 |
| **Oit1** | 1.71E-02 | 3.64E-01 | -2.750817804 | 1.09E-01 | 2.99E-01 |
| **Il23r** | 4.76E-02 | 3.63E-01 | -2.751300983 | 9.30E-02 | 2.56E-01 |
| **Parp9** | 3.29E-02 | 3.62E-01 | -2.762042182 | 1.36E-02 | 3.76E-02 |
| **Tctex1d1** | 2.68E-02 | 3.61E-01 | -2.771687276 | 1.97E+00 | 5.45E+00 |
| **Myoc** | 1.99E-02 | 3.59E-01 | -2.781729093 | 1.32E-01 | 3.66E-01 |
| **Pnp2** | 2.17E-02 | 3.59E-01 | -2.785729547 | 4.14E-01 | 1.15E+00 |
| **Uaca** | 3.22E-02 | 3.59E-01 | -2.789221279 | 2.06E-02 | 5.75E-02 |
| **Crybg1** | 1.95E-02 | 3.58E-01 | -2.793419476 | 4.12E-02 | 1.15E-01 |
| **Prss8** | 3.52E-02 | 3.57E-01 | -2.80069702 | 7.81E-01 | 2.19E+00 |
| **Nlrc4** | 4.09E-02 | 3.57E-01 | -2.804533085 | 1.73E-01 | 4.86E-01 |
| **Nyap2** | 4.60E-02 | 3.56E-01 | -2.812017316 | 1.52E-02 | 4.28E-02 |
| **Zfp708** | 3.39E-02 | 3.54E-01 | -2.821491159 | 2.81E-01 | 7.94E-01 |
| **Neil3** | 1.92E-02 | 3.54E-01 | -2.825945632 | 1.48E+00 | 4.18E+00 |
| **Entpd1** | 4.76E-02 | 3.52E-01 | -2.838119226 | 6.55E-02 | 1.86E-01 |
| **Lrmp** | 2.29E-02 | 3.52E-01 | -2.838720177 | 1.26E-02 | 3.57E-02 |
| **Fam84a** | 2.62E-02 | 3.52E-01 | -2.842534878 | 1.40E+00 | 3.99E+00 |
| **Gata3** | 4.10E-02 | 3.52E-01 | -2.844432785 | 3.03E-01 | 8.61E-01 |
| **Fmo5** | 3.39E-02 | 3.46E-01 | -2.886126509 | 1.61E-01 | 4.64E-01 |
| **Arhgef28** | 3.75E-02 | 3.44E-01 | -2.909171509 | 2.28E-02 | 6.62E-02 |
| **Nfkbie** | 1.90E-02 | 3.42E-01 | -2.925096375 | 2.60E-01 | 7.60E-01 |
| **Tmem260** | 2.04E-02 | 3.41E-01 | -2.929969884 | 6.69E-02 | 1.96E-01 |
| **Nostrin** | 4.32E-02 | 3.40E-01 | -2.940224863 | 7.08E-02 | 2.08E-01 |
| **Lrp6** | 3.42E-02 | 3.40E-01 | -2.941362502 | 6.66E-02 | 1.96E-01 |
| **Abcb4** | 2.91E-02 | 3.40E-01 | -2.94234088 | 1.48E-01 | 4.35E-01 |
| **4930544G11Rik** | 4.48E-02 | 3.40E-01 | -2.945116427 | 3.19E-01 | 9.40E-01 |
| **4930469K13Rik** | 4.45E-02 | 3.39E-01 | -2.951458516 | 2.01E-02 | 5.92E-02 |
| **Zfp937** | 2.92E-02 | 3.39E-01 | -2.953100104 | 1.17E-01 | 3.44E-01 |
| **2810030D12Rik** | 3.29E-02 | 3.39E-01 | -2.953882304 | 1.21E-01 | 3.58E-01 |
| **Sult1d1** | 2.36E-02 | 3.34E-01 | -2.993972751 | 1.00E-02 | 2.99E-02 |
| **Mroh7** | 1.59E-02 | 3.34E-01 | -2.996081489 | 4.78E-02 | 1.43E-01 |
| **Glis1** | 1.99E-02 | 3.34E-01 | -2.997573545 | 3.83E-02 | 1.15E-01 |
| **Psg17** | 3.40E-02 | 3.33E-01 | -3.005455266 | 1.57E+00 | 4.71E+00 |
| **Dsg1a** | 1.61E-02 | 3.30E-01 | -3.026434499 | 1.42E-02 | 4.29E-02 |
| **1810041L15Rik** | 4.07E-02 | 3.29E-01 | -3.037211062 | 1.82E-02 | 5.52E-02 |
| **Lcp2** | 3.48E-02 | 3.28E-01 | -3.044905523 | 3.12E-02 | 9.50E-02 |
| **Sh3rf2** | 4.18E-02 | 3.28E-01 | -3.052824517 | 1.47E-01 | 4.48E-01 |
| **Gm6526** | 2.96E-02 | 3.26E-01 | -3.066717958 | 8.18E-02 | 2.51E-01 |
| **Ube2u** | 4.73E-02 | 3.25E-01 | -3.078701404 | 3.82E-02 | 1.18E-01 |
| **Zfp157** | 2.33E-02 | 3.24E-01 | -3.084117995 | 5.72E-02 | 1.76E-01 |
| **Epsti1** | 2.61E-02 | 3.23E-01 | -3.099495367 | 1.45E-01 | 4.50E-01 |
| **Cela1** | 1.71E-02 | 3.22E-01 | -3.109936382 | 4.00E-01 | 1.24E+00 |
| **Ddx58** | 3.38E-02 | 3.19E-01 | -3.131976135 | 3.27E-01 | 1.03E+00 |
| **BC048562** | 3.83E-02 | 3.18E-01 | -3.140555951 | 1.35E-02 | 4.25E-02 |
| **Tfap2b** | 4.57E-02 | 3.18E-01 | -3.145233719 | 1.78E-02 | 5.61E-02 |
| **Gm14288** | 3.81E-02 | 3.17E-01 | -3.149666927 | 1.88E-01 | 5.93E-01 |
| **Slc17a2** | 3.98E-02 | 3.17E-01 | -3.156236596 | 7.12E-02 | 2.25E-01 |
| **Cldn34b2** | 2.76E-02 | 3.17E-01 | -3.157440321 | 2.40E+00 | 7.59E+00 |
| **Glis3** | 1.76E-02 | 3.14E-01 | -3.181932124 | 1.42E-02 | 4.51E-02 |
| **Tac2** | 1.15E-02 | 3.14E-01 | -3.189009783 | 1.59E+00 | 5.06E+00 |
| **Bhlhe41** | 1.61E-02 | 3.13E-01 | -3.197116894 | 1.72E-01 | 5.50E-01 |
| **Vmn2r25** | 2.26E-02 | 3.12E-01 | -3.210252322 | 5.77E-02 | 1.85E-01 |
| **Bmp5** | 2.24E-02 | 3.09E-01 | -3.238555053 | 1.58E-01 | 5.11E-01 |
| **Eras** | 1.62E-02 | 3.09E-01 | -3.240250298 | 2.43E-01 | 7.87E-01 |
| **Ms4a1** | 4.43E-02 | 3.09E-01 | -3.240311901 | 1.18E+00 | 3.82E+00 |
| **Egfr** | 2.33E-02 | 3.08E-01 | -3.242183912 | 9.80E-02 | 3.18E-01 |
| **Olfr214** | 2.19E-02 | 3.04E-01 | -3.285436453 | 7.04E-01 | 2.31E+00 |
| **Cd1d1** | 2.58E-02 | 3.03E-01 | -3.295108156 | 4.88E-01 | 1.61E+00 |
| **Gcnt2** | 3.27E-02 | 3.02E-01 | -3.30684318 | 1.24E-02 | 4.10E-02 |
| **Cd5** | 2.33E-02 | 3.02E-01 | -3.306871103 | 3.01E-01 | 9.95E-01 |
| **Runx1t1** | 3.28E-02 | 3.02E-01 | -3.308427737 | 5.01E-02 | 1.66E-01 |
| **Gm7073** | 2.33E-02 | 2.99E-01 | -3.339675314 | 2.16E-02 | 7.23E-02 |
| **Ccnb3** | 3.21E-02 | 2.98E-01 | -3.360889649 | 5.04E-02 | 1.70E-01 |
| **Akr1b8** | 2.36E-02 | 2.97E-01 | -3.361903655 | 7.39E-01 | 2.49E+00 |
| **Mbd3l2** | 1.87E-02 | 2.97E-01 | -3.363146413 | 7.71E-01 | 2.59E+00 |
| **Rmdn2** | 3.04E-02 | 2.95E-01 | -3.388791331 | 2.38E-02 | 8.06E-02 |
| **Slc26a9** | 9.89E-03 | 2.94E-01 | -3.406287215 | 8.76E-02 | 2.98E-01 |
| **Hbq1a** | 4.95E-02 | 2.93E-01 | -3.412483544 | 2.36E+00 | 8.07E+00 |
| **Gramd1c** | 3.21E-02 | 2.93E-01 | -3.415829041 | 4.53E-02 | 1.55E-01 |
| **Slc15a5** | 4.36E-02 | 2.90E-01 | -3.448160554 | 1.01E-01 | 3.48E-01 |
| **Cpa2** | 2.68E-02 | 2.89E-01 | -3.46295794 | 1.01E-01 | 3.51E-01 |
| **Oas1f** | 2.41E-02 | 2.88E-01 | -3.470145744 | 1.60E+00 | 5.55E+00 |
| **Myh11** | 4.39E-02 | 2.88E-01 | -3.471399503 | 8.79E-02 | 3.05E-01 |
| **Mmp24** | 4.61E-02 | 2.86E-01 | -3.500983298 | 1.86E-02 | 6.52E-02 |
| **Rgsl1** | 3.38E-02 | 2.82E-01 | -3.542565398 | 3.79E-02 | 1.34E-01 |
| **Ttc12** | 1.86E-02 | 2.81E-01 | -3.562497256 | 1.14E-01 | 4.07E-01 |
| **Ildr1** | 1.51E-02 | 2.81E-01 | -3.56287128 | 3.58E-01 | 1.27E+00 |
| **Gm7271** | 1.51E-02 | 2.80E-01 | -3.566163059 | 3.81E-02 | 1.36E-01 |
| **Mrap** | 1.18E-02 | 2.79E-01 | -3.58815875 | 1.52E+00 | 5.44E+00 |
| **Olfr543** | 4.19E-02 | 2.78E-01 | -3.598370244 | 1.71E-02 | 6.15E-02 |
| **Stxbp5l** | 2.78E-02 | 2.77E-01 | -3.605806985 | 2.45E-02 | 8.83E-02 |
| **Nckap1l** | 3.53E-02 | 2.76E-01 | -3.619863793 | 6.62E-02 | 2.40E-01 |
| **Asb13** | 3.17E-02 | 2.76E-01 | -3.629272437 | 7.26E-02 | 2.64E-01 |
| **Adamts4** | 3.22E-02 | 2.74E-01 | -3.643112489 | 7.01E-02 | 2.55E-01 |
| **Spocd1** | 2.31E-02 | 2.73E-01 | -3.656779336 | 9.16E-02 | 3.35E-01 |
| **Mroh3** | 1.73E-02 | 2.73E-01 | -3.658052589 | 6.30E-02 | 2.30E-01 |
| **Actg2** | 4.94E-02 | 2.71E-01 | -3.683677256 | 6.21E-02 | 2.29E-01 |
| **Trib2** | 3.47E-02 | 2.70E-01 | -3.706526938 | 2.48E-02 | 9.20E-02 |
| **Gm15128** | 1.95E-02 | 2.69E-01 | -3.719498211 | 2.43E-02 | 9.04E-02 |
| **Trav7n-6** | 4.10E-02 | 2.68E-01 | -3.728498812 | 6.00E-01 | 2.24E+00 |
| **Fam107b** | 1.53E-02 | 2.68E-01 | -3.733696409 | 1.68E-01 | 6.29E-01 |
| **Sh3bp4** | 1.20E-02 | 2.67E-01 | -3.740201928 | 1.60E-02 | 5.98E-02 |
| **Pdzph1** | 2.69E-02 | 2.64E-01 | -3.787055378 | 1.78E-01 | 6.73E-01 |
| **Slc2a12** | 7.66E-03 | 2.63E-01 | -3.801413852 | 7.79E-02 | 2.96E-01 |
| **Jam2** | 2.45E-02 | 2.62E-01 | -3.819719136 | 1.96E-01 | 7.50E-01 |
| **Cdh17** | 4.85E-02 | 2.61E-01 | -3.835833047 | 3.58E-02 | 1.37E-01 |
| **Ptgs2** | 2.19E-02 | 2.61E-01 | -3.837752107 | 1.00E-02 | 3.84E-02 |
| **Krt27** | 1.51E-02 | 2.60E-01 | -3.846516333 | 1.00E-02 | 3.85E-02 |
| **Cldn34d** | 9.39E-03 | 2.58E-01 | -3.873805467 | 2.79E-01 | 1.08E+00 |
| **Plek** | 2.62E-02 | 2.57E-01 | -3.892846292 | 2.21E-02 | 8.60E-02 |
| **Tslp** | 4.74E-02 | 2.57E-01 | -3.895290666 | 6.75E-02 | 2.63E-01 |
| **Olfr1463** | 3.89E-02 | 2.56E-01 | -3.901437898 | 5.54E-02 | 2.16E-01 |
| **Sec16b** | 1.93E-02 | 2.54E-01 | -3.938190397 | 1.72E-02 | 6.76E-02 |
| **Hectd2** | 3.37E-02 | 2.53E-01 | -3.94820155 | 3.95E-02 | 1.56E-01 |
| **Plpp1** | 1.61E-02 | 2.53E-01 | -3.952778669 | 1.26E-01 | 4.99E-01 |
| **Lrrtm3** | 3.14E-02 | 2.53E-01 | -3.957789145 | 2.19E-02 | 8.65E-02 |
| **Cnga1** | 2.29E-02 | 2.52E-01 | -3.96919897 | 2.84E-02 | 1.13E-01 |
| **Olfr878** | 3.74E-02 | 2.52E-01 | -3.970878281 | 9.11E-01 | 3.62E+00 |
| **Hc** | 1.63E-02 | 2.51E-01 | -3.978293795 | 1.31E-02 | 5.19E-02 |
| **Elovl7** | 2.24E-02 | 2.51E-01 | -3.980609017 | 2.84E-01 | 1.13E+00 |
| **Ankrd33b** | 1.71E-02 | 2.45E-01 | -4.076387577 | 4.99E-02 | 2.03E-01 |
| **Mrc1** | 4.18E-02 | 2.45E-01 | -4.07730616 | 2.73E-02 | 1.11E-01 |
| **Nipal1** | 1.15E-02 | 2.43E-01 | -4.122276897 | 7.15E-02 | 2.95E-01 |
| **Trps1** | 3.74E-02 | 2.39E-01 | -4.177359618 | 4.22E-02 | 1.76E-01 |
| **Mpped1** | 1.75E-02 | 2.39E-01 | -4.186420266 | 4.80E-02 | 2.01E-01 |
| **Prss56** | 6.90E-03 | 2.36E-01 | -4.23011327 | 1.47E-01 | 6.23E-01 |
| **Fam49a** | 2.82E-02 | 2.36E-01 | -4.24322541 | 3.03E-02 | 1.29E-01 |
| **Havcr1** | 1.76E-02 | 2.33E-01 | -4.285602177 | 3.33E-02 | 1.43E-01 |
| **4930522H14Rik** | 4.96E-02 | 2.33E-01 | -4.286862557 | 5.64E-02 | 2.42E-01 |
| **Nedd4l** | 2.57E-02 | 2.31E-01 | -4.327659313 | 1.17E-01 | 5.06E-01 |
| **Cxcr3** | 3.55E-02 | 2.30E-01 | -4.356511441 | 1.66E+00 | 7.22E+00 |
| **Popdc2** | 1.53E-02 | 2.26E-01 | -4.420522544 | 2.54E-01 | 1.12E+00 |
| **Dab2** | 2.71E-02 | 2.26E-01 | -4.433870176 | 7.19E-02 | 3.19E-01 |
| **Crabp2** | 2.52E-02 | 2.25E-01 | -4.445544304 | 8.46E+00 | 3.76E+01 |
| **Nfkbiz** | 1.01E-02 | 2.20E-01 | -4.537552128 | 3.49E-02 | 1.58E-01 |
| **1700019D03Rik** | 3.18E-02 | 2.18E-01 | -4.592523918 | 5.45E-02 | 2.50E-01 |
| **Aldh1a2** | 1.97E-02 | 2.15E-01 | -4.652717581 | 1.44E-01 | 6.68E-01 |
| **Gpr142** | 1.53E-02 | 2.14E-01 | -4.666493078 | 1.00E-02 | 4.67E-02 |
| **Olfr283** | 4.91E-02 | 2.14E-01 | -4.670297669 | 1.62E-01 | 7.56E-01 |
| **Gipc2** | 9.70E-03 | 2.09E-01 | -4.783655461 | 1.10E-01 | 5.27E-01 |
| **Rgn** | 1.73E-02 | 2.08E-01 | -4.810596132 | 9.78E-01 | 4.70E+00 |
| **Tnip3** | 2.83E-02 | 2.07E-01 | -4.822819097 | 3.29E-02 | 1.59E-01 |
| **Slc43a1** | 2.83E-02 | 2.03E-01 | -4.933677621 | 2.75E-02 | 1.36E-01 |
| **A630095E13Rik** | 3.79E-02 | 1.99E-01 | -5.015436708 | 4.80E-01 | 2.41E+00 |
| **Got1** | 4.26E-03 | 1.99E-01 | -5.03374279 | 4.98E-01 | 2.50E+00 |
| **12-Sep** | 3.13E-02 | 1.99E-01 | -5.035483684 | 1.58E-01 | 7.97E-01 |
| **Nmu** | 2.43E-02 | 1.97E-01 | -5.066895735 | 7.51E-02 | 3.81E-01 |
| **Ednrb** | 2.19E-02 | 1.96E-01 | -5.092635137 | 5.91E-02 | 3.01E-01 |
| **Fbxo40** | 2.45E-02 | 1.95E-01 | -5.120951044 | 4.53E-01 | 2.32E+00 |
| **Tekt4** | 1.20E-02 | 1.94E-01 | -5.151610798 | 2.41E-01 | 1.24E+00 |
| **Gm20594** | 7.02E-03 | 1.93E-01 | -5.173805458 | 2.78E+01 | 1.44E+02 |
| **Dirc2** | 1.17E-02 | 1.91E-01 | -5.236011248 | 2.71E-02 | 1.42E-01 |
| **Batf3** | 1.59E-02 | 1.91E-01 | -5.239732683 | 1.49E-01 | 7.79E-01 |
| **Igf1os** | 3.40E-02 | 1.89E-01 | -5.292415734 | 8.10E-02 | 4.29E-01 |
| **Lbx1** | 1.59E-02 | 1.86E-01 | -5.381012322 | 2.52E-01 | 1.35E+00 |
| **Uts2r** | 2.78E-02 | 1.73E-01 | -5.77436487 | 6.88E-02 | 3.97E-01 |
| **Slc4a3** | 1.84E-02 | 1.68E-01 | -5.956314305 | 3.65E-02 | 2.18E-01 |
| **Sptssb** | 2.21E-02 | 1.61E-01 | -6.207283322 | 6.04E-02 | 3.75E-01 |
| **Sgo2b** | 3.75E-02 | 1.60E-01 | -6.260691082 | 4.78E-02 | 2.99E-01 |
| **Fabp6** | 8.05E-03 | 1.59E-01 | -6.276171239 | 1.00E-02 | 6.28E-02 |
| **Tuba3b** | 8.84E-03 | 1.57E-01 | -6.357482673 | 3.12E-01 | 1.99E+00 |
| **Triml1** | 3.66E-02 | 1.56E-01 | -6.430238993 | 2.08E-02 | 1.34E-01 |
| **Olfr811** | 2.03E-02 | 1.52E-01 | -6.564185275 | 1.00E-02 | 6.56E-02 |
| **Ctsm** | 4.36E-02 | 1.45E-01 | -6.881256033 | 1.00E-02 | 6.88E-02 |
| **Pabpc6** | 2.33E-02 | 1.45E-01 | -6.913998176 | 1.93E-02 | 1.34E-01 |
| **Prl8a2** | 3.09E-02 | 1.31E-01 | -7.635551555 | 1.32E-01 | 1.01E+00 |
| **Cela2a** | 2.36E-02 | 1.22E-01 | -8.169955247 | 3.28E-02 | 2.68E-01 |
| **Krt12** | 4.47E-02 | 1.22E-01 | -8.189750108 | 1.86E+00 | 1.53E+01 |
| **BC051665** | 2.42E-02 | 1.19E-01 | -8.383778788 | 2.34E-02 | 1.96E-01 |
| **D330020A13Rik** | 4.10E-02 | 1.18E-01 | -8.48481588 | 4.38E-02 | 3.72E-01 |
| **Lalba** | 3.74E-02 | 1.17E-01 | -8.552874326 | 4.87E-02 | 4.16E-01 |
| **Spata19** | 2.17E-02 | 1.15E-01 | -8.67195798 | 1.00E-02 | 8.67E-02 |
| **Cryba2** | 1.20E-02 | 1.11E-01 | -9.015485117 | 1.00E-02 | 9.02E-02 |
| **Mfn1** | 2.04E-03 | 9.36E-02 | -10.68361679 | 5.77E-02 | 6.16E-01 |
| **Gpr84** | 3.92E-02 | 8.85E-02 | -11.29497219 | 2.64E-02 | 2.98E-01 |
| **Gpr12** | 1.63E-02 | 8.47E-02 | -11.81281333 | 2.05E-02 | 2.43E-01 |
| **4930583I09Rik** | 1.31E-02 | 7.44E-02 | -13.44057148 | 1.00E-02 | 1.34E-01 |
| **Apon** | 8.05E-03 | 7.23E-02 | -13.83306099 | 1.00E-02 | 1.38E-01 |
| **Cd200** | 1.74E-02 | 7.01E-02 | -14.27088627 | 3.48E-02 | 4.96E-01 |
| **Pih1d3** | 1.65E-02 | 5.67E-02 | -17.63228212 | 1.00E-02 | 1.76E-01 |
| **Gm2564** | 8.16E-03 | 4.15E-02 | -24.09694047 | 1.02E-02 | 2.45E-01 |
| **CT009546.1** | 3.29E-03 | 3.74E-02 | -26.7393047 | 1.00E-02 | 2.67E-01 |
